# Ensemble Modeling of the Likely Public Health Impact of a Pre-Erythrocytic Malaria Vaccine

**DOI:** 10.1371/journal.pmed.1001157

**Published:** 2012-01-17

**Authors:** Thomas Smith, Amanda Ross, Nicolas Maire, Nakul Chitnis, Alain Studer, Diggory Hardy, Alan Brooks, Melissa Penny, Marcel Tanner

**Affiliations:** 1 Swiss Tropical and Public Health Institute, Basel, Switzerland; 2 University of Basel, Basel, Switzerland; St. George's, University of London, United Kingdom

## Abstract

**Background:**

The RTS,S malaria vaccine may soon be licensed. Models of impact of such vaccines have mainly considered deployment via the World Health Organization's Expanded Programme on Immunization (EPI) in areas of stable endemic transmission of *Plasmodium falciparum*, and have been calibrated for such settings. Their applicability to low transmission settings is unclear. Evaluations of the efficiency of different deployment strategies in diverse settings should consider uncertainties in model structure.

**Methods and Findings:**

An ensemble of 14 individual-based stochastic simulation models of *P. falciparum* dynamics, with differing assumptions about immune decay, transmission heterogeneity, and treatment access, was constructed. After fitting to an extensive library of field data, each model was used to predict the likely health benefits of RTS,S deployment, via EPI (with or without catch-up vaccinations), supplementary vaccination of school-age children, or mass vaccination every 5 y. Settings with seasonally varying transmission, with overall pre-intervention entomological inoculation rates (EIRs) of two, 11, and 20 infectious bites per person per annum, were considered. Predicted benefits of EPI vaccination programs over the simulated 14-y time horizon were dependent on duration of protection. Nevertheless, EPI strategies (with an initial catch-up phase) averted the most deaths per dose at the higher EIRs, although model uncertainty increased with EIR. At two infectious bites per person per annum, mass vaccination strategies substantially reduced transmission, leading to much greater health effects per dose, even at modest coverage.

**Conclusions:**

In higher transmission settings, EPI strategies will be most efficient, but vaccination additional to the EPI in targeted low transmission settings, even at modest coverage, might be more efficient than national-level vaccination of infants. The feasibility and economics of mass vaccination, and the circumstances under which vaccination will avert epidemics, remain unclear. The approach of using an ensemble of models provides more secure conclusions than a single-model approach, and suggests greater confidence in predictions of health effects for lower transmission settings than for higher ones.

*Please see later in the article for the Editors' Summary*

## Abstract 2

Using an ensemble modeling approach, Thomas Smith and colleagues find that targeted mass vaccination with a pre-erythrocytic malaria vaccine RTS,S in low-transmission settings might have better health effects than vaccination through national EPI programs.

## Editors' Summary

**Background:**

The World Health Organization estimates that there are over 200 million cases of malaria each year, and that more than three-quarters of a million people (mostly children living in sub-Saharan Africa) die as a result. Several *Plasmodium* parasites cause malaria, the most deadly being *Plasmodium falciparum*. *Plasmodium* parasites, which are transmitted to people through the bites of infected night-flying mosquitoes, cause recurring fever and can cause life-threatening organ damage. Malaria transmission can be prevented by using insecticides to control the mosquitoes that spread the parasite and by sleeping under insecticide-treated bed nets to avoid mosquito bites. Treatment with antimalarial drugs also reduces transmission. Together, these preventative measures have greatly reduced the global burden of malaria over recent years, but a malaria vaccine could be a valuable additional tool against the disease. At present there is no licensed malaria vaccine, but one promising vaccine—RTS,S—is currently undergoing phase III clinical trials (the last stage of testing before licensing) in infants and children in seven African countries.

**Why Was This Study Done?:**

If the RTS,S vaccine fulfills its promise and is licensed, how should it be used to maximize its effect on the global malaria burden? Should it be given through the World Health Organization's Expanded Programme on Immunization (EPI), which aims to provide universal access to immunization against several infectious diseases during the first three months of life, for example, or through mass vaccination campaigns? Individual mathematical models have been used to investigate this type of question, but the predictions made by these models may be inaccurate because malaria immunity is poorly understood, because little is known about the levels of variability (heterogeneity) in host responses to malaria infection and in malaria transmission, and because it is unclear what the structure of models used to predict vaccine efficacy should be. In this study, the researchers use an “ensemble” approach to model the likely public health impact of the RTS,S malaria vaccine. That is, they simultaneously consider the effect of the vaccine in multiple models of *P. falciparum* dynamics. Ensemble modeling is widely used in weather forecasting and has been used to investigate several other infectious diseases.

**What Did the Researchers Do and Find?:**

The researchers constructed an ensemble of 14 individual-based stochastic simulation models of *P. falciparum* dynamics that included different assumptions about immune decay, transmission heterogeneity, and access to treatment. Such models simulate the passage of thousands of hypothetical individuals through different stages of malaria infection; movement between stages occurs stochastically (by chance) at a probability based on field data. Each model was used to predict the health benefits over 14 years of RTS,S deployment through EPI (with and without catch-up vaccination for infants who were not immunized during their first three months of life), through EPI and supplementary vaccination of school children, and through mass vaccination campaigns every five years at malaria transmission levels of 2, 11, and 20 infectious bites per person per annum (low, medium, and high entomological inoculation rates [EIRs], respectively). The predicted benefits of EPI vaccination programs over the 14-year period were modest and similar over a wide range of settings. However, EPI with an initial catch-up phase averted the most deaths per vaccine dose at higher EIRs. At the lowest EIR, mass vaccination strategies substantially reduced transmission, leading to much greater health effects per dose than other strategies, even at modest coverage.

**What Do These Findings Mean?:**

The ensemble approach taken here suggests that targeted mass vaccination with RTS,S in low transmission settings may have greater health benefits than vaccination through national EPI programs. Importantly, this computer-intensive approach, which used computers made available over the internet by volunteers, provides more secure predictions than can be obtained using single models. In addition, it suggests that predictions made about the health effects of RTS,S vaccination for low transmission settings are more likely to be accurate than those made for higher transmission settings. However, this study only reports the first stages of using ensemble modeling to predict the health effects of RTS,S vaccination. Future studies will need to combine the outputs of multiple models with economic analyses to provide a rational basis for the design of vaccine-containing malaria control and elimination programs.

**Additional Information:**

Please access these websites via the online version of this summary at http://dx.doi.org/10.1371/journal.pmed.1001157.

- Information is available from the World Health Organization on malaria and on malaria immunization; the 2010 World Malaria Report provides details on the current global malaria situation; WHO also provides information on its Expanded Programme on Immunization (EPI), and its Global Immunization Vision and Strategy (some information is available in several languages)
- The US Centers for Disease Control and Prevention provide information on malaria (in English and Spanish), including a selection of personal stories about malaria
- Information is available from the Roll Back Malaria Partnership on the global control of malaria and on malaria in Africa
- The latest results from the phase III trial of RTS,S are available on the website of the PATH Malaria Vaccine Initiative, a global program of the international nonprofit organization PATH that aims to accelerate the development of malaria vaccines and ensure their availability and accessibility in the developing world
- Wikipedia has a page on ensemble forecasting (note: Wikipedia is a free online encyclopedia that anyone can edit; available in several languages)
- OpenMalaria is the open source simulator of malaria epidemiology and control used in this study; BOINC is the open source software for volunteer computing and grid computing that was used to run the simulations
- MedlinePlus provides links to additional information on malaria and on immunization (in English and Spanish)

## Introduction

Malaria vaccines have long been awaited by public health planners [1]. Promising results of recent phase II trials [2],[3] and a current large-scale phase III trial of the RTS,S vaccine increase the urgency of understanding the potential benefits of a licensed malaria vaccine [4]. Consequently, there is an urgent need to understand how a malaria vaccine should best be deployed, and what resources should be invested in deployment. To contribute to addressing these questions, we previously developed a stochastic simulation model of malaria epidemiology and vaccination [5], and used this to make predictions of the likely impact of potential malaria vaccines with a wide range of characteristics, using a limited set of deployment options in African health systems at various transmission levels [6].

This analysis was limited by depending on the assumptions of a single (base) model for malaria transmission dynamics, pathogenesis, and immunity (Table 1). While there is general consensus on the dynamics of malaria in the mosquito, based on the Ross-Macdonald model [7],[8] and discrete time analogues [9],[10], there is considerable uncertainty about the dynamics of malaria immunity, and there is no consensus on what constitutes an adequate mathematical model for immunity. The predictions of vaccination models might be expected to be highly sensitive to assumptions about the dynamics of the natural immune response to the parasite. Levels of heterogeneity both in the host response to infection and in transmission are generally unknown, and this also contributes to uncertainty in model predictions.

**Table 1:** Main assumptions of the base model.

Category	Assumption
**Main assumptions about malaria epidemiology**	The parasite densities experienced in cases of induced malaria in neurosyphilis patients were representative of the natural history of single malaria infections in the naive host.
	All hosts have the same age-dependent risks of infectious bites and co-morbidities, and the same probabilities of treatment for uncomplicated and severe episodes.
	The relationships between asexual parasite densities and infection of mosquitoes seen in induced malaria can be applied across all settings independently of immune status of the population.
	The definition of clinical episodes used in the studies in Dielmo and Ndiop, Senegal [58], corresponds to clinically meaningful events.
	Decay of immunity in the absence of exposure can be neglected as a factor determining clinical outcomes in stable endemic settings.
	The temporal pattern of exposure makes little difference to the resulting immune status (e.g., a 5-y-old who has been infected three times has the same immune status as an adult who has been infected three times).
**Approximations made in applying the same model across different settings**	Patterns of occurrence of clinical episodes in stable endemic settings can be used to make valid inferences about the incidence of clinical disease at intermediate transmission and lower intensities.
	The incidence and age pattern of other diseases that interact with *P. falciparum* in determining its severity are similar across different settings where malaria is endemic.
	Variations between human populations in both acquisition of immunity and response to infection are not important in determining the outcome of infection.
	Differences in the age structure of different populations are of only secondary importance in influencing the impact of partially protective vaccines.
	Differences in patterns of seasonality of malaria transmission do not have a large effect on the public health impact of a vaccine.
	The seasonal pattern of the vectorial capacity for malaria will remain unchanged for the time horizon under consideration, i.e., existing levels of vector control will be maintained but not improved.
**Assumptions about case management**	The model originally intended to represent case management in rural Tanzania [35] represents an adequate approximation of case management systems in other malaria endemic settings.
	The health system will remain essentially unchanged throughout the follow-up period in terms of efficacy of treatment as well as costs.
**Assumptions about vaccine deployment**	The same deployment strategy is assumed to be applied across the whole of the country/region, with uniform levels of access to both vaccination and health care based on data from rural Tanzania.
**Assumptions of the model of vaccine action**	Efficacy of vaccination is independent of host immune status.
	Efficacy of vaccination is unchanged by breakthrough infections.
	Efficacy decays over time following an exponential decay.

Large reductions in malaria transmission have been observed across Africa in recent years [11], so settings with very low transmission are increasingly important. However, predictions of the public health impact of vaccination for such settings have been particularly uncertain. Vaccine trials are carried out in areas with high incidence of malaria, and our previous model was calibrated mainly with data from areas of stable endemic transmission, because this calibration requires seasonal patterns of the entomological inoculation rate (EIR), a quantity rarely available from low transmission sites [12]. Micro-heterogeneity in transmission, in the biological response of the host to infection, and in the health system are well known [13]–[15] and are likely to be extreme in cities [16],[17] or zones with poorly transmitting vectors [18],[19]. At low levels of exposure, natural immunity may decay. It is unclear whether the structures of existing models, including our own [5], are adequate for predicting vaccine effects outside the range of transmission settings used for calibration. The likely impact of the RTS,S vaccine on disease burden in low and unstable transmission settings has therefore been highly uncertain.

Model uncertainty exists because we are unsure of the best model structure. One strategy to address this is to simultaneously consider many different models (known as ensemble modeling). Each element in such an ensemble is based on a distinct set of assumptions, broadly consistent with known biology and field data, leading to a different simulation of the processes and making it possible to evaluate the sensitivity of the predictions to these assumptions. In many disciplines, notably meteorology [20], this is a well-established approach. In infectious disease modeling, such uncertainty analysis is used less frequently, though there have been valuable developments, such as the comprehensive assessment of data-driven uncertainty in the predictions of the SPECTRUM HIV model [21], in recent analyses of models of sexually transmitted infections [22], and in comparisons of different influenza models by the MIDAS network [23]. Formal analysis of model uncertainty using Bayesian melding has also been used, most notably in the predictions from the Joint United Nations Programme on HIV/AIDS Estimation and Projection Package [24], but analysis of malaria models has generally been based on point predictions, simple sensitivity analyses of single models, or, at most, comparisons of small numbers of model formulations [25]–[27]. We recently carried out a probabilistic sensitivity analysis to enable us to make predictions allowing for the uncertainty in the parameters of our base model [28], but there is a need to compare outputs from multiple models if more robust inferences are to be made from such modeling [29].

We now compile an ensemble of stochastic simulation models of malaria epidemiology, incorporating different assumptions about decay of immunity and about heterogeneities in exposure and access to treatment. We use this ensemble to analyze the likely impact of such vaccines in settings with moderate to very low levels of transmission intensity. We consider a range of possible deployment strategies of the vaccine to identify which might be most efficient.

## Methods

### Models of Malaria Epidemiology

The base model is a comprehensive, individual-based model of malaria and vaccination in humans that has been previously published in a supplement to the *American Journal of Tropical Medicine and Hygiene* [5],[30]–[34] Briefly, a simulated population of humans is updated at each 5-d time step via components representing new infections, parasite densities, acquired immunity, uncomplicated and severe malaria episodes (including severe malarial anemia), direct and indirect mortality, infectiousness to mosquitoes, case management [35], and vaccination with a pre-erythrocytic vaccine [36]. The simulated malaria infections each have distinct parasite densities that vary by time step, while the level of malaria transmission is assumed to vary seasonally.

The models are constructed in a modular way, with distinct components that represent infection of humans, blood-stage parasite densities, infectiousness of humans to mosquitoes, incidence of morbidity, and mortality. Each of the components aims to capture the relevant biology, while at the same time fitting available data. Simulated immunity acts mainly by controlling parasite densities [30]. In turn, the simulated incidence of clinical malaria is a function of parasite density [32], as are the incidences of severe disease and malaria-related mortality [31]. Natural immunity to infection without vaccination is acquired only after considerable exposure to *Plasmodium falciparum* malaria parasites [33].

The ensemble was constructed by varying different modular components of the base model. A total of 30 models, each constructed by substituting different versions of one or more components, were investigated. Sixteen of these models were excluded from the ensemble, either because they were very similar to other models in the ensemble, or because the model-fitting algorithm did not find any sets of parameter values that provided an adequate fit to the data (see “Model Fitting” below). Fourteen models were retained. The modifications of the base model that resulted in inclusion of these 14 models are summarized in Table 2 and described in detail in Text S1. Each of these models was assigned the identifier used for the fitting process. Each specific parameterization evaluated in the fitting process (several thousands for each model; see Text S1) was also assigned a unique identifier. The models were programmed in C++ as part of the open source software platform OpenMalaria (http://code.google.com/p/openmalaria/).

**Table 2:** Models/parameterizations included in the ensemble and their behavior.

Model Identifier	Parameterization	Plot of Model Fit	Description	Half-Life of Decay (Years)		Loss Function	Incidence of Clinical Episodes^a^		Incidence of Mortality^b^		Proportion of Episodes Averted^c^	
				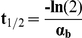	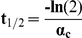		Mean	s.d.	Mean	s.d.	Mean	s.d.
R0001	42327	[5]	Base model	∞	∞	0.82	1.24	0.005	313	32	0.18	0.005
R0063	253417	Text S2	Mass action: 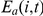 varies mainly between hosts	∞	∞	1.16	1.27	0.006	408	22	0.07	0.003
R0065	242494	Text S3	Mass action: 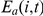 varies between and within hosts	∞	∞	1.14	1.14	0.011	315	37	0.06	0.005
R0068	251951	Text S4	Mass action: 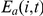 varies mainly within hosts	∞	∞	1.09	1.06	0.005	234	24	0.12	0.004
R0111	245999	Text S5	Fixed decay in effective cumulative exposure	1,000^d^	∞	0.83	1.36	0.009	329	31	0.17	0.001
R0115	253164	Text S6	Fixed decay in effective cumulative exposure	10^d^	∞	1.11	1.59	0.006	242	45	0.11	0.002
R0121	253695	Text S7	Fixed decay in immune proxies	∞	1,000^d^	0.81	1.36	0.008	474	25	0.15	0.003
R0125	247027	Text S8	Fixed decay in immune proxies	∞	10^d^	0.90	1.62	0.013	456	89	0.12	0.003
R0131	250302	Text S9	Estimation of decay in effective cumulative exposure	1,187	∞	0.82	1.37	0.006	296	40	0.15	0.002
R0132	248942	Text S10	Estimation of decay in immune proxies	∞	14	0.82	1.65	0.005	358	34	0.13	0.003
R0133	251169	Text S11	Estimation of both decay parameters	250	19	0.86	1.50	0.009	339	32	0.14	0.004
R0670	243515	Text S12	Heterogeneity in susceptibility to co-morbidity	∞	∞	0.84	1.54	0.010	318	29	0.17	0.002
R0674	252127	Text S13	Uncorrelated heterogeneities in access to treatment and susceptibility to co-morbidity	∞	∞	1.02	2.14	0.001	314	32	0.20	0.002
R0678	252458	Text S14	Heterogeneity in access to treatment	∞	∞	1.15	1.99	0.007	442	28	0.20	0.004

a Incidence of clinical episodes (episodes per person-year) in the absence of intervention at an EIR of 20 ibpa.

b Incidence of mortality (malaria-related deaths per 100,000 person-years) in the absence of intervention at an EIR of 20 ibpa.

c Proportion of clinical episodes that are averted during the first 10 y of a mass vaccination program.

d These parameters were fixed; in other models the decay parameters were estimated. Decays shorter than the shortest fixed values gave unacceptable fits to the data.

s.d., standard deviation.

### Model Fitting

The parameters listed in Table 1 were estimated by fitting to the same set of 61 datasets originally used for fitting the base model. These datasets cover a total of ten different epidemiological quantities (objectives) (see Text S1, and Table 1 in [37]). A genetic algorithm was used to maximize a goodness of fit statistic computed as the weighted sum of the log-likelihood contributions for each objective [37] (see Text S1). To obtain the substantial computing resources required, we used computers made available over the internet by volunteers, via the BOINC (Berkeley Open Infrastructure for Network Computing) volunteer computing software (www.malariacontrol.net).

### Characteristics of the Simulated Settings

The settings for the simulated vaccination programs were assumed to have the seasonal pattern of Namawala, Tanzania [38] scaled to give an overall pre-intervention EIR of 20, 11, or two infectious bites per person per annum (ibpa). The highest EIR of 20 ibpa is similar to standard scenarios that we previously simulated [6],[38], while the EIR of 2 ibpa corresponds to a low transmission setting in which interruption of transmission might be a realistic objective. An EIR of 11 ibpa is close to the transmission intensity at which the base model predicts optimal cost-effectiveness for vaccination of infants via the World Health Organization's Expanded Programme on Immunization (EPI) [28].

The simulated human populations comprised 100,000 people with an age distribution that was approximately stable over time based on data from Ifakara, Tanzania, and with a health system using artemisinin combination therapy with low rates of access [6]. Each simulation begins by exposing the simulated population to the same annually recurring pattern of inoculations for a period of at least 90 y before the intervention program to ensure that the vaccination program starts with infection status and immune status at steady state values over the whole age range.

### Simulation of Vaccine Effects

Pre-erythrocytic vaccination was simulated as described previously [6],[38], assuming that vaccination leads to a reduction in the proportion of inoculations from the bites of infected mosquitoes that result in blood-stage infection, and that the underlying vaccine efficacy is equal to the proportion by which this force of infection is reduced. This value is higher than the efficacy in preventing clinical malaria [36].

Based on analyses of the initial phase II trials of RTS,S [36], which used the AS02 adjuvant, we estimated that this underlying efficacy should take a value of 52% [36]. More recent trials of RTS,S/AS01 have demonstrated somewhat higher efficacy, so for the simulations presented here, we assumed an overall average underlying efficacy of 60% immediately after the third dose.

The rate of decay over time in the immunity induced by the vaccine, and hence in its underlying efficacy, is an important driver of the overall uncertainty in projections of long-term effectiveness of RTS,S [28], largely because of difficulties in measuring it. Extended follow-up of field trials of malaria vaccines provide direct evidence on decay of efficacy over time, but variations in efficacy observed in the field relate only indirectly to the underlying biological effect of the vaccine because of heterogeneity in the host population. In particular, transmission heterogeneity and acquired immunity bias downwards the estimates of efficacy [36],[39]. These heterogeneities also result in substantial biases in estimates of rates of decay of clinical protection when only the first clinical episode is analyzed for each trial participant, as in the main analyses of RTS,S trials [40],[41]. However, even when appropriate statistical methods are used to analyze all malaria episodes occurring in the trial cohort, measured clinical efficacy is likely to decay because the age profile of morbidity and mortality in vaccine recipients shifts towards the pattern found in lower transmission settings. This shift of morbidity to older ages [42],[43] will appear as a decay in efficacy over time in a controlled trial, thus complicating inference of the decay rate of the underlying vaccine effect from trial data.

An alternative approach to estimate decay in underlying efficacy is to measure immune effectors. This has the advantage that data can be obtained even from individuals who do not get sick, but there is uncertainty in whether the relevant immune response has been assessed, and how it maps onto protection [44]. The relationship between RTS,S-induced protection and anti-circumsporozoite antibody levels is not consistent over time or across trials [40],[45]. In the longest reported follow-up of an RTS,S trial to date [41], the effects on prevalence and antibody levels were sustained over 4 y [45], while clinical efficacy appeared to decline [41].

Overall, therefore, these field studies provide little data from which to estimate the underlying rates of decay of protection, with the best evidence being the sustained effect on prevalence reported in Mozambique [41]. In order to better understand how to translate trial results into projections of effectiveness, we simulated a range of field trials with different decays of underlying efficacy, and considered how this would translate into trial results and effectiveness.

For each of the models, we simulated field trials with an underlying efficacy (immediately on completion of the vaccination schedule) of 60%. This quantity is equivalent to the assumed efficacy in preventing infection in challenge trials in naive volunteers. The simulated trials all used the initial 20-ibpa transmission setting and the EPI schedule, conducted in total populations of 20,000 people, with 50% of newborn children (assigned randomly) receiving the full course of vaccine. Three sets of trials were simulated, with different exponential decays over time in the underlying efficacy (5-y half-life, 10-y half-life, and negligible decay).

The effect of incomplete courses of vaccination is also highly uncertain. For the main analyses we assigned an average initial underlying efficacy of 40% after a single dose, and 50% after two doses. Booster (fourth or subsequent doses) of vaccine return the simulated efficacy to the value achieved immediately after the third dose. To explore the sensitivity of the results to this assumption we carried out an additional series of simulations in which incomplete courses of vaccination were assumed to have zero efficacy.

### Vaccine Deployment Modalities

Six modalities for deployment of vaccines in programs were simulated.

#### Expanded Program on Immunization

Delivery of the vaccines through the EPI with vaccinations is at ages 1, 2, and 3 mo. We assume coverage of full vaccination (three doses) corresponds to that reported in Tanzania for three doses of diphtheria–tetanus–acellular pertussis–hepatitis B virus vaccine in the year 2003, which stood at 89%. The assumed dropout rate from the first to the third dose is 6%, since coverage for the first dose of diphtheria–tetanus–acellular pertussis–hepatitis B virus vaccine was 95%.

#### Expanded Program on Immunization with catch-up

Vaccination is at ages 1, 2, and 3 mo, but in addition, the program includes catch-up vaccination of children under 18 mo of age, with each dose reaching 80% of eligible children.

#### Expanded Program on Immunization with vaccination of school children

Vaccination is at ages 1, 2, and 3 mo, but with additional vaccination of primary school children (aged 6 to 11 y). The program starts with each dose reaching 80% of school-age children (simulating vaccination of entire schools). Subsequent annual intakes of new enrollments are vaccinated. Once children who have received EPI vaccination start to enter school, the annual school vaccination rounds deliver only a single (booster) dose of vaccine.

#### Expanded Program on Immunization with vaccination of school children at low coverage

This modality is the same as the previous one, but with only 50% of children reached at each vaccination round.

#### Mass vaccination

A mass vaccination campaign is carried out at the beginning of the intervention period, comprising three monthly rounds to deliver a full course of vaccination, and additional campaigns every 5 y subsequently. The simulated coverage level was 80% at each round of vaccination in each age group.

#### Mass vaccination with low coverage

This modality is the same the previous one, but with only 50% of the population reached at each vaccination round.

**Figure 1:**
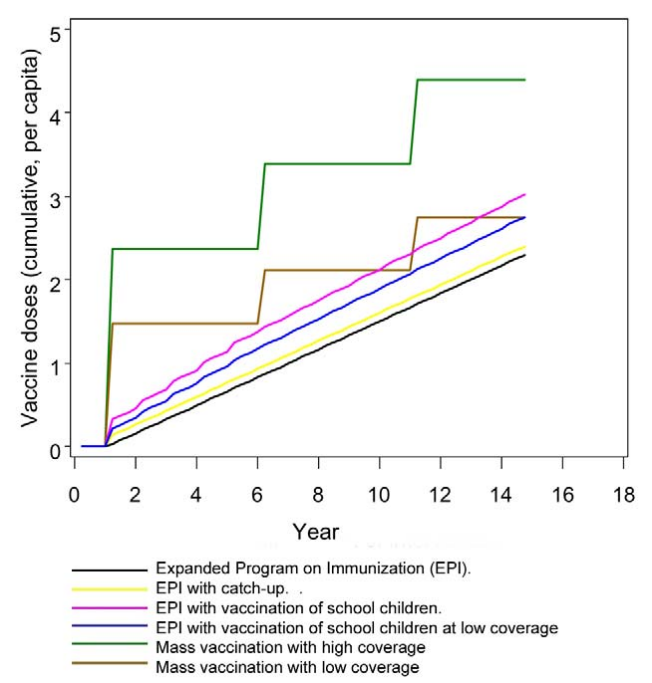
Numbers of doses of vaccine delivered by various deployment strategies over time.

EPI and school vaccination result in vaccination at almost constant rates, with established programs aiming to administer three and four doses for each child, respectively (Figure 1). Mass vaccination strategies deliver vaccines intermittently, leading to a stepwise increase in the cumulative numbers of doses delivered (Figure 1).

### Predictions and Analysis

The results were plotted for time horizons up to 14 y. For each simulation, the prevalence of patent infection in the population (assuming diagnosis by standard microscopy procedures), incidence of clinical malaria, severe malaria, and overall malaria-related mortality (summing direct and indirect deaths) were monitored. The corresponding effectiveness values, defined as the proportion of events averted, were also computed. To provide information on stochastic variation as well as variation between models in the ensemble, each scenario was simulated five times for each of the 14 models, with different streams of random numbers for each of the five simulations.

Graphical output was generated using the SAS GPLOT procedure (SAS Institute, version 9.2 for Windows).

## Results

### Model Fitting and Parameter Estimates

The 14 models (Table 2) that satisfied the criteria for inclusion adequately reproduced the age-specific patterns of infection and morbidity to which they were fitted (detailed plots of fit available from the authors).

Some of the estimated parameter values varied considerably between the 14 members of the ensemble (Table 3). In particular, several fits gave much higher estimates of the critical value of the number of entomological inoculations in the model of pre-erythrocytic immunity, 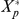, than in the base model. High values of this parameter correspond to a minimal role of the pre-erythrocytic component in naturally acquired immunity. The re-estimated parameters relating to severe morbidity and mortality retained values very similar to those in the base model, with the exception that values of 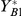 were all lower, corresponding to a lower parasitemia threshold for severe malaria. These lower values seem more reasonable than those in the base model when compared with actual measured parasite densities in severe malaria cases.

**Table 3:** Re-estimated values of parameters common to different models.

Parameter		Units/Dimension	Model/Parameterization														
Symbol	Description		Base	Overdispersion in Force of Infection			Decay in Immunity							Additional Heterogeneity			
			R0001	R0063	R0065	R0068	R0111	R0115	R0121	R0125	R0131	R0132	R0133	R0670	R0673	R0674	R0678
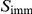	Lower limit of success probability of inoculations in immune individuals	Proportion	0.14	0.24	0.19	0.10	0.11	0.12	0.15	0.15	0.17	0.16	0.19	0.14	0.22	0.16	0.15
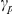	Steepness of relationship between success of inoculation and 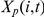	Dimensionless	2.04	1.66	1.78	2.04	1.96	1.96	2.11	1.80	2.91	2.12	1.77	1.90	1.76	2.82	2.28
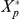	Critical value of cumulative number of entomological inoculations	Inoculations	1,514.4	7,771.4	1,011.4	633.4	4,694.1	8,057.4	2,920.7	42,848.1	4,899.7	2,963.8	5,732.7	103,655.7	2,411.0	3,529.1	63,562.9
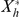	Critical value of cumulative number of infections	Infections	97.3	258.7	182.0	299.0	84.9	57.1	71.7	57.7	68.0	51.7	62.2	80.8	96.5	100.1	99.8
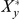	Critical value of cumulative number of parasite days	Parasite days/µl×10^−7^	3.52	0.21	2.41	0.55	4.74	5.63	6.86	8.93	12.06	10.27	40.31	7.38	2.61	6.33	4.48
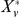	Critical value of cumulative number of infections for variance in parasite densities	Infections	0.92	0.89	0.91	0.90	0.91	0.91	0.92	0.91	0.92	0.91	0.91	0.92	0.93	0.92	0.93
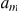	Maternal protection at birth	Dimensionless	0.90	0.93	0.95	0.91	0.91	0.92	0.91	0.89	0.90	0.88	0.92	0.91	0.91	0.89	0.93
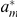	Decay of maternal protection	Per year	2.53	3.15	2.24	2.51	2.64	3.11	2.72	2.27	2.47	2.47	3.33	2.33	3.25	2.52	2.77
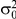	Fixed variance component for densities	[ln(parasites/µl)]^2^	0.66	0.64	0.63	0.67	0.67	0.68	0.68	0.71	0.68	0.72	0.67	0.66	0.58	0.61	0.67
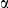	Factor determining increase in 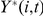	Parasites^2^µl^−2^day^−1^	142,602	439,384	159,642	189,968	102,833	175,521	138,186	190,399	129,867	104,690	99,055	120,493	114,403	82,441	72,126
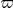	Decay rate of pyrogenic threshold	Year^−1^	2.52	2.25	2.91	2.59	2.42	2.14	2.61	2.27	2.31	2.38	2.84	2.34	2.71	2.46	2.52
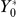	Pyrogenic threshold at birth	Parasites/µl	296.3	1169.4	324.2	219.5	194.8	740.9	576.4	349.4	274.7	268.2	672.4	215.5	477.7	263.0	680.9
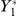	Critical value of parasite density in determining increase in 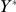	Parasites/µl	0.60	0.72	0.62	0.58	0.64	0.55	0.62	0.61	0.59	0.65	0.61	0.60	0.60	0.58	0.54
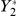	Critical value of 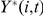 in determining increase in 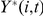	Parasites/µl	6,502.3	60,269.3	8,035.9	7,849.9	6,635.7	8,583.7	10,239.5	14,757.1	11,311.3	10,485.7	5,154.5	11,434.2	8,557.5	12,966.6	9,809.1
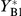	Parasitemia threshold for severe episodes type B_1_	Parasites/µl	784,000	220,000	536,000	518,000	264,000	252,000	330,000	324,000	377,000	274,000	292,000	340,000	283,000	429,000	446,000
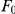	Prevalence of co-morbidity/susceptibility at birth relevant to severe episodes (B_2_)	Proportion	0.092	0.122	0.106	0.097	0.070	0.087	0.074	0.114	0.070	0.056	0.069	0.088	0.067	0.104	0.083
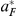	Critical age for co-morbidity	Years	0.117	0.073	0.127	0.097	0.098	0.113	0.124	0.089	0.119	0.128	0.099	0.130	0.111	0.065	0.110
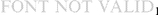	Case fatality for severe episodes in the community compared to in hospital	Odds ratio	2.09	2.11	2.09	1.99	2.10	2.04	2.10	2.22	2.17	2.07	2.09	2.05	2.09	2.17	2.10
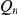	Non-malaria intercept for infant mortality rate	Deaths/1,000 live births	49.5	50.3	55.7	59.2	49.1	63.8	45.1	44.2	54.7	43.8	47.7	56.2	48.5	64.3	56.8
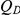	Co-morbidity intercept relevant to indirect mortality	Proportion	0.019	0.030	0.014	0.012	0.016	0.008	0.021	0.030	0.010	0.014	0.018	0.010	0.014	0.012	0.015

The fitting process is comprehensively described in the Text S1.

### Simulations of Field Trials

**Figure 2:**
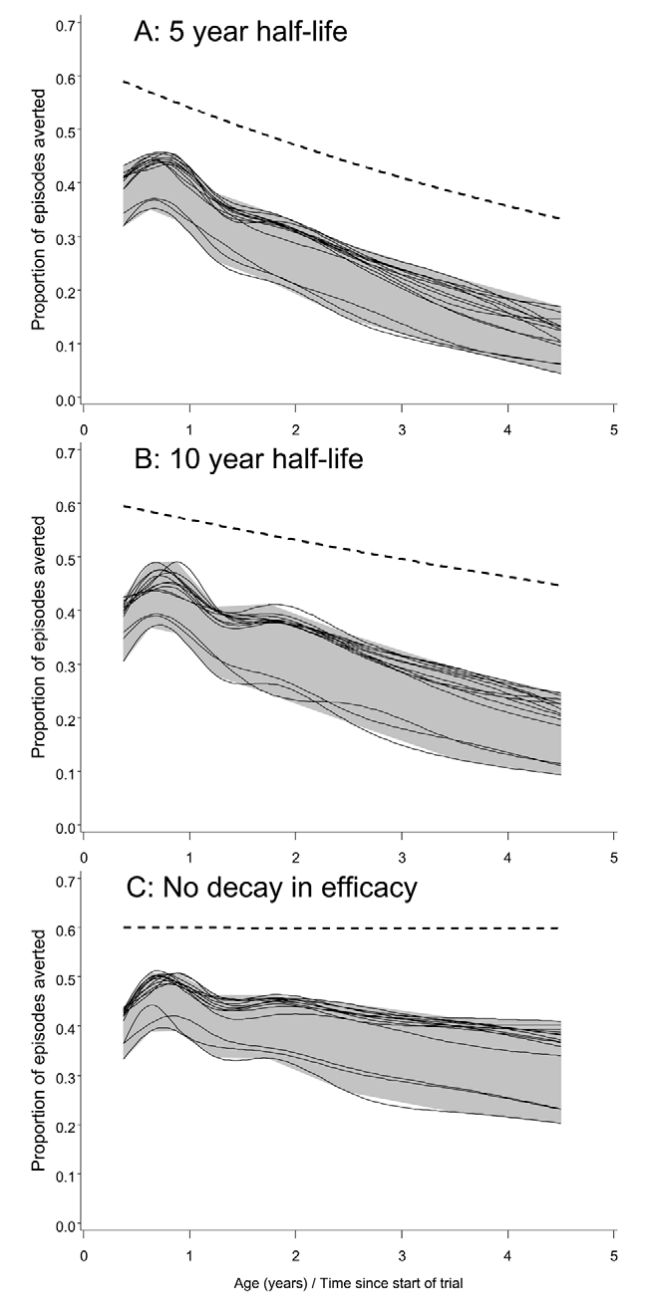
Efficacy in averting clinical episodes in simulated clinical trials. The three panels correspond to different decay rates of vaccine efficacy: (A) 5-y half-life, (B) 10-y half-life, and (C) no decay. The dashed lines give the underlying efficacy. Each continuous line corresponds to a different model within the ensemble, and displays spline-smoothed estimates of efficacy in averting clinical disease. The grey area is an envelope enclosing all the simulation results. All simulations refer to the 20-ibpa transmission setting and the EPI schedule described in the Methods.

In the analyses of the simulated field trials, clinical efficacy was computed as the proportion of all clinical episodes averted for each 3-mo period of follow-up. Analyses of clinical efficacy for 4.5 y of follow-up for each child (Figure 2) indicated that in all models the clinical efficacy in the simulated trials was considerably less than the underlying efficacy. These modeled efficacies were higher than those observed in the original field trial of RTS,S/AS02 in children [46], but lower than that reported in the most recent trials of RTS,S/AS01 [47]. In these simulations the largest differences between the underlying efficacy and the efficacy in the trial simulation were with models R0063, R0065, and R0068, which assumed higher levels of transmission heterogeneity than did the other elements of the ensemble.

In each simulation, the clinical efficacy showed an initial increase during the latter part of the first year of life, and then a decline (Figure 2). This pattern mirrors the age pattern in simulated blood-stage immunity [30], which includes a component of maternal immunity that declines with age, and acquired components that cumulate in response to exposure. In the simulated trials, the declines in clinical efficacy with age after the first birthday were much steeper than the declines in the underlying efficacy, implying that field estimates of decay of immunity would considerably overestimate the rate at which efficacy was being lost.

Field efficacy decays more rapidly than the underlying vaccine effect because vaccinees experience an age pattern of clinical disease equivalent to that of a population with reduced exposure. This corresponds to an age shift in the peak of mortality, which can be seen in the field data used to parameterize the models [48]. The shift of disease to older age groups is manifested in the simulated trials (and presumably in actual field trials) as decay with age (and hence time) in the measured efficacy. This decay is evident even in the absence of a decay in the underlying efficacy (Figure 2C), though the presence of decay in the underlying efficacy considerably increases the decay rate that would be observed.

We would expect the same biases in estimation of decay rates to occur in actual field trials that continue long enough and have sufficient power.

### Simulated Levels of Transmission

**Figure 3:**
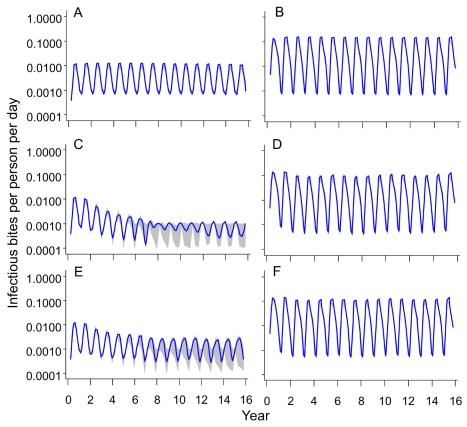
Simulated Entomological Inoculation Rates over time.

Figures 3A and 3B show the assumed seasonally recurring patterns of transmission used as input for the simulations for 2 and 20 ibpa, respectively. The 11-ibpa simulated pattern is given in Figure S1.

The effects of a vaccination program on transmission are illustrated by trends in the simulated EIR after the start of the simulations (Figures 3C–3F and S1). Delivery of vaccine via EPI or by vaccination in schools had minimal effect on these trends, and is therefore illustrated only in Text S1.

With an initial EIR of 2 ibpa, mass vaccination with high coverage reduced transmission by an order of magnitude, but in most simulations, a new steady state was reached (Figure 3C) after the second round of vaccination (at year 6; see Figure 1). Mass vaccination at low coverage (Figure 3E) had similar effects.

Despite the low levels of simulated EIR achieved in these scenarios, interruption of transmission (assessed as the absence of patent infection from the human population) did not occur in any of the simulations. Mass vaccination with low coverage resulted in only a modest reduction in EIR, and the maximal effect was achieved about 9 y into the simulation (Figure 3E).

With an initial EIR of 20 ibpa, even mass vaccination had little effect on the EIR, with very close agreement among the 70 simulations, (corresponding to five different seed values for each of the 14 models) (Figure 3D and 3E). With other vaccination deployment modalities, effects on EIR were almost imperceptible (Figure S1). Results of the 11-ibpa scenarios were very similar to those of the 20-ibpa scenarios.

### Simulated Prevalence

**Figure 4:**
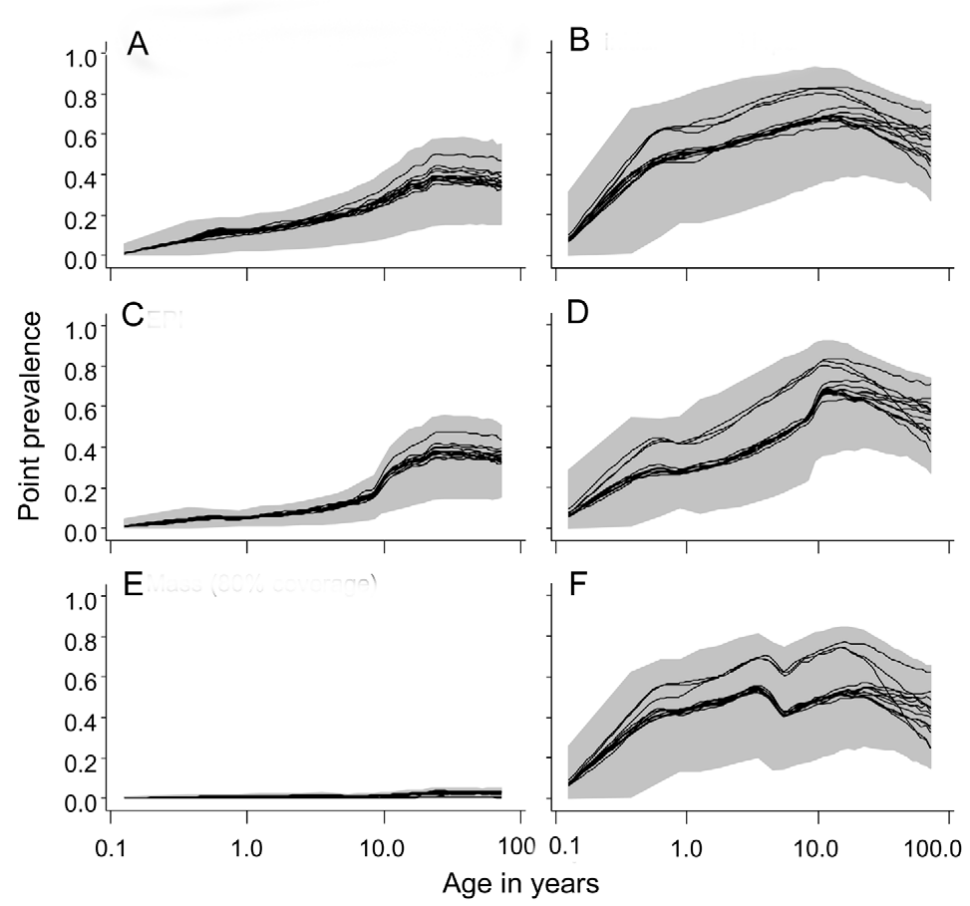
Age prevalence curves during the tenth year of follow-up. (A) EIR = 2 ibpa, no intervention. (B) EIR = 20 ibpa, no intervention. (C) EIR = 2 ibpa, EPI vaccination. (D) EIR = 20 ibpa, EPI vaccination. (E) EIR = 2 ibpa, mass vaccination, high coverage. (F) EIR = 20 ibpa, mass vaccination, high coverage. The lines correspond to the median values of the five simulations for each model within the ensemble of the prevalence, computed from values averaged within each simulation over the full year; the grey areas are the envelopes delimited by the 2.5 and 97.5 percentiles of the simulations.

In the absence of interventions, the simulated prevalence of malaria at 2 ibpa was both much lower and had the peak shifted to older ages compared to at 11 or 20 ibpa (Figure 4). There was less variation between simulations at 2 ibpa (Figure 4A) than at 20 ibpa (Figure 4B). The patterns for 11 ibpa were intermediate (Figure S2).

EPI vaccination had little effect on overall prevalence, but some reduction in the youngest age groups is evident at both transmission intensities (Figure 4C and 4D). Mass vaccination almost completely eliminated prevalent infections at 2 ibpa (Figure 4E), but, as found in the previous analyses [6],[28], had rather little effect on prevalence at the higher transmission intensity (Figure 4F). The effects for an initial EIR of 11 ibpa were similar to those for 20 ibpa (Figure S2). Catch-up or school vaccination had little effect on prevalence additional to that of EPI vaccine delivery.

### Simulated Incidence of Uncomplicated Malaria Episodes

**Figure 5:**
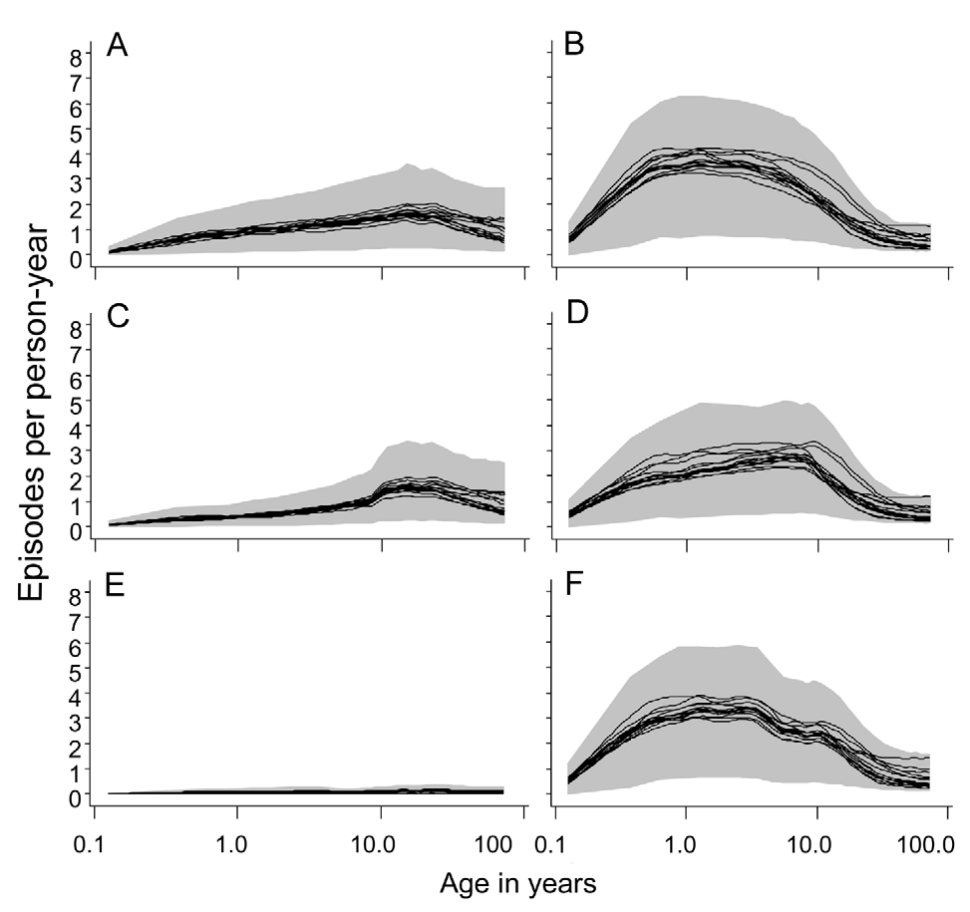
Age incidence curves during the tenth year of follow-up. (A) EIR = 2 ibpa, no intervention. (B) EIR = 20 ibpa, no intervention. (C) EIR = 2 ibpa, EPI vaccination. (D) EIR = 20 ibpa, EPI vaccination. (E) EIR = 2 ibpa, mass vaccination, high coverage. (F) EIR = 20 ibpa, mass vaccination, high coverage. The lines correspond to the median values of the five simulations for each model within the ensemble of the incidence of clinical malaria, computed from values averaged within each simulation over the full year; the grey areas are the envelopes delimited by the 2.5 and 97.5 percentiles of the simulations.

In the absence of a vaccination program, the overall incidence rates are similar for the different transmission intensities but with a substantial shift to older age groups in lower transmission settings (Figures 5 and S3), as is observed in the field [48]. There was less variation among simulations in the incidence at low transmission (Figures 5 and S3). This variation was mostly between the different models (Table 2), with the variation between seeds (the standard deviation columns in Table 2) very low compared to the mean incidence rates. The highest incidence of clinical episodes was associated with the models R0674 and R0678, in which additional heterogeneities were simulated.

**Figure 6:**
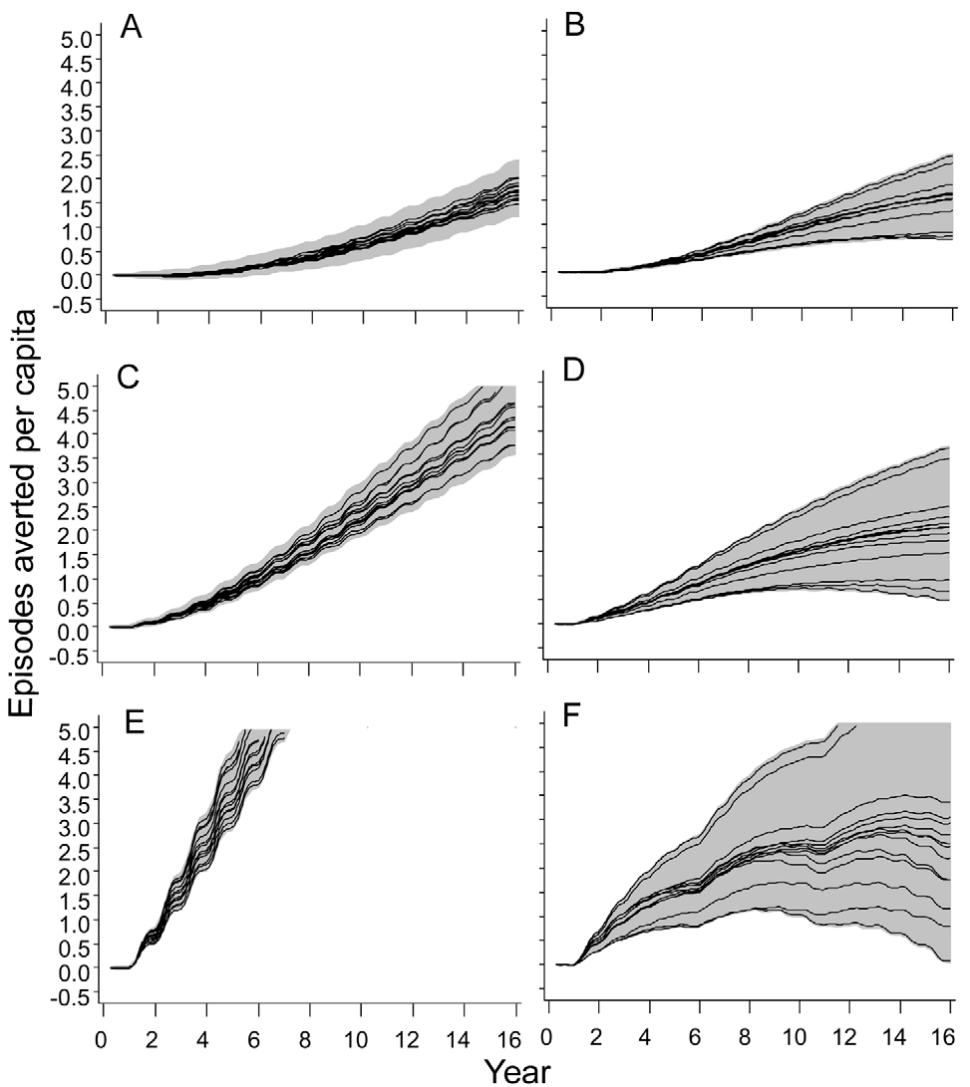
Numbers of clinical episodes averted. (A) EIR = 2 ibpa, EPI vaccination. (B) EIR = 20 ibpa, EPI vaccination. (C) EIR = 2 ibpa, EPI and school vaccination, high coverage. (D) EIR = 20 ibpa, EPI and school vaccination, high coverage. (E) EIR = 2 ibpa, mass vaccination, high coverage. (F) EIR = 20 ibpa, mass vaccination, high coverage. The lines correspond to the median values of the five simulations for each model within the ensemble of the episodes averted per capita, computed from values averaged within each simulation over the full year; the grey areas are the envelopes delimited by the 2.5 and 97.5 percentiles of the simulations.

Irrespective of the transmission setting, EPI vaccination averted only a modest number of malaria episodes, with most predictions of the number of episodes averted falling within a rather narrow envelope (Figure 6A and 6B). Similar patterns were observed for all three transmission intensities and for EPI with catch-up, or for school vaccination (Figure S4). The rather narrow envelopes enclosing the predictions indicate that the vaccination effects are not very sensitive to the various assumptions about immunity or heterogeneity.

Mass vaccination at an initial EIR of 2 ibpa was predicted to avert substantial morbidity, even at the lower coverage level. The trajectory of cumulative numbers of episodes averted over time was close to a straight line, with some influence of seasonality (Figure 5E), implying that the year-to-year benefits remain more or less constant. Variation between models and seeds was low (indicated by the closeness of the maxima and minima) but showed some tendency to increase over time.

In simulations of mass vaccination at initial EIRs of 11 ibpa (not shown) or 20 ibpa, substantial numbers of clinical episodes were averted in the first few years of the simulation (Figure 6F), but the curves of numbers of events averted tended to flatten out, so that the proportion of episodes averted decreased as the time horizon grew longer. Simulations of school vaccination strategies (Figure 6C and 6D) generated results that were intermediate between those for EPI and mass vaccination strategies.

Although the different models and parameterizations all predicted these patterns, the envelopes of the results for mass vaccination at initial EIRs of 11 and 20 ibpa were much wider than those for the lower transmission setting, indicating more model uncertainty, especially in the prediction of the number of clinical episodes averted (Figure S4). This proportion varied considerably between models (Table 2), with the percentage averted, cumulated over the first 10 y of the simulated program, varying from only 6%–7% for the models with between host variation in susceptibility to infection, to 20% in models with heterogeneity in access to treatment. The stochastic variation within models, measured by the standard deviation of these percentages, was small.

### Simulated Incidence of Severe Disease and Mortality

In the absence of a vaccination program, there is a strong decrease with age in simulated incidence of severe events at 20 ibpa, but rather little age dependence at 2 ibpa, with results for 11 ibpa being more similar to those of the higher transmission intensity than to those of the lower one (Figures S5, S6, and 7). The simulations agree that at 2 ibpa, somewhat more of the severe disease and mortality is in adolescents and young adults. Existing field data provide only a weak evidence base on age dependence of severe outcomes in low transmission settings [31]. Most research has emphasized morbidity in younger age groups, but where malaria exposure occurs only infrequently, it is likely to result in severe disease among older hosts. An exposure of 2 ibpa is intermediate between the EIRs to which the models were fitted and is a setting with only sporadic exposure and negligible acquired immunity, where all age groups are vulnerable to disease with a high case-fatality rate if not treated. Stochastic variation is a substantial contributor to variations between simulations in mortality rates because of the smaller numbers of deaths in low transmission settings (Table 2).

**Figure 7:**
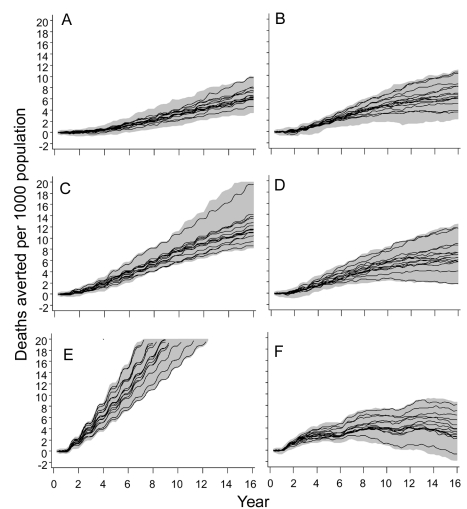
Numbers of malaria-related deaths averted. (A) EIR = 2 ibpa, EPI vaccination. (B) EIR = 20 ibpa, EPI vaccination. (C) EIR = 2 ibpa, EPI and school vaccination, high coverage. (D) EIR = 20 ibpa, EPI and school vaccination, high coverage. (E) EIR = 2 ibpa, mass vaccination, high coverage. (F) EIR = 20 ibpa, mass vaccination, high coverage. The lines correspond to the median values of the five simulations for each model within the ensemble of the deaths averted per 1,000 population, computed from values averaged within each simulation over the full year; the grey areas are the envelopes delimited by the 2.5 and 97.5 percentiles of the simulations.

At all three transmission intensities, simulated vaccination through EPI had only modest effects on the patterns of severe disease or mortality (Figures S7 and S8). In contrast, there were substantial effects of mass vaccination at 2 ibpa on the number of severe episodes averted (Figure S7) and deaths averted (Figures S8 and 7). These effects are similar to those on overall clinical incidence (Figure 6), in that the cumulated number of episodes averted continued to increase more or less linearly with time, even with some upward curvature, corresponding to the decrease in transmission. The proportions of clinical episodes, severe episodes, and deaths averted all increased with the duration of the time horizon, each quantity reaching values of about 80% after 14 y. However, the envelopes enclosing all the predictions (Figures 6 and 7) widened with the duration of the follow-up, indicating that uncertainty increases considerably with the length of the time horizon.

At 20 ibpa, mass vaccination showed similar temporal patterns in its effects on averting severe disease (Figure S7) and death (Figures 7 and S8), as for uncomplicated malaria, but with an even more pronounced tendency for the health benefits to decline with longer time horizons, so that the plots of cumulative proportions of episodes averted show decreases with time. In contrast to the pattern for uncomplicated episodes, the variation between models was less at 20 ibpa than at 2 ibpa.

**Figure 8:**
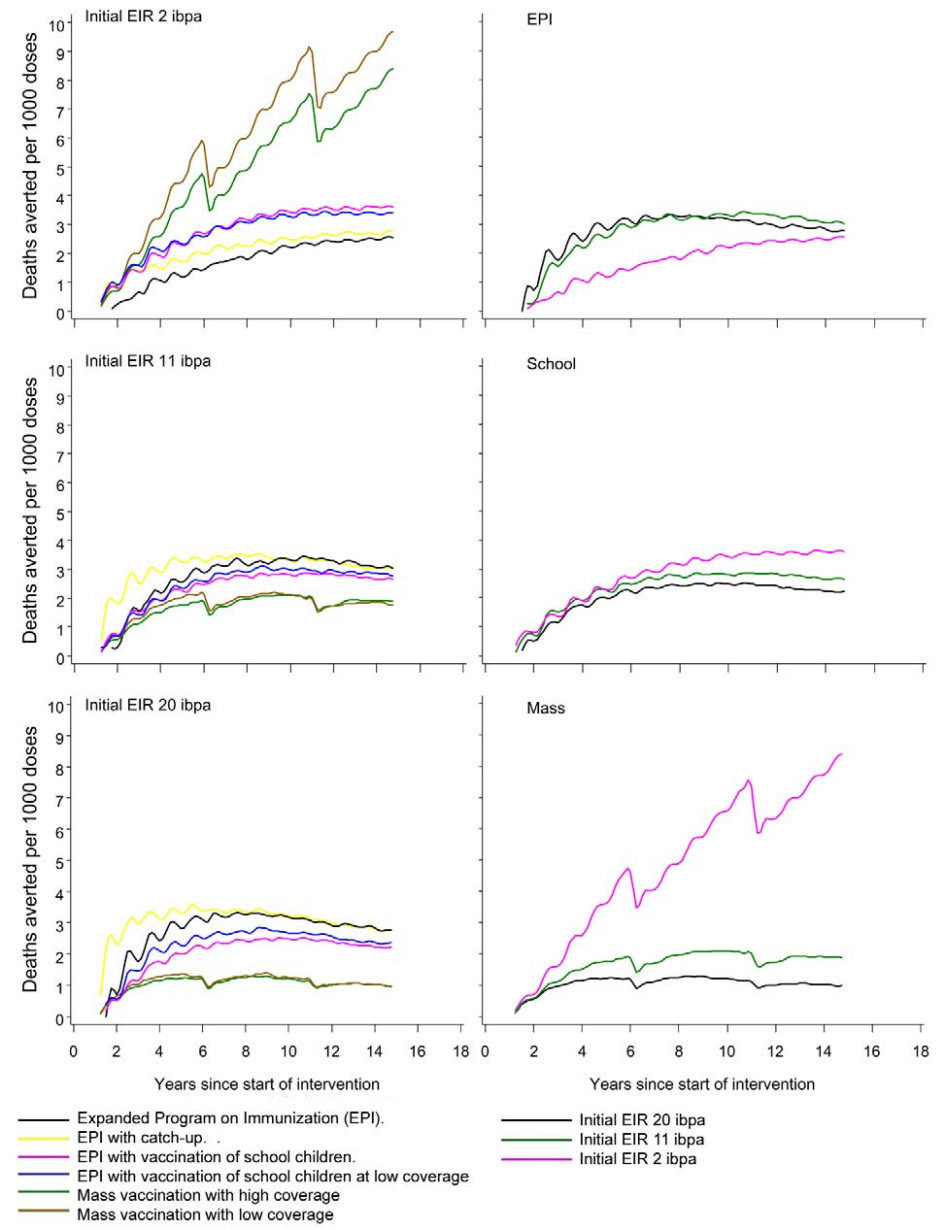
Numbers of malaria-related deaths averted in relation to number of vaccine doses administered. The values plotted are the medians of all simulations. In the left-hand panels, the different lines correspond to different deployment strategies; in the right-hand panels, the different lines correspond to different initial transmission intensities.

Since EPI deploys the fewest doses of vaccine, it is not surprising that it delivers the lowest impact, and the number of severe events or deaths averted per dose of vaccine represents a simple measure of the overall efficiency of a program that adjusts for this. Given the rather limited variation between models in predicted impact, the main patterns are captured by a simple average of this ratio across simulations. Figure 8 compares the numbers of deaths averted per 1,000 vaccine doses by each of the different strategies. At 20 ibpa, EPI performs better on this metric than any of the other strategies, with catch-up providing an initial benefit for the first 6 y of the program. At 11 ibpa, the ranking of strategies is the same, but at 2 ibpa, the greatest benefit per vaccine dose is obtained with a mass vaccination strategy. The same data are also considered from the perspective of comparing the benefit per dose of a given strategy across transmission intensities (Figure 8). EPI provides more benefit (considered over the whole simulation period) at 11 ibpa than at either of the other EIRs (corresponding to previous findings [28]). EPI benefit cumulates only very slowly at 2 ibpa. School vaccination delivers more benefit per dose at 2 ibpa than it does at either other EIR, whereas mass vaccination provides the highest benefit per dose at 2 ibpa, but performs poorly at higher transmission levels.

### Effects of Decay of Protection over Time

The effectiveness of a vaccination program in averting morbidity and mortality is quantified by the overall population proportion of episodes that are averted. The effectiveness calculation includes episodes in individuals who are not vaccinated or are not eligible for vaccination, includes new recruits into the population, and may consider longer time horizons, within which there may be gradual effects of reduced exposure on acquisition of immunity. All these effects modify the relationship between effectiveness and decay in efficacy, defined either as underlying efficacy or as measured in a field trial.

**Figure 9:**
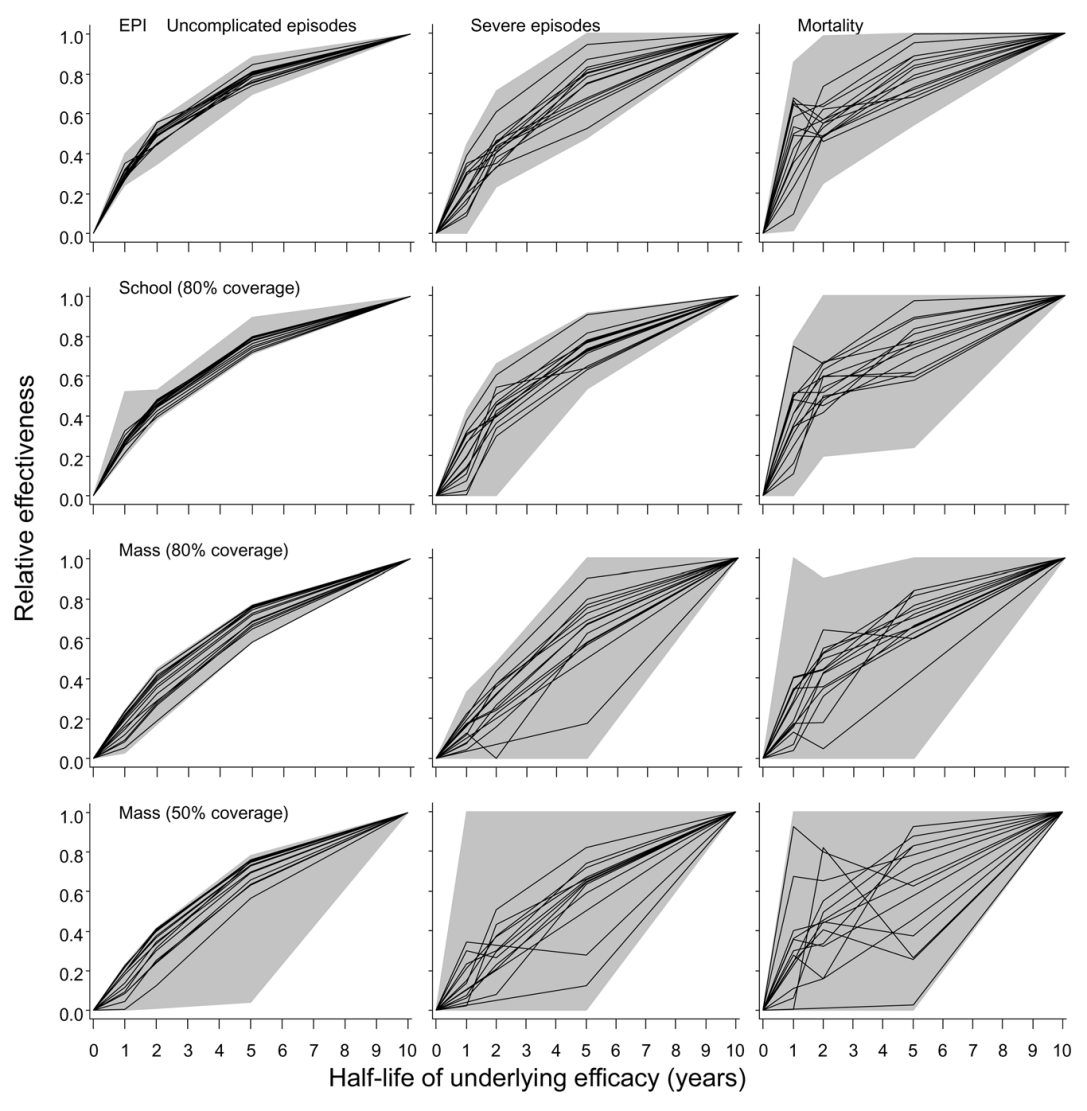
Effect of half-life of underlying efficacy on effectiveness of vaccination. The three columns correspond to distinct outcomes (uncomplicated episodes, severe malaria, and malaria-related mortality [including both direct and indirect]). The rows correspond to different deployment strategies. The horizontal axis in each graph corresponds to the half-life of the underlying effect of the vaccine. The black lines give the median relative effectiveness during the first 10 y of the program for each model, where relative effectiveness is defined as the proportion of events averted divided by the proportion of events averted by a vaccine with a 10-y half-life. The grey areas correspond to the range of this relative effectiveness for all simulations (three simulations for each model and each half-life). All simulations refer to the 20-ibpa (initial) transmission setting.

Previous analyses of the base model considered the effects of decay of immunity on effectiveness of both EPI vaccination [5],[6] and mass vaccination [6]. These analyses indicated that effectiveness is not very strongly influenced by the half-life of efficacy, providing this half-life is of the order of 5–10 y or more. Additional simulations using all the models in the ensemble considered half-lives of 1, 2, or 5 y in the underlying efficacy (Figure 9). Each scenario was simulated with three distinct random number seeds for each of the 14 models, and for a simulated population of 100,000 individuals, exposed initially to an EIR of 20 ibpa. Effectiveness was computed for the first 10 y after introduction of the intervention.

To facilitate recalculation of likely effects of vaccination programs with different assumed half-lives of vaccine efficacy, the results are presented in terms of the effectiveness of the program relative to that of the vaccine with the standard half-life of 10 y assumed in the main simulations. These simulations confirmed the insensitivity of effectiveness to half-life at half-lives of 5–10 y for all 14 models (Figure 9). There was very little difference between the models in the extent of reduction in effectiveness against uncomplicated episodes at a given half-life. This applied irrespective of the deployment strategy of the vaccine (compare rows in Figure 9; catch-up and low coverage school strategies are not shown as these show patterns very similar to those of EPI and high coverage school strategies). An exception was mass vaccination at low coverage, where one simulation with a 10-y half-life vaccine predicted a very high effectiveness, leading to an anomalously low effectiveness in the shorter half-life scenarios with which it was compared.

In comparison to effects for uncomplicated disease, there was more variation between simulations in the extent of reduction in effectiveness against severe disease and mortality. Nevertheless, the overall shapes of the curves for these outcomes in Figure 9 are generally similar to those for uncomplicated disease. Most of the variation between simulations is attributable to stochastic variation, rather than variation between models.

### Sensitivity of Effectiveness Estimates to Efficacy of Incomplete Courses of Vaccination

Additional simulations were carried out to examine the effect of the assumed efficacy of incomplete courses of vaccination (see Methods). These simulations considered the effect of assuming that an incomplete course of either one or two doses of vaccine has zero efficacy. The full course of three doses was assumed, as in the main analyses, to have a 60% underlying efficacy decaying with a 10-y half-life. The results of these simulations are expressed as effectiveness relative to that of the standard scenarios described above, in which the efficacy of one dose was set at 40%, and of two doses at 50% (Table 4). All these simulations assumed a pre-intervention EIR of 20 ibpa.

**Table 4:** Effects of the assumption of zero efficacy of incomplete vaccination courses.

Vaccine Deployment Modality	Median Relative Effectiveness of Vaccination (Minima, Maxima of 42 Simulations)		
	Uncomplicated Episodes	Severe Episodes	Malaria-Related Mortality
EPI	0.93 (0.83, 1.00)	0.95 (0.65, 1.26)	0.89 (0.54, 1.47)
EPI with catch-up	0.88 (0.81, 0.99)	0.92 (0.70, 1.16)	0.88 (0.60, 1.24)
EPI with vaccination of school children	0.77 (0.72, 0.86)	0.91 (0.68, 1.12)	0.91 (0.62, 1.65)
EPI with vaccination of school children at low coverage	0.72 (0.59, 0.90)	0.87 (0.63, 1.33)	0.85 (0.43, 1.50)
Mass vaccination with high coverage	0.64 (0.53, 0.68)	0.57 (0.00, 1.92)	0.58 (0.04, 1.00)
Mass vaccination with low coverage	0.28 (0.01, 0.34)	0.24 (0.00, 1.57)	0.28 (0.00, 1.50)

The rows correspond to deployment strategies as defined in the main text. The relative effectiveness during the first 10 y of the program is computed as the proportion of events averted assuming zero efficacy of incomplete courses, divided by the proportion of events averted assuming the reference efficacy of incomplete courses. The minima and maxima are computed over all 14 models and three simulations for each model. All simulations refer to the 20-ibpa transmission setting.

In the case of EPI-based interventions, where assumed coverage for each dose is high, the effect of reducing the efficacy of incomplete courses is small, because most recipients are fully vaccinated. However, with school vaccination and mass vaccination, the simulations lead to substantial numbers of incompletely vaccinated recipients, leading to a substantial reduction in effectiveness if partially vaccinated individuals have no protection.

In all these simulations, as in those reported above, the recipients of each dose are independently sampled from the population. This leads to a very low proportion of individuals receiving the complete course and may be unduly pessimistic. In practice it is likely that the same individuals will tend to comply with the administration of each dose of vaccine, so that wastage due to incomplete courses will be much less than in these simulations. A full analysis considering intermediate levels of efficacy for the first and second doses of vaccine would also need to consider different levels of correlation in receipt of multiple doses.

## Discussion

We originally anticipated that in low transmission settings the low disease burden might limit the impact of RTS,S vaccination, but conversely we might have expected that at low transmission, a herd immunity effect would add to the benefits. Given the consequent uncertainty about the balance of these effects, we were reluctant to rely on predictions from a single model because it was unclear how much the outcomes depend on model structure. The present analysis shows that an assemblage of multiple models, encompassing a wide range of assumptions about decay of immunity and heterogeneity, provide similar predictions. These simulations all indicate that high coverage vaccination strategies will be relatively effective at very low transmission levels, while EPI vaccination will give similar benefits across a wide range of settings.

The computationally intensive approach we adopted is more robust than using a single model. For instance, the greater health impact of vaccination at lower transmission intensities arises because averting an infection leads to a penalty in terms of acquisition of immunity. Averting an inoculation at high exposure may therefore simply delay the clinical response a short time until the next inoculation arrives. However, one common assumption is that vaccination should avert more morbidity and mortality at high transmission, because there is more to avert. Without a set of models we would have been quite unable to decide between these two arguments, and without a range of model structures we would not have known whether the clear dependence of predicted health outcomes on transmission intensity was a specific quirk of one particular model.

Contrary to our prior expectation, the envelopes enclosing the predicted transmission and overall morbidity impacts for the very low transmission setting were narrower, implying less model uncertainty. After completion of our analyses, it seems likely that this is because natural immunity, and hence the details of how it is modeled, is less important at low EIR. Evidently, assumptions about between host heterogeneity also have little effect on the simulated health impact of vaccination programs. We can now make relatively robust statements about the merits of a range of different deployment strategies in very low transmission settings, though there are many variants still to analyze (such as initial mass vaccination with keep-up via EPI and/or school-based vaccination).

The results support previous research [6],[38],[49] that suggests that RTS,S will have less impact in high transmission settings than in lower ones, largely because in the former, morbidity will not be averted, but rather will be delayed. This might be interpreted in the field as decaying efficacy or as “rebound” effects. These findings correspond to those of studies using the base model only [28], which found that EPI vaccination would have the greatest effectiveness around an EIR of 11 ibpa. This impact of EPI is small, however, compared to the simulated impacts of community-wide administration of RTS,S at 2 ibpa. At high EIR, malaria is a disease of very young children, and EPI is likely to be the most cost-effective deployment strategy, but at low exposure levels, all ages of hosts are affected, so an EPI program may miss much of the disease burden [50].

Mass vaccination, on the other hand, will avert disease in all age groups, and this is partly why the simulations indicate a substantially greater impact at 2 ibpa than at the higher exposure level. Large populations now live in areas with very low transmission, in particular cities [51] and highland fringe areas. Targeting mass vaccination to such specific settings could deliver much greater health benefits than introducing RTS,S into the nation-wide EPI. These benefits are observed even if coverage is modest, so that, over the long term, the number of doses administered is no greater than in a school vaccination program, though these effects will depend on the (unknown) efficacy of incomplete courses of vaccination, and on the extent to which it is the same people who receive all doses. Mass vaccination achieves a herd immunity effect by vaccinating a large proportion of the population early on, while EPI or school-based strategies do not achieve this. This benefit of front-loading a vaccination program contrasts with other malaria control strategies, such as indoor residual spraying, that must be sustained continuously at high coverage if transmission is to be prevented from rapidly reverting towards pre-intervention levels.

Programs may well be reluctant to consider mass delivery of multiple-dose vaccines, because of the logistic complexity it would entail, and the danger, inherent in any intermittently intense activity, of disrupting routine health services. Before such an approach can be adopted, many questions need to be addressed. In particular, if more people are eligible for vaccination, the efficacy of incomplete vaccination courses will probably be more important. Uncertainty about the persistence of protection is also a critical problem for strategies depending on herd immunity and on booster vaccination, while duration of protection is less critical with EPI strategies, providing it is long enough to carry children through the first few years of life, when the case fatality rate is highest. The list of questions becomes even longer when the feasibility, economics, and equity of varying vaccination strategies at the sub-national level are considered.

In the real world, when average EIR values become very low, transmission becomes unstable and epidemic. None of our simulations reproduce epidemic malaria, which would have given rise to added variability between simulations in Figure 3. This suggests that vaccines will reduce transmission in a stable way, rather than adding to the instability. Suppression of epidemics in previously unstable transmission settings may therefore be a bonus of pre-erythrocytic vaccination. These considerations also speak for the applicability of RTS,S vaccination in low transmission settings outside the African continent—in particular in South America and Southeast Asia, where low levels of transmission are the norm [52]—where RTS,S vaccination may be a useful adjunct to effective surveillance.

The insensitivity of the results to different model assumptions about transmission heterogeneity suggests that this is not an important factor influencing vaccine impact; however, transmission heterogeneity does affect efficacy estimates in trials (Figure 2), and so cannot be ignored in inferring the underlying efficacy. The main analyses in this paper assume acceptable duration of protection (the consequences of shorter efficacy have already been explored extensively [6]). All the models in the current ensemble also assume perfect mixing in the mosquito population, and there is a need for further model formulations to evaluate the implications of this assumption. Perfect mixing of mosquitoes may well be close to the reality of some transmission settings, since focal vector control interventions, such as insecticide-treated nets used against the *Anopheles gambiae* complex [53]–[56], can have effects over large areas, suggesting considerable vector mobility. Mosquito mobility may be much lower in urban settings [16], in highland fringe areas, and with weaker vectors. Highly focal transmission would be expected to be associated with lower average disease incidence because many hosts would never be exposed [15]. Some of the other standard assumptions about vectors are debatable (such as the insensitivity of mosquito feeding behavior to infection status [57]) and could also be varied, to support analyses of vector control interventions and of integrated control programs, though these would not affect predictions of the impact of vaccination. The ensemble could also be strengthened by including alternative sub-models for pathogenesis, and exploring a wider range of models of immunity.

The present paper thus reports on only the first steps of assembling and analyzing an ensemble for predicting general effects of possible malaria intervention strategies and for highlighting data needs and uncertainties. Model ensembles are often used to improve the precision of model predictions, but so far we did not analyze the predictive power of the different models, or how best to combine their outputs statistically to obtain unbiased point and interval estimates for the predictions. These challenges will require further analysis, which must take into consideration the relatedness of the models. All this needs to be complemented with economic analyses to provide a rational basis for decision making for national integrated control and elimination programs.

### Conclusions

The ensemble modeling approach provides more robust outcomes than single models, and our analyses suggest that such an approach produces greater confidence in predictions of health effects for lower malaria transmission settings than for higher ones. This study suggests that targeted mass vaccination with RTS,S in low transmission settings may be more efficient than national-level introduction via EPI programs, but there remains a need to analyze the feasibility and economics of such strategies and the circumstances in which vaccination will avert epidemics.

## Supporting Information

Figure S1 **Simulated entomological inoculation rates over time.** The columns correspond to the initial EIR values, and the rows to the vaccination strategies simulated. The thick lines correspond to the median across all simulations of the EIR; the grey area is the envelope delimited by the 2.5 and 97.5 percentiles of the complete set of simulations.
(JPG)

Figure S2 **Age prevalence during the tenth year of follow-up.** Interventions and transmission settings as in Figure S1. The lines correspond to the median values of the five simulations for each model within the ensemble of the prevalence, computed from values averaged within each simulation over the full year; the grey area is the envelope delimited by the 2.5 and 97.5 percentiles of the full set of simulations.
(JPG)

Figure S3 **Age incidence curves during the tenth year of follow-up.** Interventions and transmission settings as in Figure S1. The lines correspond to the median values of the five simulations for each model within the ensemble of the incidence of clinical episodes, computed from values averaged within each simulation over the full year; the grey area is the envelope delimited by the 2.5 and 97.5 percentiles of the full set of simulations.
(JPG)

Figure S4 **Number of clinical episodes averted.** The columns correspond to the initial EIR values, and the rows to the vaccination strategies simulated. The lines correspond to the median values of the five simulations for each model within the ensemble of the incidence of clinical episodes; the grey area is the envelope delimited by the 2.5 and 97.5 percentiles of the full set of simulations.
(JPG)

Figure S5 **Age incidence of severe disease during the tenth year of follow-up.** Interventions and transmission settings as in Figure S1. The lines correspond to the median values of the five simulations for each model within the ensemble of the incidence of severe disease, computed from values averaged within each simulation over the full year; the grey area is the envelope delimited by the 2.5 and 97.5 percentiles of the full set of simulations.
(JPG)

Figure S6 **Age incidence of mortality during the tenth year of follow-up.** Interventions and transmission settings as in Figure S1. The lines correspond to the median values of the five simulations for each model within the ensemble of the incidence of mortality, computed from values averaged within each simulation over the full year; the grey area is the envelope delimited by the 2.5 and 97.5 percentiles of the full set of simulations.
(JPG)

Figure S7 **Number of severe episodes averted.** The columns correspond to the initial EIR values, and the rows to the vaccination strategies simulated. The lines correspond to the median values of the five simulations for each model within the ensemble of the number of severe episodes averted; the grey area is the envelope delimited by the 2.5 and 97.5 percentiles of the full set of simulations.
(JPG)

Figure S8 **Number of malaria-related deaths averted.** The columns correspond to the initial EIR values, and the rows to the vaccination strategies simulated. The lines correspond to the median values of the five simulations for each model within the ensemble of the number of deaths averted; the grey area is the envelope delimited by the 2.5 and 97.5 percentiles of the full set of simulations.
(JPG)

Text S1 **Description of models.**
(DOC)

Text S2 **Plots of model fit to field data: R0063.**
(PDF)

Text S3 **Plots of model fit to field data: R0065.**
(PDF)

Text S4 **Plots of model fit to field data: R0068.**
(PDF)

Text S5 **Plots of model fit to field data: R0111.**
(PDF)

Text S6 **Plots of model fit to field data: R0115.**
(PDF)

Text S7 **Plots of model fit to field data: R0121.**
(PDF)

Text S8 **Plots of model fit to field data: R0125.**
(PDF)

Text S9 **Plots of model fit to field data: R0131.**
(PDF)

Text S10 **Plots of model fit to field data: R0132.**
(PDF)

Text S11 **Plots of model fit to field data: R0133.**
(PDF)

Text S12 **Plots of model fit to field data: R0670.**
(PDF)

Text S13 **Plots of model fit to field data: R0674.**
(PDF)

Text S14 **Plots of model fit to field data: R0678.**
(PDF)

## Abbreviations

EIR: entomological inoculation rate
EPI: Expanded Programme on Immunization
ibpa: infectious bites per person per annum

## References

[1] London School of Hygiene and Tropical Medicine Public Health Forum (1992). Malaria: waiting for the vaccine.

[2] Bejon P, Lusingu J, Olotu A, Leach A, Lievens M (2008). Efficacy of RTS,S/AS01E vaccine against malaria in children 5 to 17 months of age.. N Engl J Med.

[3] Abdulla S, Oberholzer R, Juma O, Kubhoja S, Machera F (2008). Safety and immunogenicity of RTS,S/AS02D malaria vaccine in infants.. N Engl J Med.

[4] Ballou WR (2009). The development of the RTS,S malaria vaccine candidate: challenges and lessons.. Parasite Immunol.

[5] Smith T, Killeen GF, Maire N, Ross A, Molineaux L (2006). Mathematical modeling of the impact of malaria vaccines on the clinical epidemiology and natural history of *Plasmodium falciparum* malaria: overview.. Am J Trop Med Hyg.

[6] Penny MA, Maire N, Studer A, Schapira A, Smith TA (2008). What should vaccine developers ask? Simulation of the effectiveness of malaria vaccines.. PLoS ONE.

[7] Macdonald G (1957). The epidemiology and control of malaria.

[8] Le Menach A, Takala S, McKenzie FE, Perisse A, Harris A (2007). An elaborated feeding cycle model for reductions in vectorial capacity of night-biting mosquitoes by insecticide-treated nets.. Malar J.

[9] Saul A, Graves PM, Kay BH (1990). A cyclical feeding model for pathogen transmission and its application to determine vectorial capacity from vector infection-rates.. J Appl Ecol.

[10] Chitnis N, Smith T, Steketee R (2008). A mathematical model for the dynamics of malaria in mosquitoes feeding on a heterogeneous host population.. J Biol Dyn.

[11] Greenwood B (2008). Progress in malaria control in endemic areas.. Travel Med Infect Dis.

[12] Hay SI, Rogers DJ, Toomer JF, Snow R (2000). Annual *Plasmodium falciparum* entomological inoculation rates (EIR) across Africa: literature survey, Internet access and review.. Trans R Soc Trop Med Hyg.

[13] Woolhouse ME, Dye C, Etard JF, Smith T, Charlwood JD (1997). Heterogeneities in the transmission of infectious agents: implications for the design of control programs.. Proc Natl Acad Sci U S A.

[14] Bejon P, Williams TN, Liljander A, Noor AM, Wambua J (2010). Stable and unstable malaria hotspots in longitudinal cohort studies in Kenya.. PLoS Med.

[15] Ross A, Smith T (2010). Interpreting malaria age-prevalence and incidence curves: a simulation study of the effects of different types of heterogeneity.. Malar J.

[16] Trape JF, Lefebvre-Zante E, Legros F, Ndiaye G, Bouganali H (1992). Vector density gradients and the epidemiology of urban malaria in Dakar, Senegal.. Am J Trop Med Hyg.

[17] Dongus S, Nyika D, Kannady K, Mtasiwa D, Mshinda H (2009). Urban agriculture and Anopheles habitats in Dar es Salaam, Tanzania.. Geospat Health.

[18] Belizario VY, Saul A, Bustos MD, Lansang MA, Pasay CJ (1997). Field epidemiological studies on malaria in a low endemic area in the Philippines.. Acta Trop.

[19] Gamage-Mendis AC, Carter R, Mendis C, De Zoysa AP, Herath PR (1991). Clustering of malaria infections within an endemic population: risk of malaria associated with the type of housing construction.. Am J Trop Med Hyg.

[20] Tracton MS, Kalnay E (1993). Operational ensemble prediction at the National Meteorological Center: practical aspects.. Weather Forecast.

[21] Grassly N, Morgan M, Walker N, Garnett G, Stanecki K (2004). Uncertainty in estimates of HIV/AIDS: the estimation and application of plausibility bounds.. Sex Transm Infect.

[22] Johnson LF, Alkema L, Dorrington RE (2010). A Bayesian approach to uncertainty analysis of sexually transmitted infection models.. Sex Transm Infect.

[23] Halloran ME, Ferguson NM, Eubank S, Longini IM, Cummings DA (2008). Modeling targeted layered containment of an influenza pandemic in the United States.. Proc Natl Acad Sci U S A.

[24] Brown T, Salomon JA, Alkema L, Raftery AE, Gouws E (2008). Progress and challenges in modelling country-level HIV/AIDS epidemics: the UNAIDS Estimation and Projection Package 2007.. Sex Transm Infect.

[25] Dietz K, Wernsdorfer WH, Mc Gregor I (1988). Mathematical models for transmission and control of malaria.. Malaria, principles and practice of malariology.

[26] Koella JC, Antia R (2003). Epidemiological models for the spread of anti-malarial resistance.. Malar J.

[27] Ross A, Penny M, Maire N, Studer A, Carneiro I (2008). Modelling the impact of intermittent preventive treatment in infants.. PLoS ONE.

[28] Maire N, Shillcutt S, Walker DG, Tediosi F, Smith T (2011). Cost effectiveness of the introduction of a pre-erythrocytic malaria vaccine into the Expanded Program on Immunization in sub-Saharan Africa: analysis of uncertainties using a stochastic individual-based simulation model of *Plasmodium falciparum* malaria.. Value Health.

[29] The MalERA Consultative Group on Modeling (2010). A research agenda for malaria eradication: modeling.. PLoS Med.

[30] Maire N, Smith T, Ross A, Owusu-Agyei S, Dietz K (2006). A model for natural immunity to asexual blood stages of *Plasmodium falciparum* malaria in endemic areas.. Am J Trop Med Hyg.

[31] Ross A, Maire N, Molineaux L, Smith T (2006). An epidemiologic model of severe morbidity and mortality caused by *Plasmodium falciparum*.. Am J Trop Med Hyg.

[32] Smith T, Ross A, Maire N, Rogier C, Trape JF (2006). An epidemiologic model of the incidence of acute illness in Plasmodium falciparum malaria.. Am J Trop Med Hyg.

[33] Smith T, Maire N, Dietz K, Killeen GF, Vounatsou P (2006). Relationship between the entomologic inoculation rate and the force of infection for *Plasmodium falciparum* malaria.. Am J Trop Med Hyg.

[34] Ross A, Killeen GF, Smith T (2006). Relationships of host infectivity to mosquitoes and asexual parasite density in *Plasmodium falciparum*.. Am J Trop Med Hyg.

[35] Tediosi F, Maire N, Smith T, Hutton G, Utzinger J (2006). An approach to model the costs and effects of case management of Plasmodium falciparum malaria in sub-saharan Africa.. Am J Trop Med Hyg.

[36] Maire N, Aponte JJ, Ross A, Thompson R, Alonso P (2006). Modeling a field trial of the RTS,S/AS02A malaria vaccine.. Am J Trop Med Hyg.

[37] Smith T, Maire N, Ross A, Penny M, Chitnis N (2008). Towards a comprehensive simulation model of malaria epidemiology and control.. Parasitology.

[38] Maire N, Tediosi F, Ross A, Smith T (2006). Predictions of the epidemiologic impact of introducing a pre-erythrocytic vaccine into the expanded program on immunization in sub-Saharan Africa.. Am J Trop Med Hyg.

[39] White MT, Griffin JT, Drakeley CJ, Ghani AC (2010). Heterogeneity in malaria exposure and vaccine response: implications for the interpretation of vaccine efficacy trials.. Malar J.

[40] Olotu A, Lusingu J, Leach A, Lievens M, Vekemans J (2011). Efficacy of RTS,S/AS01E malaria vaccine and exploratory analysis on anti-circumsporozoite antibody titres and protection in children aged 5–17 months in Kenya and Tanzania: a randomised controlled trial.. Lancet Infect Dis.

[41] Sacarlal J, Aide P, Aponte JJ, Renom M, Leach A (2009). Long-term safety and efficacy of the RTS,S/AS02A malaria vaccine in Mozambican children.. J Infect Dis.

[42] Woolhouse ME (1998). Patterns in parasite epidemiology: The peak shift.. Parasitol Today.

[43] Smith T, Hii J, Genton B, Muller I, Booth M (2001). Associations of peak shifts in age-prevalence for human malarias with bed net coverage.. Trans R Soc Trop Med Hyg.

[44] Moorthy VS, Ballou WR (2009). Immunological mechanisms underlying protection mediated by RTS,S: a review of the available data.. Malar J.

[45] Aide P, Dobano C, Sacarlal J, Aponte JJ, Mandomando I (2011). Four year immunogenicity of the RTS,S/AS02(A) malaria vaccine in Mozambican children during a phase IIb trial.. Vaccine.

[46] Alonso PL, Sacarlal J, Aponte JJ, Leach A, Macete E (2005). Duration of protection with RTS,S/AS02A malaria vaccine in prevention of *Plasmodium falciparum* disease in Mozambican children: single-blind extended follow-up of a randomised controlled trial.. Lancet.

[47] Asante KP, Abdulla S, Agnandji S, Lyimo J, Vekemans J (2011). Safety and efficacy of the RTS,S/AS01E candidate malaria vaccine given with expanded-programme-on-immunisation vaccines: 19 month follow-up of a randomised, open-label, phase 2 trial.. Lancet Infect Dis.

[48] Carneiro I, Roca-Feltrer A, Griffin JT, Smith L, Tanner M (2010). Age-patterns of malaria vary with severity, transmission intensity and seasonality in sub-Saharan Africa: a systematic review and pooled analysis.. PLoS ONE.

[49] Griffin JT, Hollingsworth TD, Okell LC, Churcher TS, White M (2010). Reducing *Plasmodium falciparum* malaria transmission in Africa: a model-based evaluation of intervention strategies.. PLoS Med.

[50] Chandramohan D, Webster J, Smith L, Awine T, Owusu-Agyei S (2007). Is the Expanded Programme on Immunisation the most appropriate delivery system for intermittent preventive treatment of malaria in West Africa?. Trop Med Int Health.

[51] Keiser J, Utzinger J, De Castro MC, Smith TA, Tanner M (2004). Urbanization in sub-Saharan Africa and implication for malaria control.. Am J Trop Med Hyg.

[52] Hay SI, Guerra CA, Gething PW, Patil AP, Tatem AJ (2009). A world malaria map: Plasmodium falciparum endemicity in 2007.. PLoS Med.

[53] Binka F, Indome F, Smith T (1998). Impact of spatial distribution of permethrin-impregnated bed nets on child mortality in rural northern Ghana.. Am J Trop Med Hyg.

[54] Hawley WA, Phillips-Howard P, ter Kuile F, Terlouw DJ, Vulule JM (2003). Community-wide effects of permethrin-treated bed nets on child mortality and malaria morbidiy in western Kenya.. Am J Trop Med Hyg.

[55] Howard SC, Omumbo J, Nevill C, Some ES, Donnelly CA (2000). Evidence for a mass community effect of insecticide-treated bednets on the incidence of malaria on the Kenyan coast.. Trans R Soc Trop Med Hyg.

[56] Gosoniu L, Vounatsou P, Tami A, Nathan R, Grundmann H (2008). Spatial effects of mosquito bednets on child mortality.. BMC Public Health.

[57] Anderson RA, Knols B, Koella JC (2000). Plasmodium falciparum sporozoites increase feeding-associated mortality of their mosquito hosts Anopheles gambiae s.l.. Parasitology.

[58] Trape JF, Rogier C (1996). Combating malaria morbidity and mortality by reducing transmission.. Parasitol Today.

