# Supplementary figures and images for: Ensemble Modeling of the Likely Public Health Impact of a Pre-Erythrocytic Malaria Vaccine
Source: PLoS Med. 2012 Jan 17;9(1):e1001157. doi: 10.1371/journal.pmed.1001157 (PMC3260300; doi:10.1371/journal.pmed.1001157)

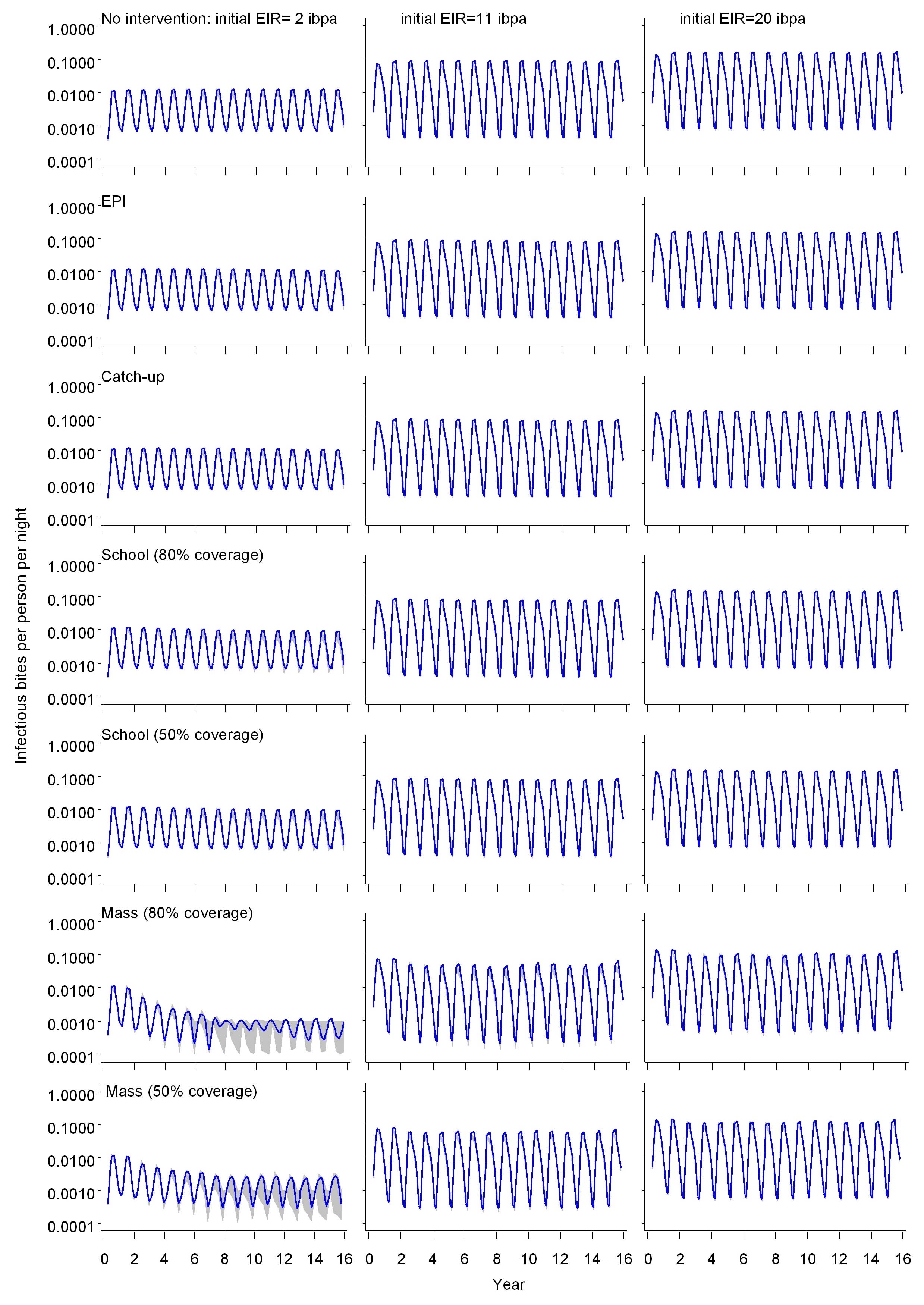

Supplement: Figure S1 — Simulated entomological inoculation rates over time. The columns correspond to the initial EIR values, and the rows to the vaccination strategies simulated. The thick lines correspond to the median across all simulations of the EIR; the grey area is the envelope delimited by the 2.5 and 97.5 percentiles of the complete set of simulations. (JPG) [file pmed.1001157.s001.jpg]

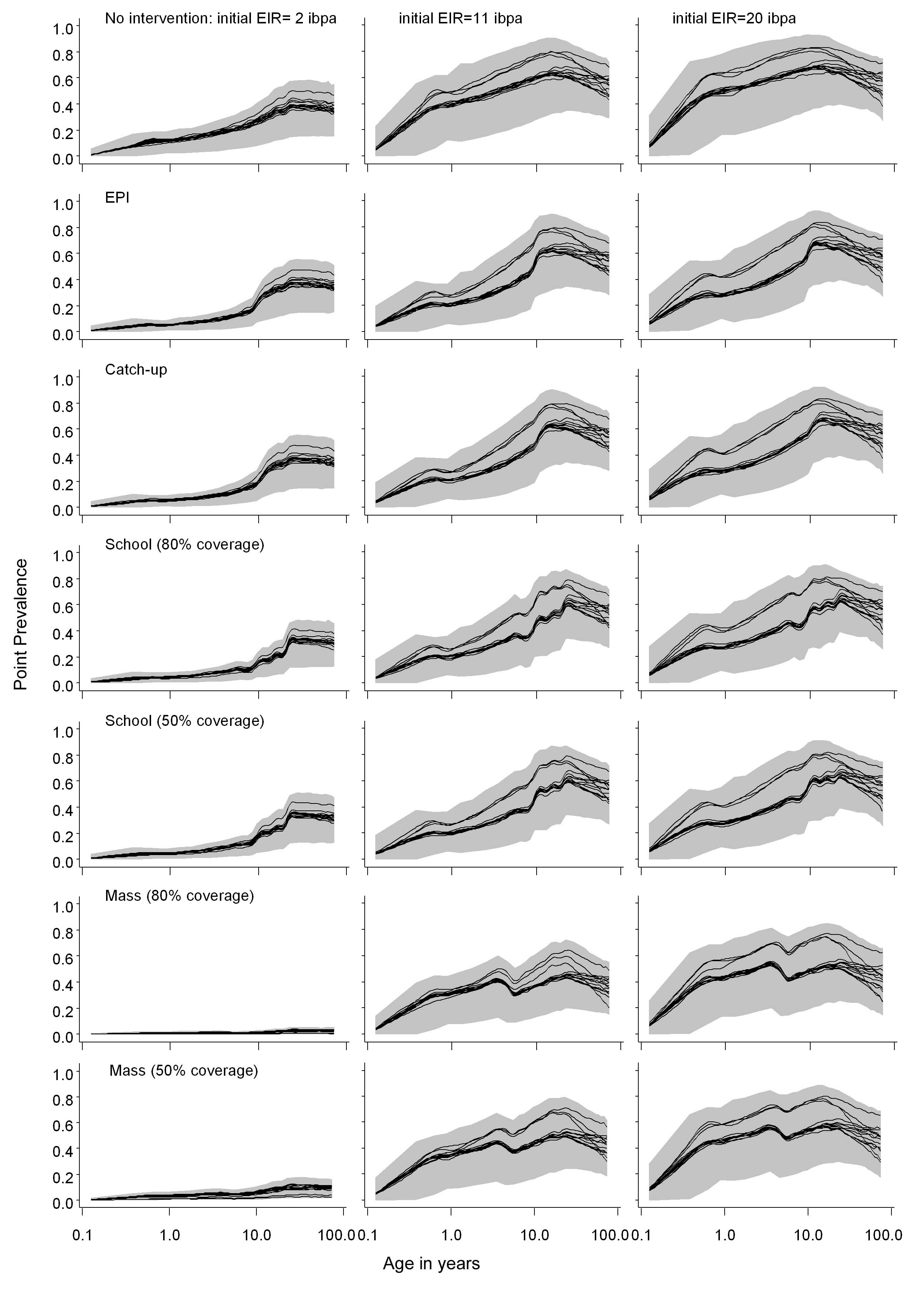

Supplement: Figure S2 — Age prevalence during the tenth year of follow-up. Interventions and transmission settings as in Figure S1. The lines correspond to the median values of the five simulations for each model within the ensemble of the prevalence, computed from values averaged within each simulation over the full year; the grey area is the envelope delimited by the 2.5 and 97.5 percentiles of the full set of simulations. (JPG) [file pmed.1001157.s002.jpg]

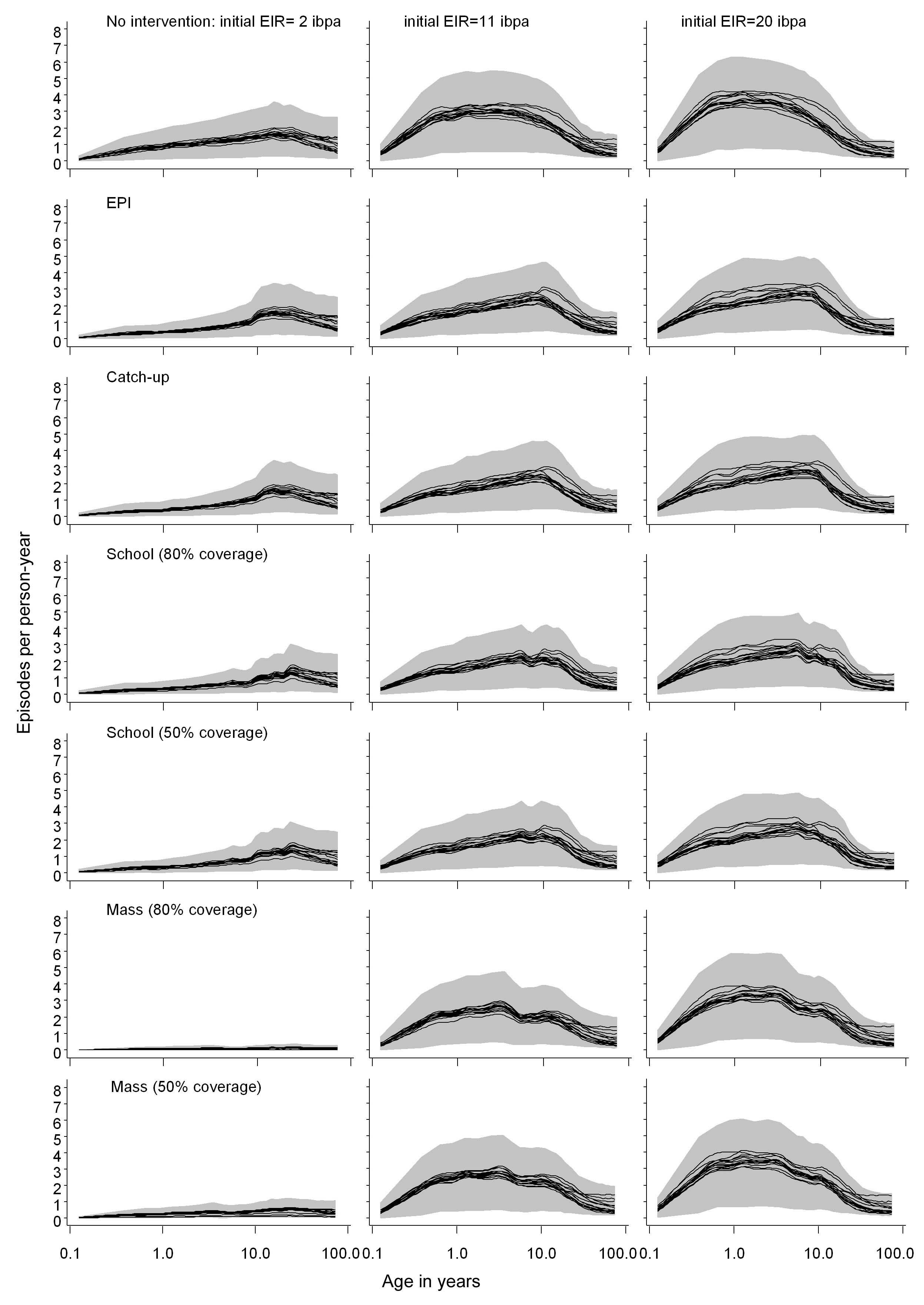

Supplement: Figure S3 — Age incidence curves during the tenth year of follow-up. Interventions and transmission settings as in Figure S1. The lines correspond to the median values of the five simulations for each model within the ensemble of the incidence of clinical episodes, computed from values averaged within each simulation over the full year; the grey area is the envelope delimited by the 2.5 and 97.5 percentiles of the full set of simulations. (JPG) [file pmed.1001157.s003.jpg]

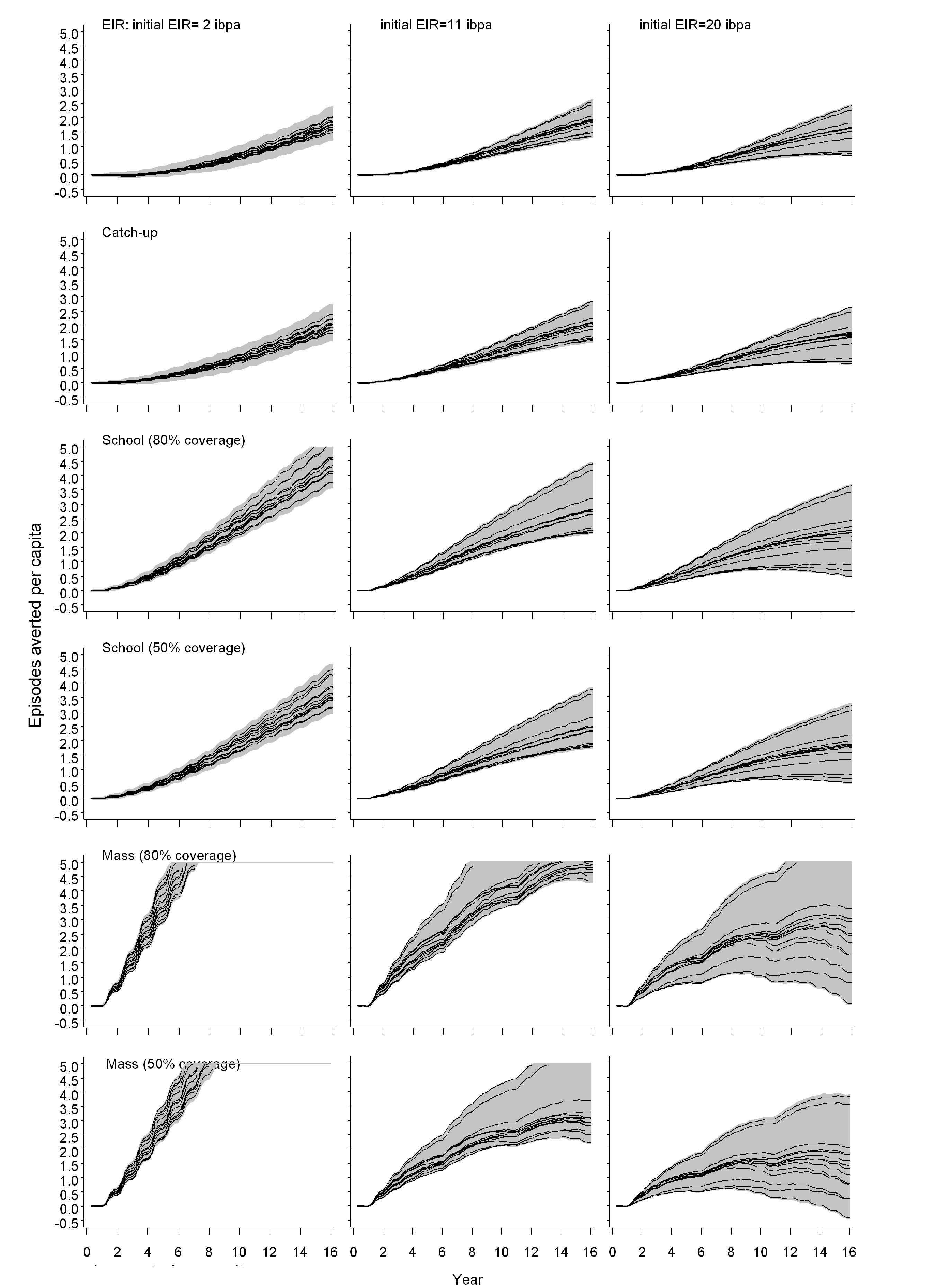

Supplement: Figure S4 — Number of clinical episodes averted. The columns correspond to the initial EIR values, and the rows to the vaccination strategies simulated. The lines correspond to the median values of the five simulations for each model within the ensemble of the incidence of clinical episodes; the grey area is the envelope delimited by the 2.5 and 97.5 percentiles of the full set of simulations. (JPG) [file pmed.1001157.s004.jpg]

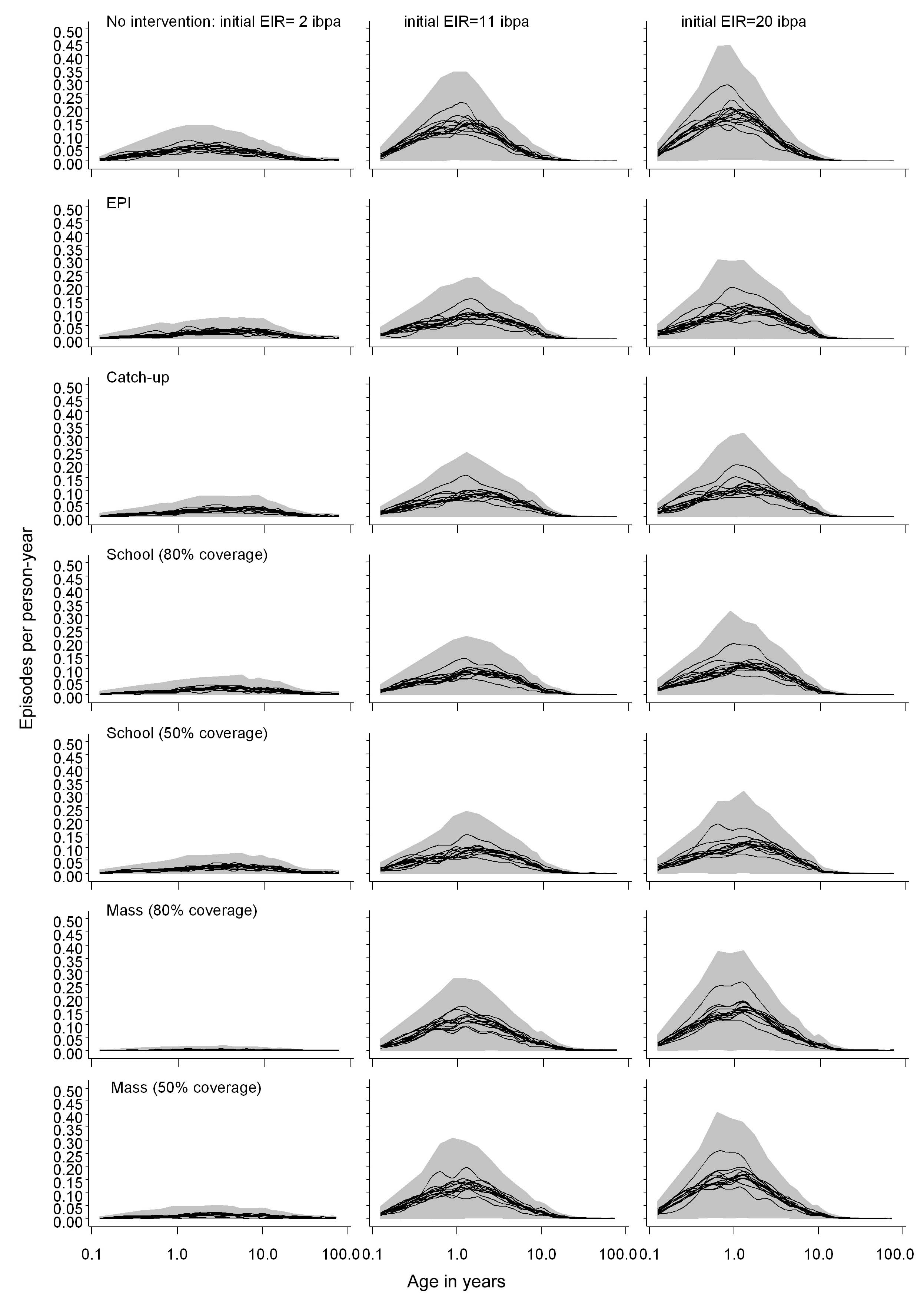

Supplement: Figure S5 — Age incidence of severe disease during the tenth year of follow-up. Interventions and transmission settings as in Figure S1. The lines correspond to the median values of the five simulations for each model within the ensemble of the incidence of severe disease, computed from values averaged within each simulation over the full year; the grey area is the envelope delimited by the 2.5 and 97.5 percentiles of the full set of simulations. (JPG) [file pmed.1001157.s005.jpg]

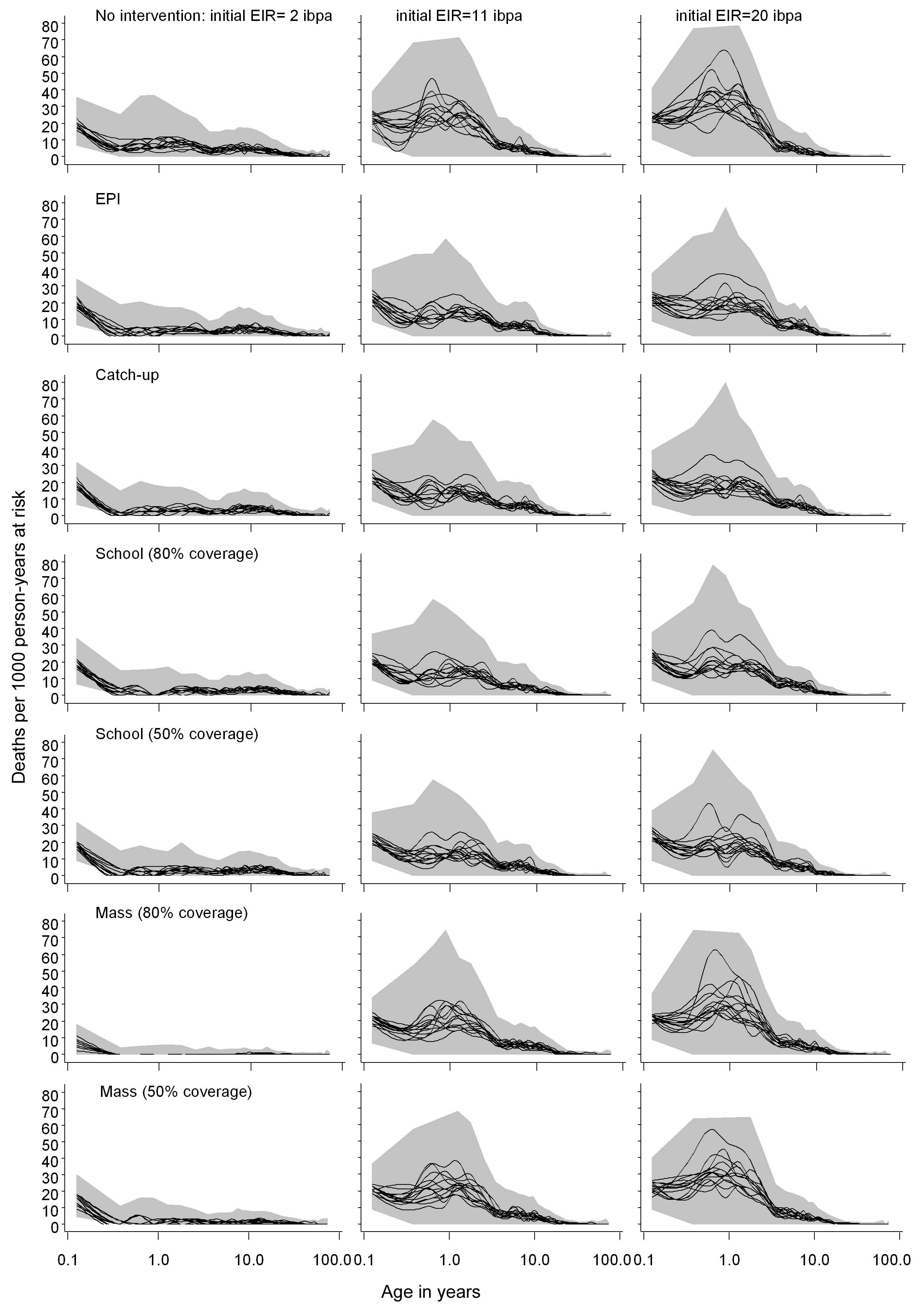

Supplement: Figure S6 — Age incidence of mortality during the tenth year of follow-up. Interventions and transmission settings as in Figure S1. The lines correspond to the median values of the five simulations for each model within the ensemble of the incidence of mortality, computed from values averaged within each simulation over the full year; the grey area is the envelope delimited by the 2.5 and 97.5 percentiles of the full set of simulations. (JPG) [file pmed.1001157.s006.jpg]

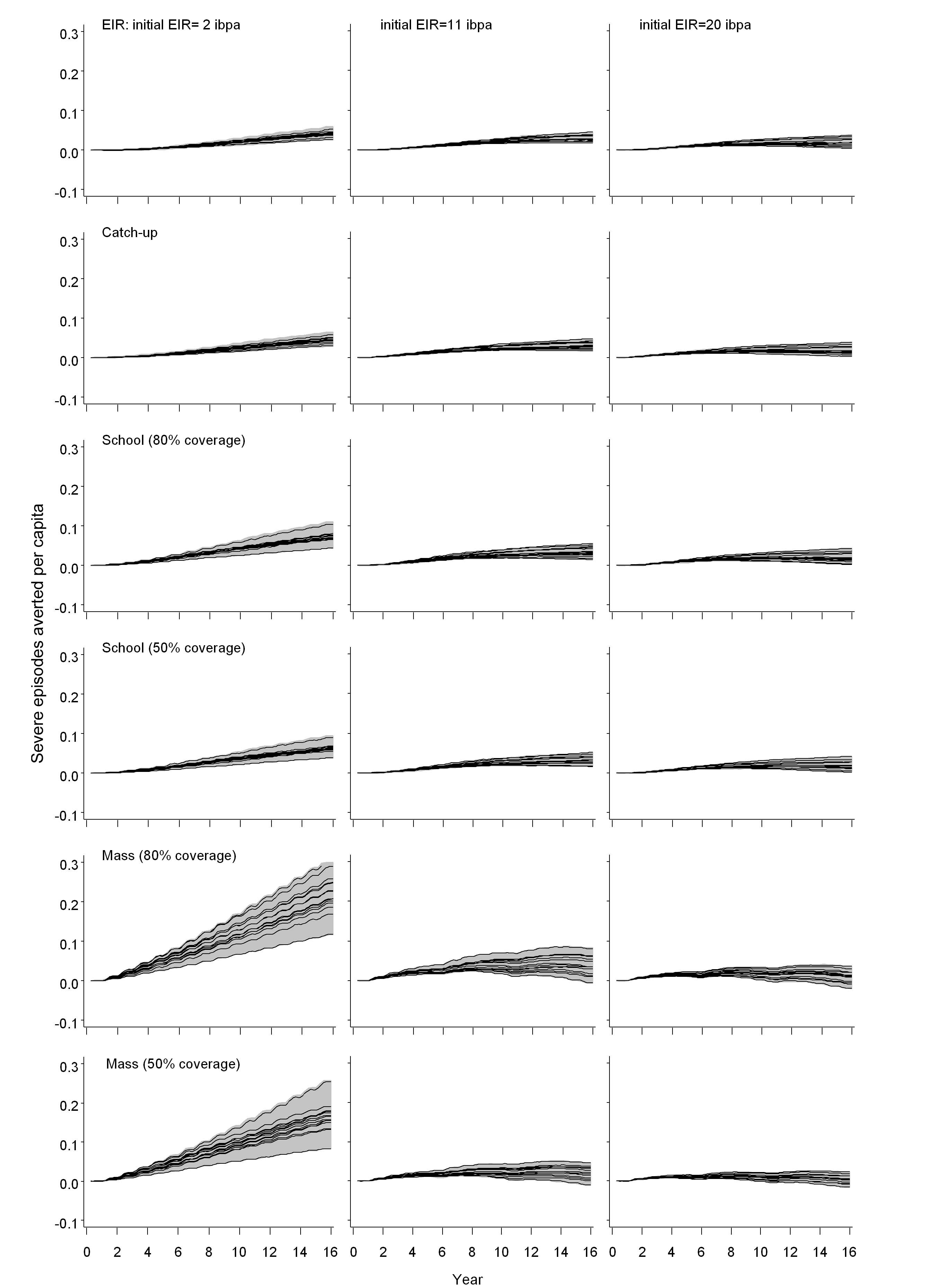

Supplement: Figure S7 — Number of severe episodes averted. The columns correspond to the initial EIR values, and the rows to the vaccination strategies simulated. The lines correspond to the median values of the five simulations for each model within the ensemble of the number of severe episodes averted; the grey area is the envelope delimited by the 2.5 and 97.5 percentiles of the full set of simulations. (JPG) [file pmed.1001157.s007.jpg]

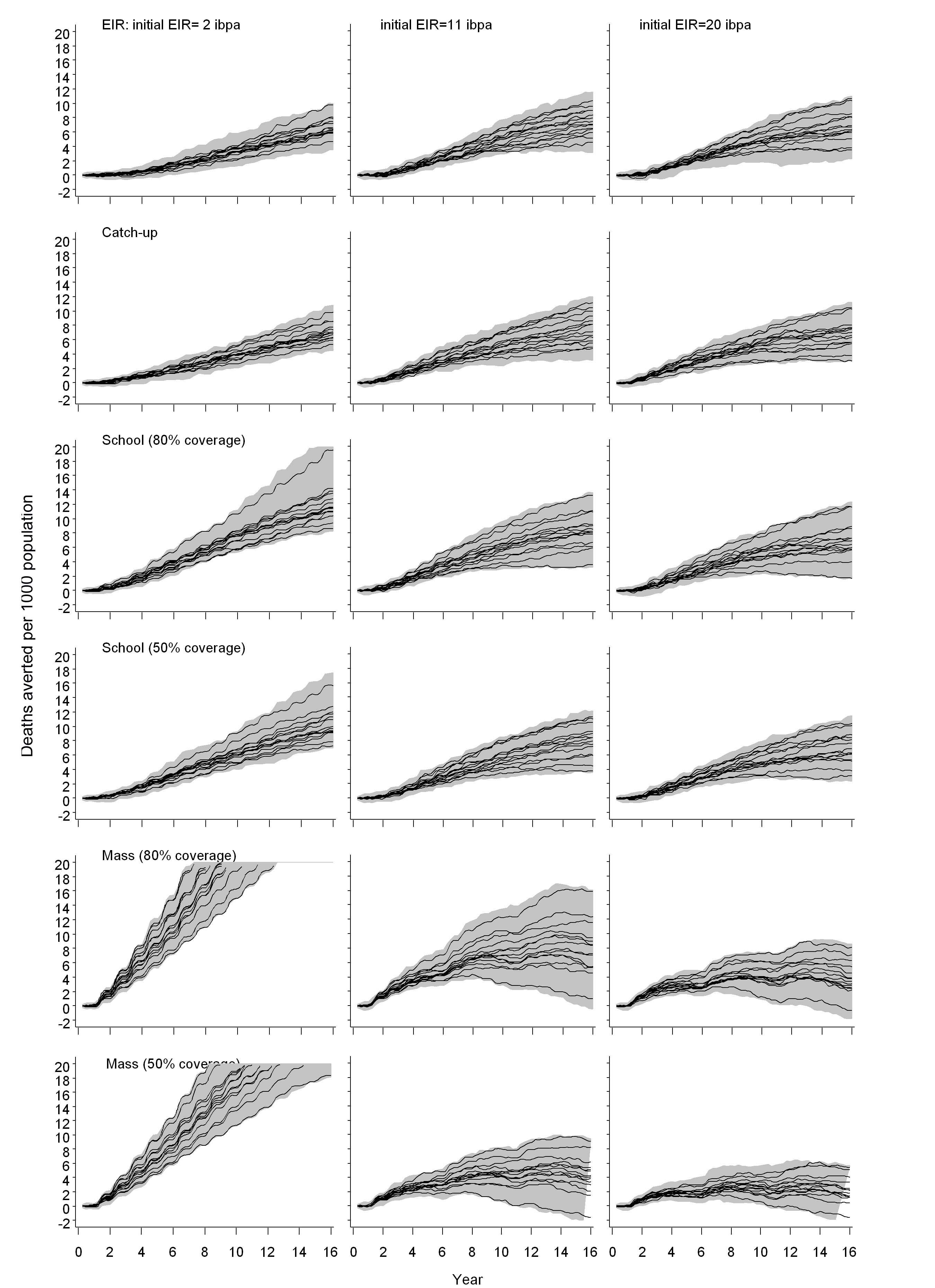

Supplement: Figure S8 — Number of malaria-related deaths averted. The columns correspond to the initial EIR values, and the rows to the vaccination strategies simulated. The lines correspond to the median values of the five simulations for each model within the ensemble of the number of deaths averted; the grey area is the envelope delimited by the 2.5 and 97.5 percentiles of the full set of simulations. (JPG) [file pmed.1001157.s008.jpg]
