# Supplementary material for: Ensemble Modeling of the Likely Public Health Impact of a Pre-Erythrocytic Malaria Vaccine
Source: PLoS Med. 2012 Jan 17;9(1):e1001157. doi: 10.1371/journal.pmed.1001157 (PMC3260300; doi:10.1371/journal.pmed.1001157)
Supplement: Text S1 — Description of models. (DOC) [file pmed.1001157.s009.doc]

# Description of models

### Common features of the models

The models used are all discrete time micro-simulations of malaria in humans, based on the published model used in our previous modeling of malaria vaccines[1], which we refer to as the base model. The model for infection of the vector as a function of parasite densities[2] (and hence for the effect of vaccination on onward transmission), the case-management model[3], the model of vaccination by pre-erythrocytic vaccines[4], the models for clinical outcomes and mortality[5,6], and the basic design of the scenarios used for predicting impacts[3,7] all correspond to the previous descriptions, which are accompanied by detailed rationales for the formulations used.

For all models, the number of infections $h(i,t)$ introduced in individual *i* in five-day time step *t*, is distributed as:

$h(i,t)\sim Poisson(\lambda(i,t))$ (1)

where the expected number,

$\lambda(i,t)=S(i,t)E_{a}(i,t)$,

$S(i,t)$ is the susceptibility (proportion of inoculations that result in infections) and$E_{a}(i,t)$ is the expected number of entomological inoculations, adjusted for age and individual factors .

### Base Model

In the base model,

$E_{a}(i,t)=E_{max}(t)\frac{A(a(i,t))}{A_{\max}}$ (2)

where *E_max_* (t) refers to the usual measure of the EIR computed from human bait collections on adults and $A()$, the availability to mosquitoes, is assumed to be proportional to average body surface area and to depend only on age $a(i,t)$. The function used to compute $A(a(i,t))$ increases with age up to age 20 years where it reaches a value of $A_{\max}$.

To capture observed relationships between infection and measured entomological exposure, the susceptibility is then:

$S(i,t)=(S_{\infty}+\frac{1-S_{\infty}}{1+\frac{E_{a}(i,t)}{E^{*}}})(S_{imm}+\frac{1-S_{imm}}{1+\left(\frac{X_{p}(i,t)}{X_{p}^{*}}\right)^{\gamma_{p}}})$ (3)

where are constants and:

$X_{p}(i,t)=\int_{t-a(i,t)}^{t} E_{a}(i,\tau)d\tau$. (4)

Fitting this model to the data of [8] gave values of $S_{\infty}=0.049$, and $E^{*}=0.032$ inoculations/person-night.

The model for each individual infection *j* in host *i* comprises a time series of parasite densities. In a naïve host the expectation of the logarithm of the density of the infection at time point $\tau$ in the course of the infection, $\ln(y_{0}(i,j,\tau))$, is taken from a statistical description of parasite densities in malaria therapy patients[9]. In the presence of previous exposure and co-infection, the expected log density for each concurrent infection is:

$$E\left(\ln\left(y\left(i,j,\tau\right)\right)\right)=D_{y}(i,t)D_{h}(i,t)D_{m}(i,t)\cdot\ln(y_{0}(i,j,\tau))+\ln\left(\frac{D_{x}}{M(i,t)}+1-D_{x}\right)$$

where *M(i,t)* is the total multiplicity of infection and

$D_{y}(i,t)=\frac{1}{1+\frac{X_{y}(i,j,t)}{X_{y}^{*}}}$,

where $X_{y}(i,j,t)=\sum_{t-a}^{t} Y(i,t)-\sum_{t_{0,j}}^{t} y(i,j,\tau)$ (note that a continuous time approximation to this is given in the original publications [1,9]).

and hence measures the cumulative parasite load, and

$D_{h}(i,t)=\frac{1}{1+\frac{X_{h}(i,t)}{X_{h}^{*}}}$,

where $X_{h}(i,t)=\sum_{t-a}^{t} h(i,\tau)-1$, the number of inoculations since birth, excluding the one under consideration, which measures the diversity of inocula experienced by the host up to the time point under consideration.

$$D_{m}(i,t)=1-\alpha_{m}\exp\left(-\frac{0.693a(i,t)}{a_{m}^{*}}\right)$$

which measures the effect of maternal immunity. $X_{y}^{*}$, $X_{h}^{*}$,$D_{x}$, $a_{m}^{*}$, and $\alpha_{m}$, are all constants estimated by fitting to field data as described below.

### Mass action model of the force of infection

The base model assumes that susceptibility is a sigmoidal function of the exposure, which conflicts with the usual mass action assumption for infectious disease transmission. However, if entomological exposure is over-dispersed, infection-exposure relationships similar to those observed in the field can be reconciled with mass action principles, thus providing explanation for the observed non-linear relationship between infection and entomological exposure and hence a more justifiable representation of the infection process than that used in the base model[10]. We simulate this by including random variation in the availability of the human host to mosquitoes. This variation comprises an individual-specific component which is captured by defining for each simulated human a baseline availability, $A_{b}(i)$, which is sampled at birth using:

$$\log(A_{b}(i))\sim Normal(\frac{-\sigma_{b}^{2}}{2},\sigma_{b}^{2})$$

so that $A_{b}(i)$ is log-normally distributed with arithmetic mean 1. The age adjusted EIR at time t, adjusted to the individual is then:

$$E_{b}(i,t)=E_{max}(t)A_{b}(i)A(a(i,t))/A_{\max}$$

Additional log normal variation is introduced at each time step in the simulation to make the expected number of entomological inoculations a function both of the individual and of log-normal noise measured by the variance parameter, $\sigma_{n}^{2}$:

$$\ln\left(E_{a}(i,t)\right)\sim Normal(\ln(E_{b}(i,t))-\frac{\sigma_{n}^{2}}{2},\sigma_{n}^{2})$$

The susceptibility in these models is independent of EIR, i.e.:

$$S(i,t)=S_{\infty}(S_{imm}+\frac{1-S_{imm}}{1+\left(\frac{X_{p}(i,t)}{X_{p}^{*}}\right)^{\gamma_{p}}})$$

Three different parameterisations of this model were considered. In each case, the overall variability in $E_{a}(i,t)$ was constrained to the same total, estimated by fitting to the data of [8].

The three cases comprise models *R0063*, *R0065*, and *R0068* with values of $\sigma_{b}^{2}$ of 0.5, 0.3 and 0.05 corresponding to values of $\sigma_{n}^{2}$ of 0.187, 0.567 and 0.704 respectively. *R0063* thus assigns most variation to be inter-host, *R0068*, assigns the variation predominantly to within host variation, and *R0065* is intermediate.

### Models of decay of natural immunity

The model for natural immunity used in the base model[9], was developed primarily for simulating the epidemiology of malaria in endemic settings, does not allow for any decay of immunity in the absence of exposure. There is substantial evidence that clinical immunity declines in the absence of stimulation, but while decay of antibody levels can be quantified from field data[11,12], it is unclear how these relate to clinical immunity or to the control of parasite densities. There are no direct estimates of the rate of decay of immune control of parasite densities.

To allow for such decay the base model was extended using two alternative algorithms. In both cases the models were parameterised so that in the absence of new exposure, the decayed value is some fixed proportion $\alpha_{b}$ or $\alpha_{c}$ of that at the previous five-day time step.

In models that allow explicit decay of immune effectors, a decay is applied to the two measures of the effective cumulative exposure which become:

$$X_{h}(i,t)=\alpha_{b}\left(\sum_{t-a}^{t} h(i,\tau)-1\right)$$

$X_{y}(i,j,t)=\alpha_{b}\left(\sum_{\tau=t-a}^{t} Y(i,\tau)-\sum_{\tau=t_{0,j}}^{t} y(i,j,\tau)\right)$.

In further models the decay was applied to the function representing the immune status, so that in the limiting case where there is no exposure:

$$D_{h}(i,t)=1-\alpha_{c}\left(1-D_{h}(i,t-1)\right)$$

$$D_{y}(i,t)=1-\alpha_{c}\left(1-D_{y}(i,t-1)\right)$$

Corresponding to:

$$X_{h}(i,t)=\frac{\alpha_{c}X_{h}^{*}X_{h}(i,t-1)}{X_{h}^{*}+\left(1-\alpha_{c}\right)X_{h}(i,t-1)}$$

$$X_{y}(i,t)=\frac{\alpha_{c}X_{y}^{*}X_{y}(i,t-1)}{X_{y}^{*}+\left(1-\alpha_{c}\right)X_{y}(i,t-1)}$$

In the presence of concurrent exposure, the corresponding difference equations for the measures of the effective cumulative exposure are:

$$X_{h}(i,t)=\frac{\alpha_{c}X_{h}^{*}\left(X_{h}(i,t-1)+h(i,t)\right)}{X_{h}^{*}+\left(1-\alpha_{c}\right)\left(X_{h}(i,t-1)+h(i,t)\right)}$$

$$X_{y}(i,j,t)=\frac{\alpha_{c}X_{y}^{*}\left(X_{y}(i,j,t-1)+Y(i,t)-\sum_{\tau=t_{0,j}}^{t} y(i,j,\tau)\right)}{X_{y}^{*}+\left(1-\alpha_{c}\right)\left(X_{y}(i,j,t-1)+Y(i,t)-\sum_{\tau=t_{0,j}}^{t} y(i,j,\tau)\right)}$$

### Discrete heterogeneity in treatment-seeking, co-morbidity or transmission.

We previously extended the base model by introducing heterogeneity in (i) transmission, (ii) co-morbidity and (iii) treatment-seeking probability. Each simulated individual was assigned a status for each of the three kinds of heterogeneity at birth, which they carry throughout their life, structured in each case so that 50% of the population are assigned to each of the high and low status categories, with the values in the base model multiplied by either 1.8 or 0.2 [13]. These heterogeneities were simulated singly and in independent and co-varying pairs so that an individual could, for example, have a high risk of a non-malaria co-morbidity and a low probability of treatment.

In the original publication exploring the effects of these types of heterogeneity[13], the remaining model parameters remained the same as in the base model. For the present analyses the parameters of the base model were re-estimated conditional on the assumed heterogeneities (see Tables 2 & 3). Parameterisations with acceptable overall goodness of fit was achieved only with only 4 of the 11 model structures analysed, corresponding to models *R670* (heterogeneity in susceptibility to co-morbidity); *R673* (correlated heterogeneities in access to treatment and susceptibility to co-morbidity); *R674* (uncorrelated heterogeneities in access to treatment and susceptibility to co-morbidity); and *R678* (heterogeneity in access to treatment).

### Fitting of models to field data

All simulations used the originally published sub-model[2] for infection of the vector, and the sub-model for the force of infection was separately fitted to data from Saradidi, Kenya[8,14]. The remaining parameters, which determining the effects of acquired immunity on parasitological and morbidity outcomes, were estimated by fitting the overall model to field data conditional on these representations of the infection process, and of the description of an infection in the naive host.

The models were all parameterized using the same datasets as were used for calibrating the base model[1,15]. The models were fitted to a further 9 different epidemiological quantities chosen to make the models of acquired immunity identifiable (Table S1). For some of these, large amounts of data are available, and for others data availability was limiting. Overall a total of 61 different datasets, containing variable numbers of data points, contributed data that could be used to fit these models.

Given the number of model parameters (see Table 1 in the original publication of the base model[1]), the search space was of high-dimension. Because the models are microsimulations of individual infections (rather than population average models) the computational demand of evaluating a single parameterization was substantial, (approximately one hundred CPU hours on a standard PC). This precluded the application of estimation methods requiring very large numbers of function evaluations, such as Bayesian Markov chain Monte Carlo approaches. A somewhat *ad hoc* approach to fitting the models was therefore adopted, recognizing that this is unlikely to lead to the global optimum on any specific criterion.

### Weighting of objectives

The parameter estimation process is in principle a multi-objective optimization problem with each of the epidemiological quantities in Table S1 representing one objective. By building a weighted average of the different objectives, this was reduced to a single overall measure of goodness-of-fit statistic, defined as:

$$G\left(\theta\right)=-\sum_{i} w_{i}\sum_{j} L_{ij}(\theta)$$

where$L_{ij}(\theta)$ is the log likelihood for parameter vector $\theta$, epidemiological quantity *i* and dataset *j*, and the weights $w_{i}$were chosen so that different epidemiological quantities contribute approximately equally to $G\left(\theta\right)$.

### Search strategy and computation

The objective functions are non-differentiable and noisy due to the stochastic nature of the simulations, and the existence of local minima cannot be ruled out. The search strategy employed an evolutionary algorithm to address these challenges, with the intention being to obtain a parameterization which approximately reproduced the data in each dimension.

This algorithm was implemented on a distributed computing platform to provide the necessary computing power through volunteered resources (see [http://boinc.berkeley.edu](http://boinc.berkeley.edu/), and http://www.malariacontrol.net). The choice of an appropriate algorithm is challenging in the light of this heterogeneous computing system: in particular, classical synchronous evolutionary algorithms are not suitable to efficiently exploit such a computing resource because the time to evaluate a single parameterization is not predictable[16]. For that reason an asynchronous implementation of a genetic algorithm was used to explore the search space (4): new parameterizations were sampled on-demand from a population of existing solutions. In the genetic algorithm, model parameters were represented as continuous loci, and constrained to positive values. Mutations were implemented by sampling from a log-normal distribution with the parents’ current parameter value as the mean, and the variance proportional to the mean. Both per-loci mutation rates and the sampling range were adaptive, i.e. are inherited from the parent while allowing for mutation in these meta-parameters. Several variations of the algorithm were explored for their performance, including algorithms that allowed for within-locus (averaging parents’ parameter values) and inter-locus (selecting different parameters from different parents) recombination. The basic mutation-only genetic algorithm proved superior in terms of convergence rate compared both to variants allowing for recombination, and to alternative algorithms that were also explored including asynchronous genetic simplex, and asynchronous differential evolution algorithms[17].

### Selection of parameterisations for inclusion in the ensemble

The full search algorithm was implemented separately for each model. $G\left(\theta\right)$ is a stochastic function, which does not converge to a single value, so the genetic algorithm was run until visual inspection of the trace of $G\left(\theta^{(k)}\right)$ against *k* (where $\theta^{(k)}$ is the parameter vector evaluated at iteration *k*), suggested that improvement had ceased. For each of these runs, the parameterization, $\tilde{\theta}$, chosen for further analysis was the one that minimized$G\left(\theta\right)$ among the full set of parameter vectors evaluated.

Model fit was further evaluated by plotting the model predictions next to the field data used in the optimization process. On the basis of visual agreement between fitted values and the data summaries in these plots an adequately fitting parameterization was considered to be one with $G\left(\tilde{\theta}\right)<1.2$. The full set of these plots for the models included in the ensemble are included as Texts S2- S14, with the sets of plots, fitting runs, and models identified by the same reference numbers. Each of the plots within these sets corresponds to a figure in the original publications describing the base model, as referenced in Table S1. Further details of the data sources and data transformations are given in these original publications.

**Table S1. Epidemiological quantities and data sources used for parameterizing models.**

| **Epidemiological quantity** | **Panel in suppl-emen-tary figures** | **Data**  **Sour-ces** | **Number of scen-arios^a^** | **Number of data points^b^** | **Publi- cation of the fitting of the base model** | **Figure number in original publi-cation** | **Assumed distribution of data**  **about model prediction** | **Weight-ing in overall goodness of fit statistic** |
| --- | --- | --- | --- | --- | --- | --- | --- | --- |
| Age- and seasonal patterns of prevalence of infection^c^ | X1 | [18] | 6 | 563 | [9] | Fig. 4 | Binomial | 0.001 |
| Age- and seasonal patterns of parasite density^c^ | X2 | [18] | 6 | 563 | [9] | Fig. 6 | Log normal | 0.01 |
| Age pattern of number of concurrent infections^c^ | X3 | [9,19] | 1 | 12 | [9] | Fig. 5 | Poisson | 0.01 |
| Age pattern of incidence of clinical malaria^c,e^ | X4 | [20,21] | 3 | 31 | [22] | Fig. 1 | Log normal | 1 |
| Age pattern of threshold parasite density for clinical attacks^c^ | X5 | [23] | 1 | 13 | [22] | Fig. 4 | Log normal | 1 |
| Hospitalisation rate in relation to prevalence in children | X6 | See [6] | 26 | 10^d^ | [6] | Fig. 2 | Log normal | 2 |
| Age pattern of hospitalisation^c,f^ | X7 | [24] | 4 | 12 | [6] | Fig. 4 | Log normal | 2 |
| Malaria specific mortality in children^c^ | X8 | [25] | 9 | 9 | [6] | Fig. 8 | Log normal | 1 |
| Infant mortality rate^c^ | X9 | See [6] | 11 | 11 | [6] | Fig. 9 | Log normal | 10 |

**^a^**Some scenarios are used to predict several outcomes, so the total of this column does not equal the total of 61 scenarios involved in fitting the models.

**^b^**The number of data points is the sum over all scenarios and simulated survey periods of the number of age groups into which the data were disaggregated for comparison with the model predictions.

**^c^**In relation to the EIR specified as a seasonal pattern

**^d^**Model predictions for this objective are compared with linear interpolations between the field data points.

**^e^**Panel (a) refers to age-specific data from Senegal[20]; panel (b) refers to data for infants in Idete, Tanzania[21]

**^f^**Panel (a) is the field data, (b) is model predictions.

**References**

1. Smith T, Killeen GF, Maire N, Ross A, Molineaux L, Tediosi F, Hutton G, Utzinger J, Dietz K, Tanner M (2006) Mathematical modeling of the impact of malaria vaccines on the clinical epidemiology and natural history of *Plasmodium falciparum* malaria: Overview. Am J Trop Med Hyg 75: 1-10.

2. Ross A, Killeen GF, Smith T (2006) Relationships of host infectivity to mosquitoes and asexual parasite density in *Plasmodium falciparum* . Am J Trop Med Hyg 75 (Suppl 2): 32-37.

3. Tediosi F, Maire N, Smith T, Hutton G, Utzinger J, Ross A, Tanner M (2006) An approach to model the costs and effects of case management of Plasmodium falciparum malaria in sub-saharan Africa. Am J Trop Med Hyg 75: 90-103.

4. Maire N, Aponte JJ, Ross A, Thompson R, Alonso P, Utzinger J, Tanner M, Smith T (2006) Modeling a field trial of the RTS,S/AS02A malaria vaccine. Am J Trop Med Hyg 75: 104-110.

5. Smith T, Ross A, Maire N, Rogier C, Trape JF, Molineaux L (2006) An epidemiologic model of the incidence of acute illness in Plasmodium falciparum malaria. Am J Trop Med Hyg 75: 56-62.

6. Ross A, Maire N, Molineaux L, Smith T (2006) An epidemiologic model of severe morbidity and mortality caused by *Plasmodium falciparum*. Am J Trop Med Hyg 75: 63-73.

7. Maire N, Tediosi F, Ross A, Smith T (2006) Predictions of the epidemiologic impact of introducing a pre-erythrocytic vaccine into the expanded program on immunization in sub-Saharan Africa. Am J Trop Med Hyg 75: 111-118.

8. Beier JC, Oster CN, Onyango FK, Bales JD, Sherwood JA, Perkins PV, Chumo DK, Koech DV, Whitmire RE, Roberts CR (1994) *Plasmodium falciparum* incidence relative to entomologic inoculation rates at a site proposed for testing malaria vaccines in western Kenya. Am J Trop Med Hyg 50: 529-536.

9. Maire N, Smith T, Ross A, Owusu-Agyei S, Dietz K, Molineaux L (2006) A model for natural immunity to asexual blood stages of *Plasmodium falciparum* malaria in endemic areas. Am J Trop Med Hyg 75: 19-31.

10. Smith TA (2008) Estimation of heterogeneity in malaria transmission by stochastic modelling of apparent deviations from mass action kinetics. Malar J 7: 12. 1475-2875-7-12 [pii];10.1186/1475-2875-7-12 [doi].

11. Cornille-Brogger R, Mathews HM, Storey J, Ashkar TS, Brogger S, Molineaux L (1978) Changing patterns in the humoral immune response to malaria before, during, and after the application of control measures: a longitudinal study in the West African savanna. Bull World Health Organ 56: 579-600.

12. Cook J, Reid H, Iavro J, Kuwahata M, Taleo G, Clements A, McCarthy J, Vallely A, Drakeley C (2010) Using serological measures to monitor changes in malaria transmission in Vanuatu. Malar J 9: 169.

13. Ross A, Smith T (2010) Interpreting malaria age-prevalence and incidence curves: a simulation study of the effects of different types of heterogeneity. Malar J 9: 132.

14. Smith T, Maire N, Dietz K, Killeen GF, Vounatsou P, Molineaux L, Tanner M (2006) Relationships between the entomological inoculation rate and the force of infection for *Plasmodium falciparum* malaria. Am J Trop Med Hyg 75 (Suppl 2): 11-18.

15. Smith T, Maire N, Ross A, Penny M, Chitnis N, Schapira A, Studer A, Genton B, Lengeler C, Tediosi F, de Savigny D, Tanner M (2008) Towards a comprehensive simulation model of malaria epidemiology and control. Parasitology 135: 1507-1516.

16. Desell T, Anderson DP, Magdon-Ismail M, Newberg H, Szymanski BK, Varela CA (2010) An analysis of massively distributed evolutionary algorithms. EEE Congress on Evolutionary Computation (CEC) doi: 10.1109/CEC.2010.5586073: 1-8.

17. Desell, T. (2009) Asynchronous global optimization for massive scale computing [dissertation]. Rensselaer Polytechnic Institute.

18. Molineaux, L. and Gramiccia G. (1980) The Garki Project. Geneva: World Health Organisation.

19. Owusu-Agyei S, Smith T, Beck H-P, Amenga-Etego L, Felger I (2002) Molecular epidemiology of Plasmodium falciparum infections among asymptomatic inhabitants of a holoendemic malarious area in northern Ghana. Trop Med Int Health 7: 421-428.

20. Trape JF, Rogier C (1996) Combating malaria morbidity and mortality by reducing transmission. Parasitol Today 12: 236-240.

21. Kitua A, Smith T, Alonso PL, Masanja H, Urassa H, Menendez C, Kimario J, Tanner M (1996) Plasmodium falciparum malaria in the first year of life in an area of intense and perennial transmission. Trop Med Int Health 1: 475-484.

22. Smith T, Ross A, Maire N, Rogier C, Trape JF, Molineaux L (2006) An epidemiological model of the incidence of acute illness in *Plasmodium falciparum* malaria. Am J Trop Med Hyg 75 (Suppl 2): 56-62.

23. Rogier C, Commenges D, Trape JF (1996) Evidence for an age-dependent pyrogenic threshold of Plasmodium falciparum parasitemia in highly endemic populations. Am J Trop Med Hyg 54: 613-619.

24. Marsh K, Snow R (1999) Malaria transmission and morbidity. Parassitologia 41: 241-246.

25. Snow R, Omumbo J, Lowe B, Molyneux CS, Obiero JO, Palmer A, Weber MW, Pinder M, Nahlen B, Obonyo C, Newbold C, Gupta S, Marsh K (1997) Relation between severe malaria morbidity in children and level of Plasmodium falciparum transmission in Africa [see comments]. Lancet 349: 1650-1654.
