# Supplementary material for: Ensemble Modeling of the Likely Public Health Impact of a Pre-Erythrocytic Malaria Vaccine
Source: PLoS Med. 2012 Jan 17;9(1):e1001157. doi: 10.1371/journal.pmed.1001157 (PMC3260300; doi:10.1371/journal.pmed.1001157)

X1: Age patterns of prevalence of infection

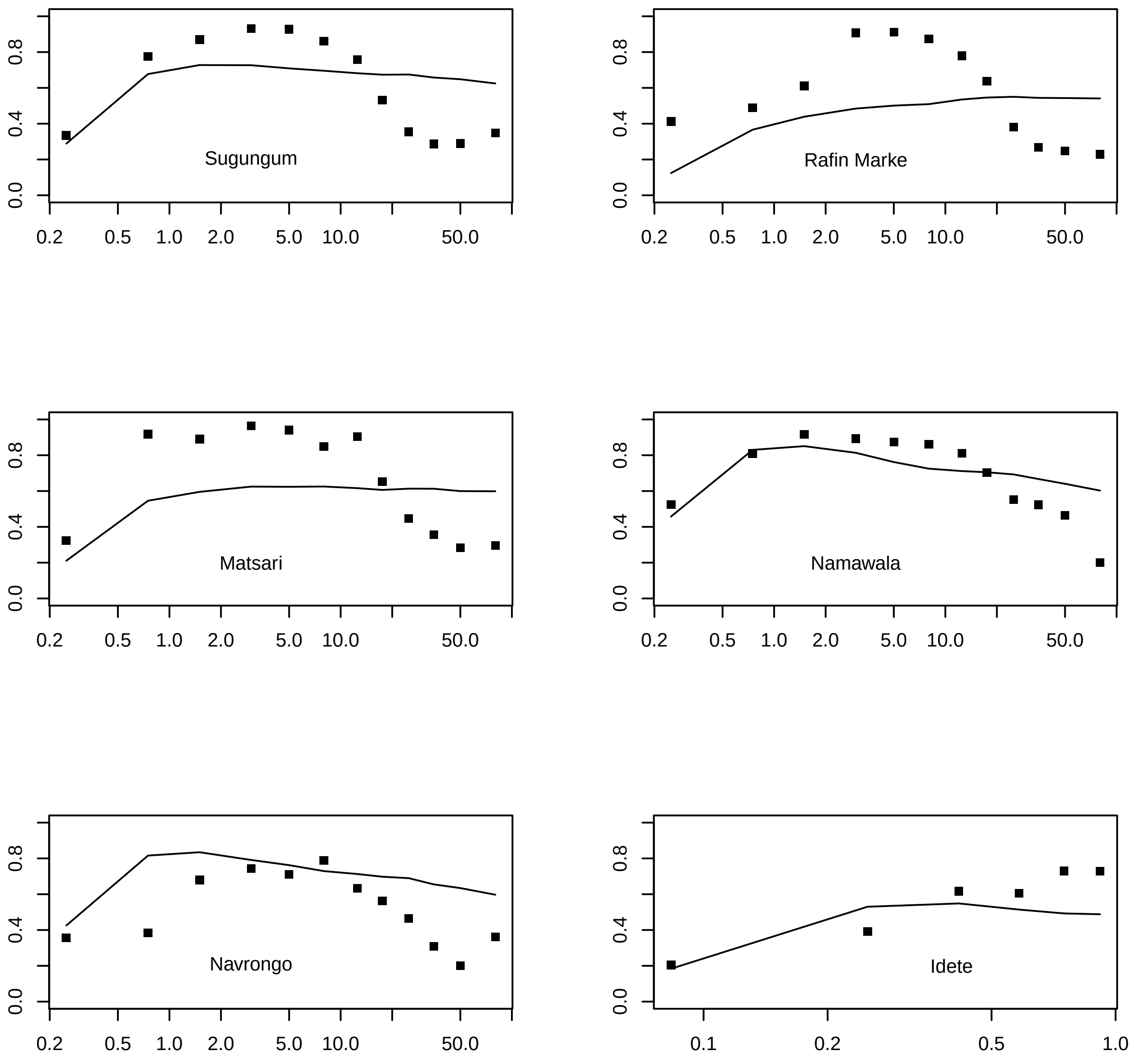

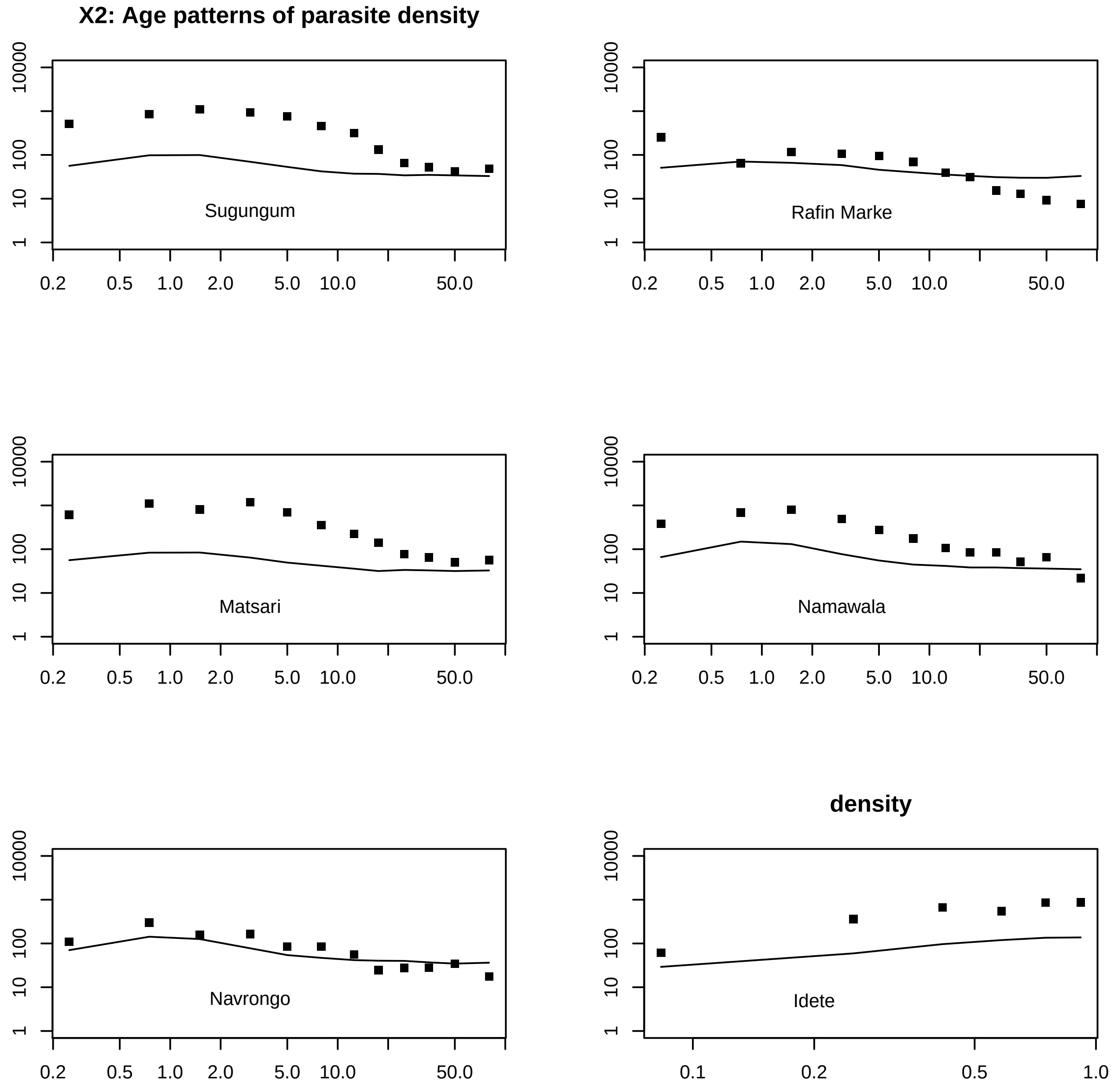

X2: Age patterns of parasite density
Sugungum
Rafin Marke
Matsari
Namawala
Navrongo
density
Idete
1
10
100
10000
0.2
0.5
1.0
2.0
5.0
10.0
50.0
0.1

X3: Age pattern of number of concurrent infections

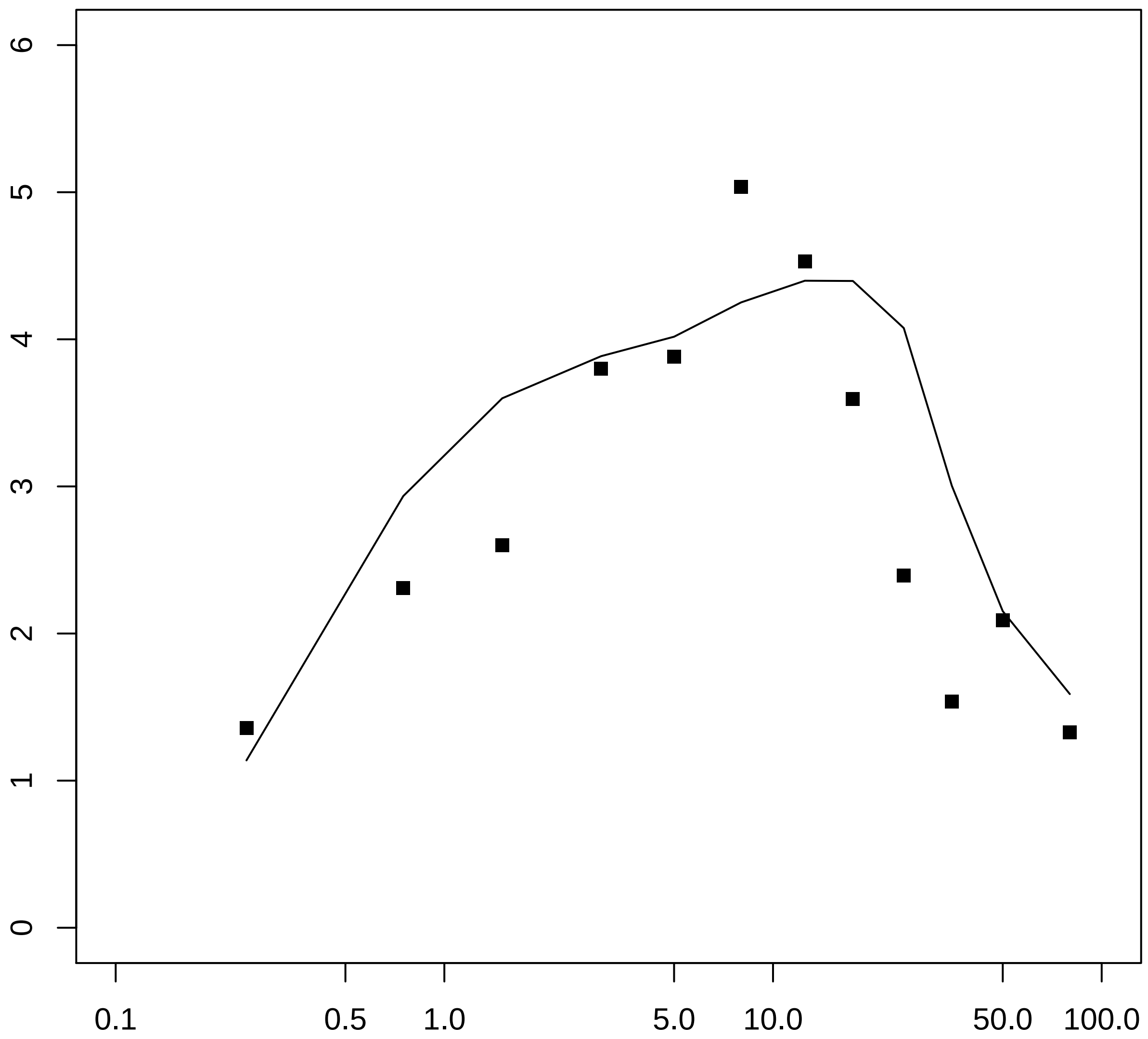

X4a: Age pattern of incidence of clinical malaria

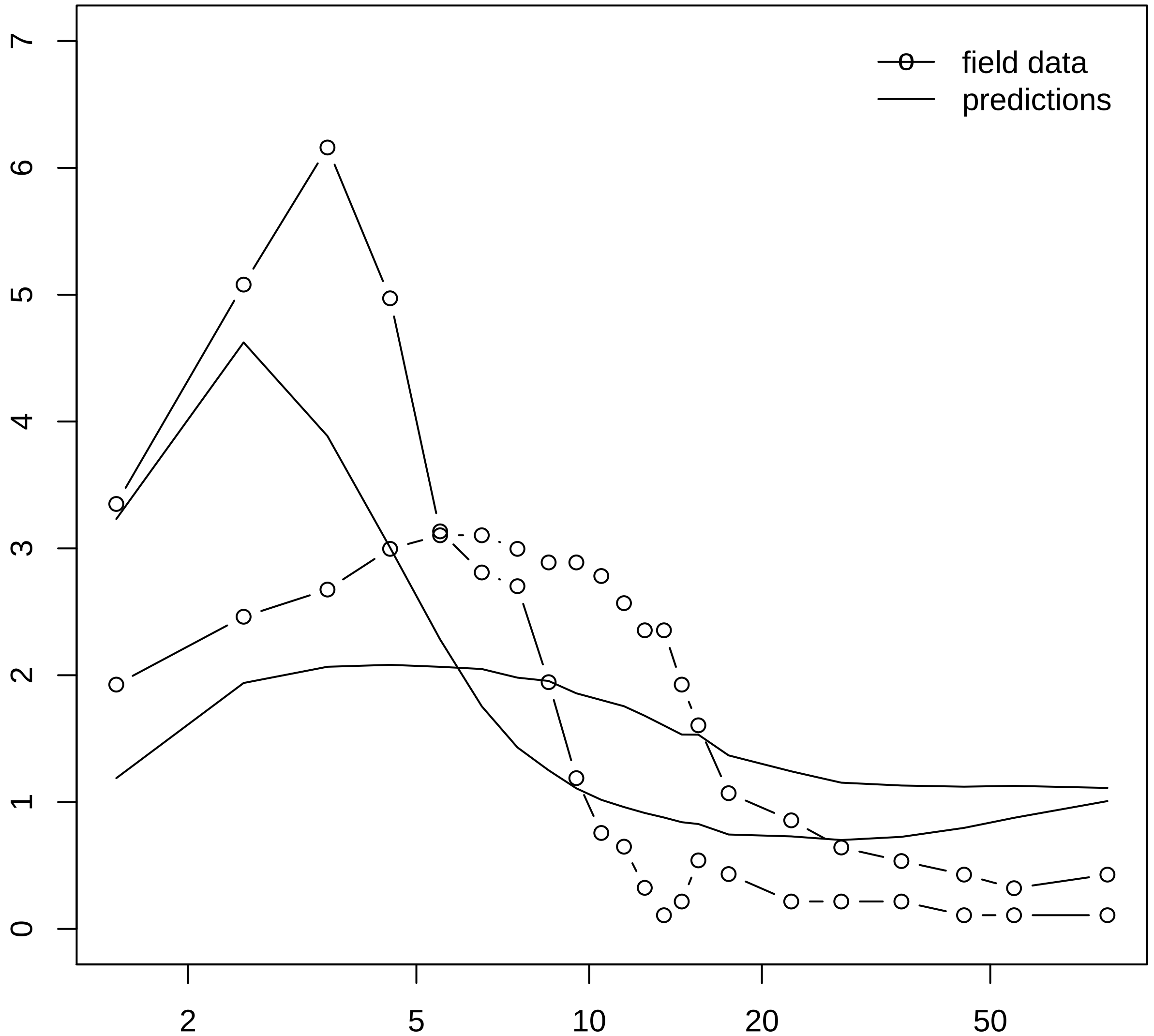

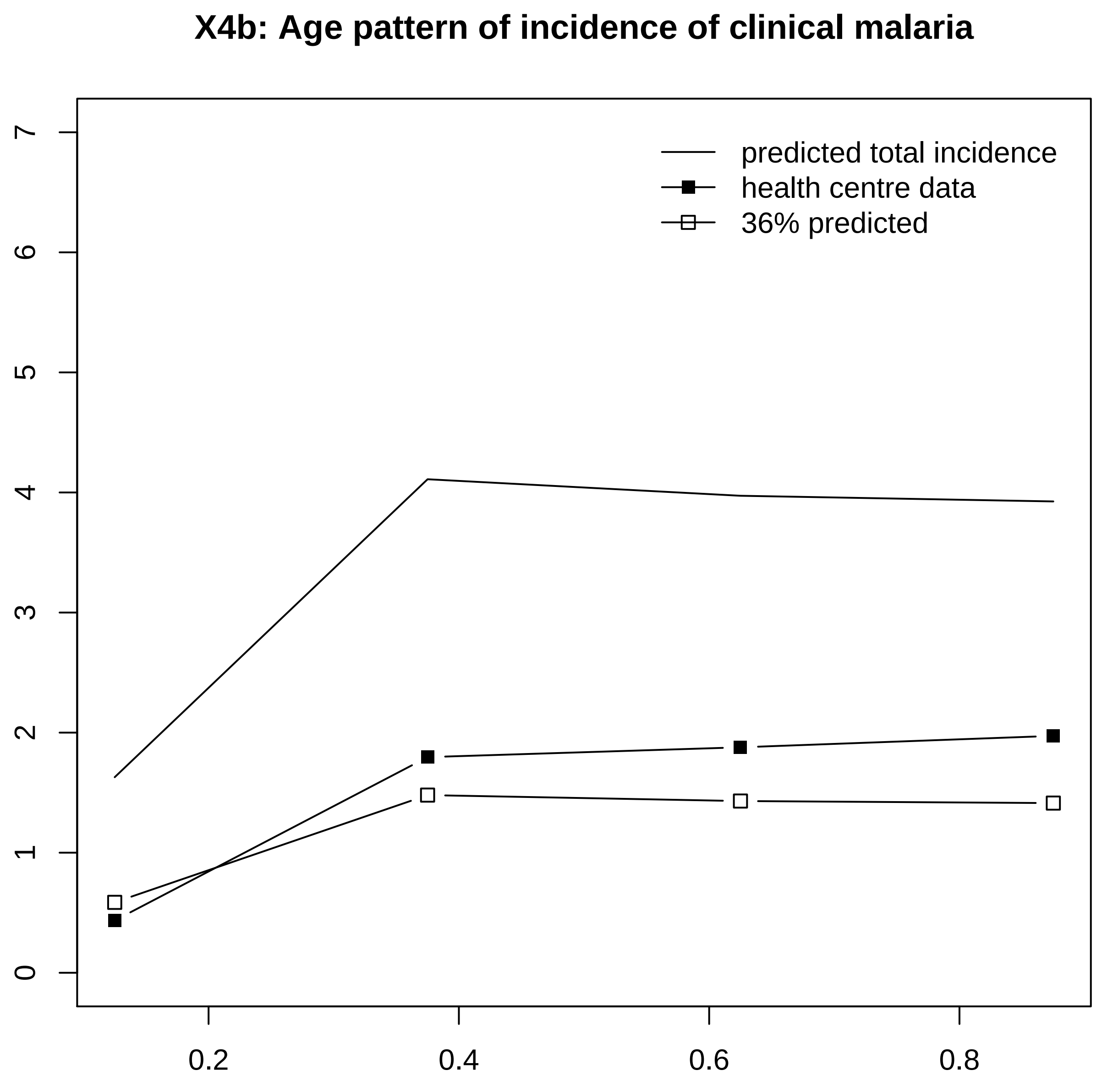

X4b: Age pattern of incidence of clinical malaria
predicted total incidence
health centre data
36% predicted
0
1
2
3
4
5
6
7
0.2
0.4
0.6
0.8

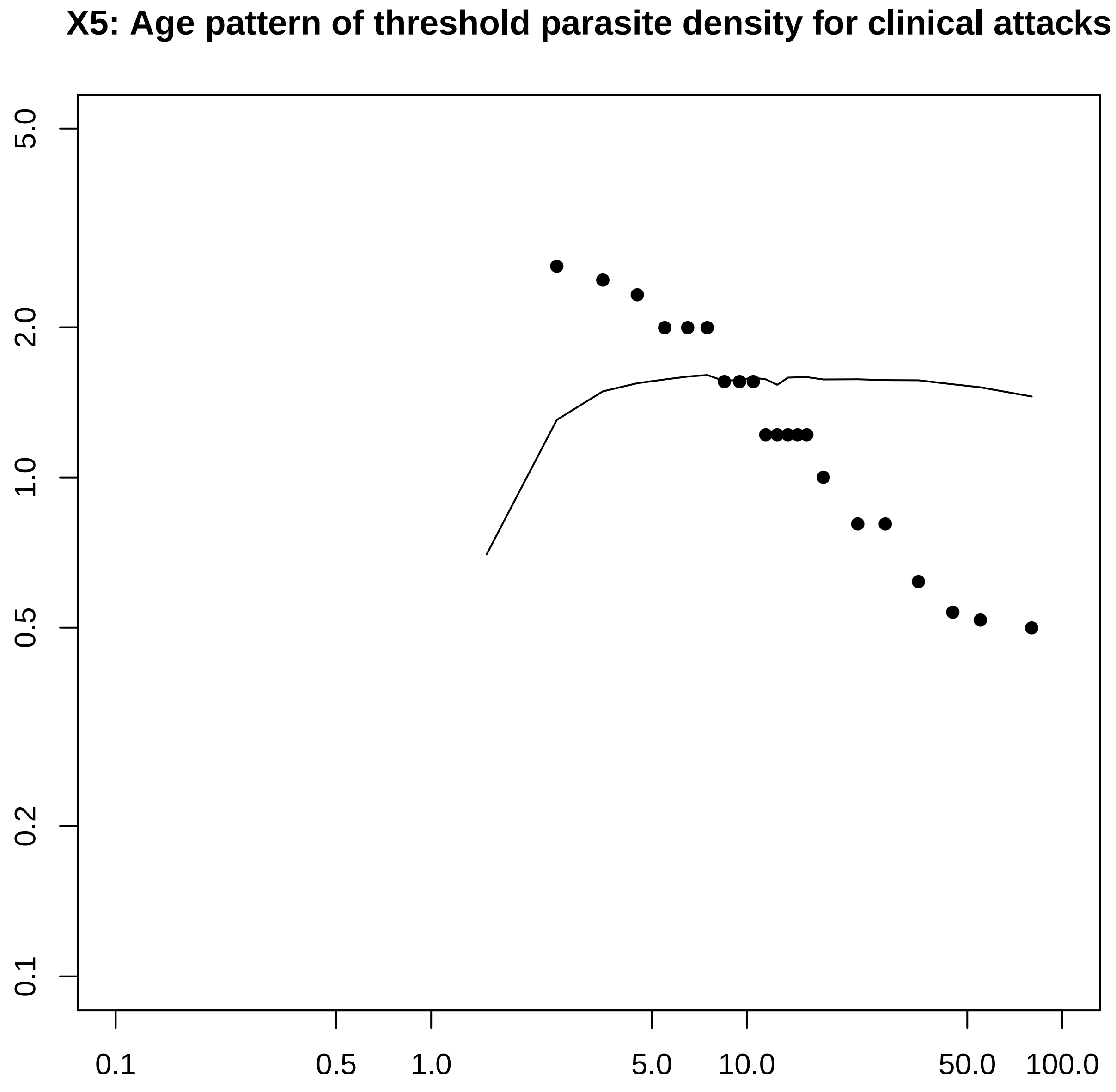
X5: Age pattern of threshold parasite density for clinical attacks
5.0
2.0
1.0
0.5
0.2
0.1
0.1
0.5
1.0
5.0
10.0
50.0
100.0

X6: Hospitalisation rate in relation to prevalence in children

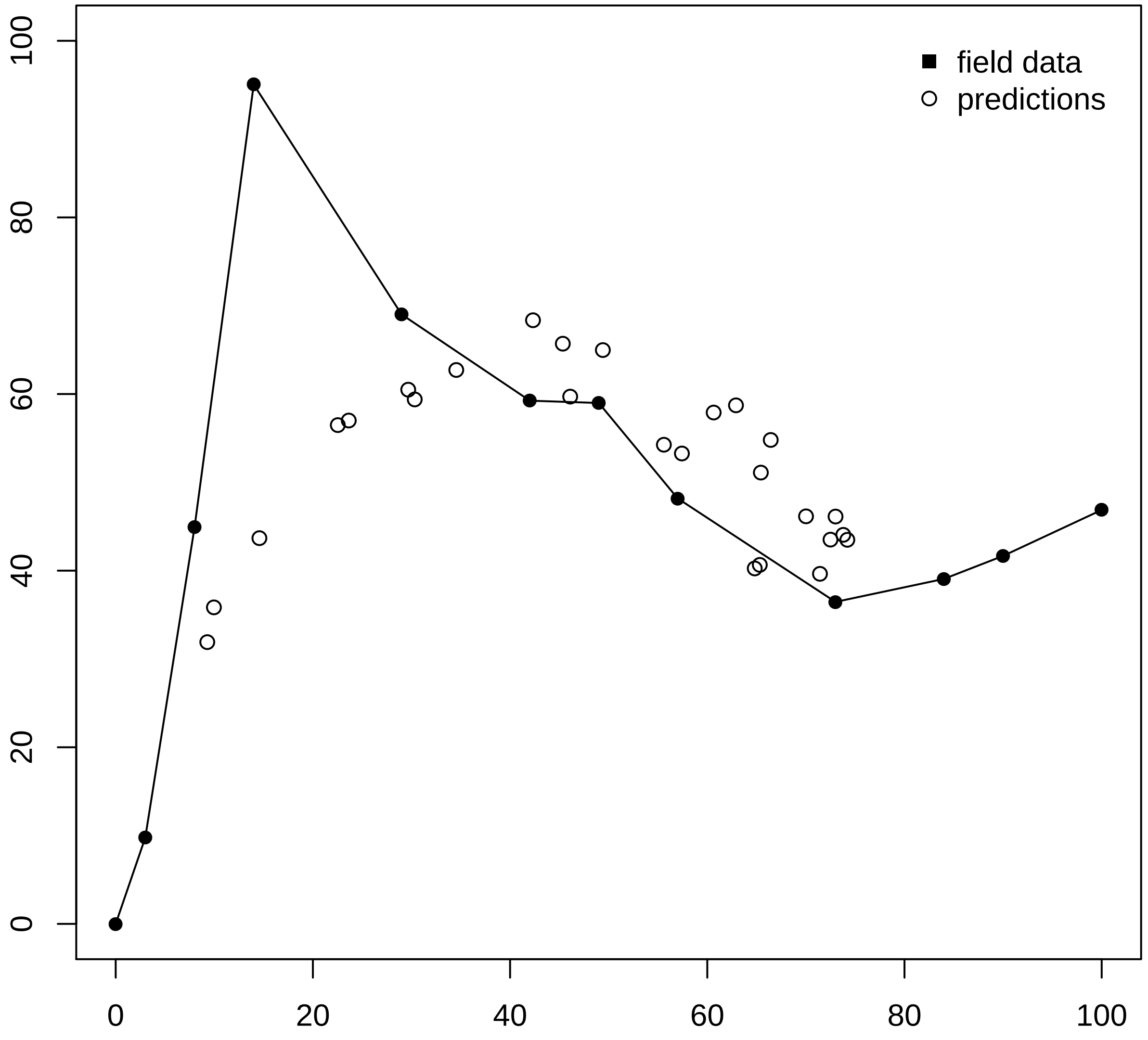

X7a: Age pattern of hospitalisation

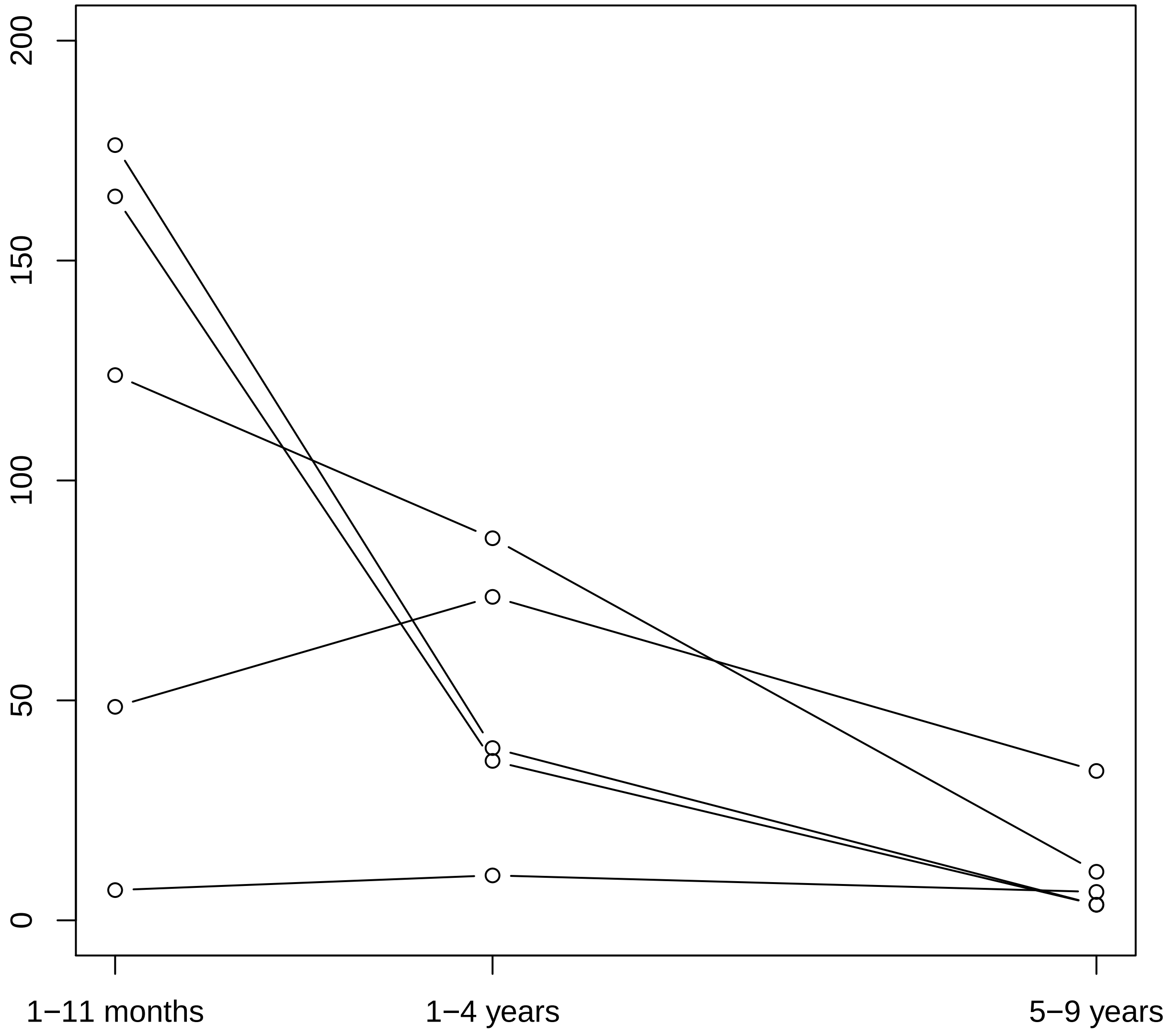

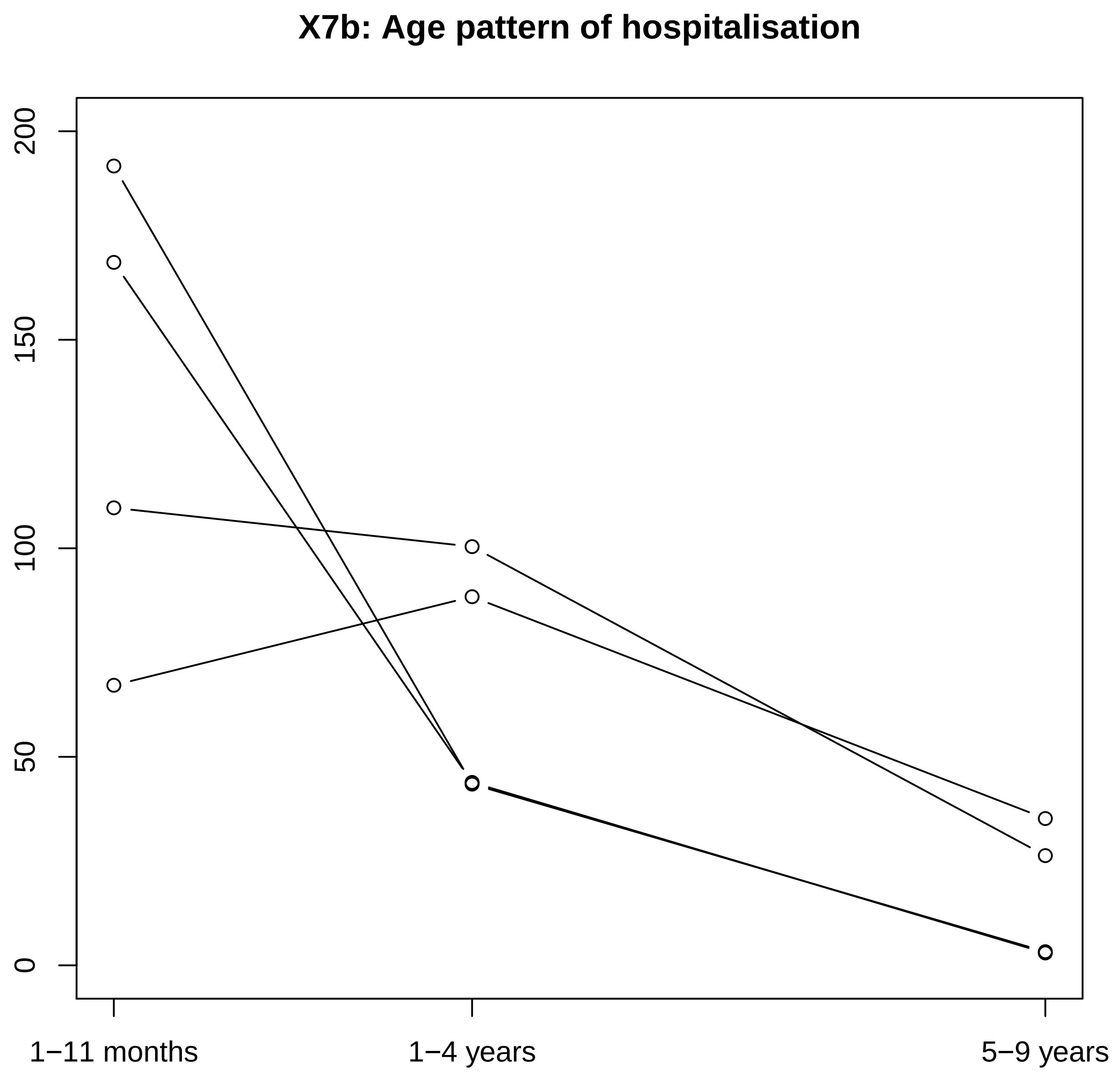
X7b: Age pattern of hospitalisation
0
50
100
150
200
1−11 months
1−4 years
5−9 years

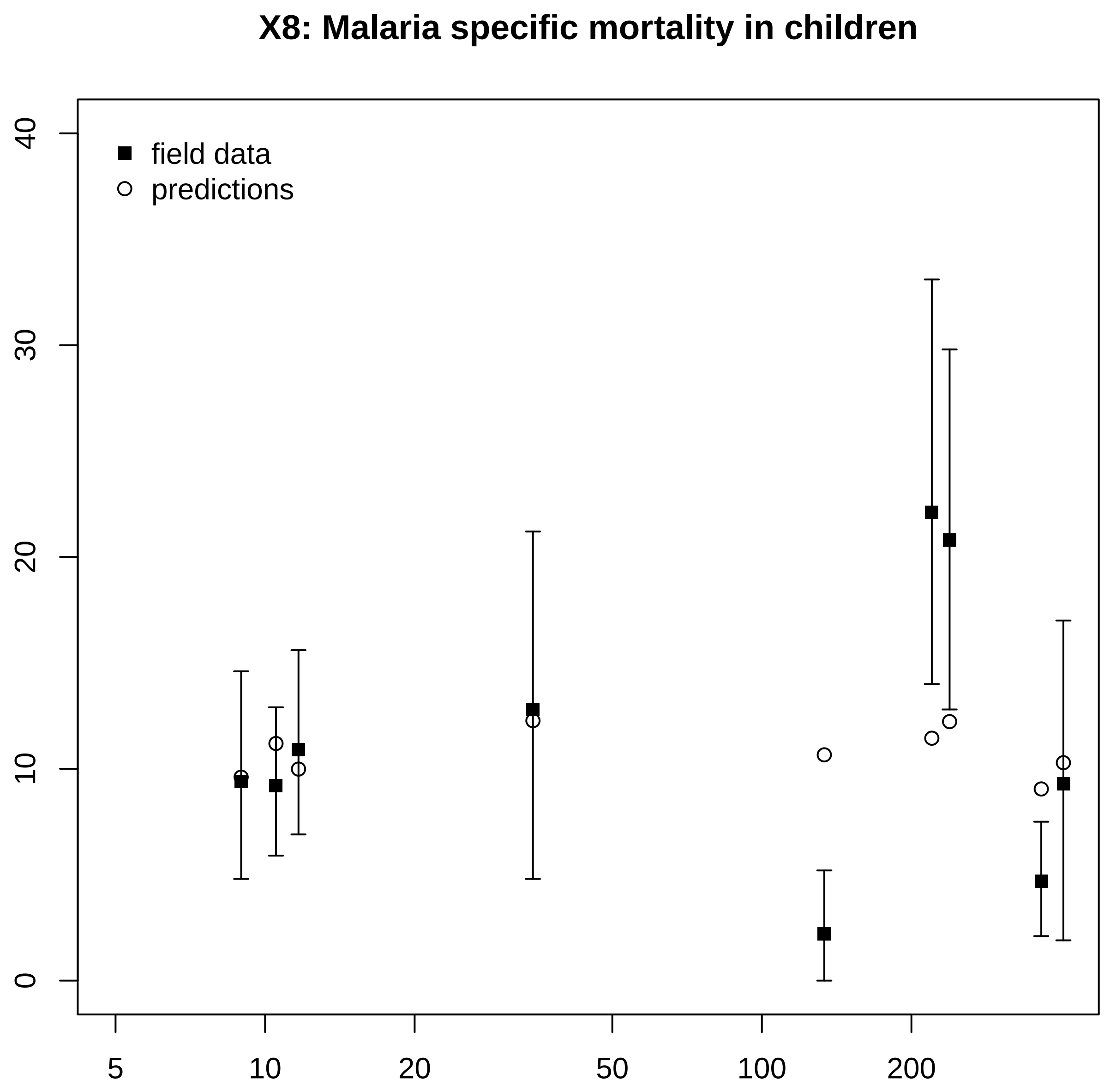
X8: Malaria specific mortality in children
field data
predictions
0
10
20
30
40
5
10
20
50
100
200

X9: Infant mortality rate

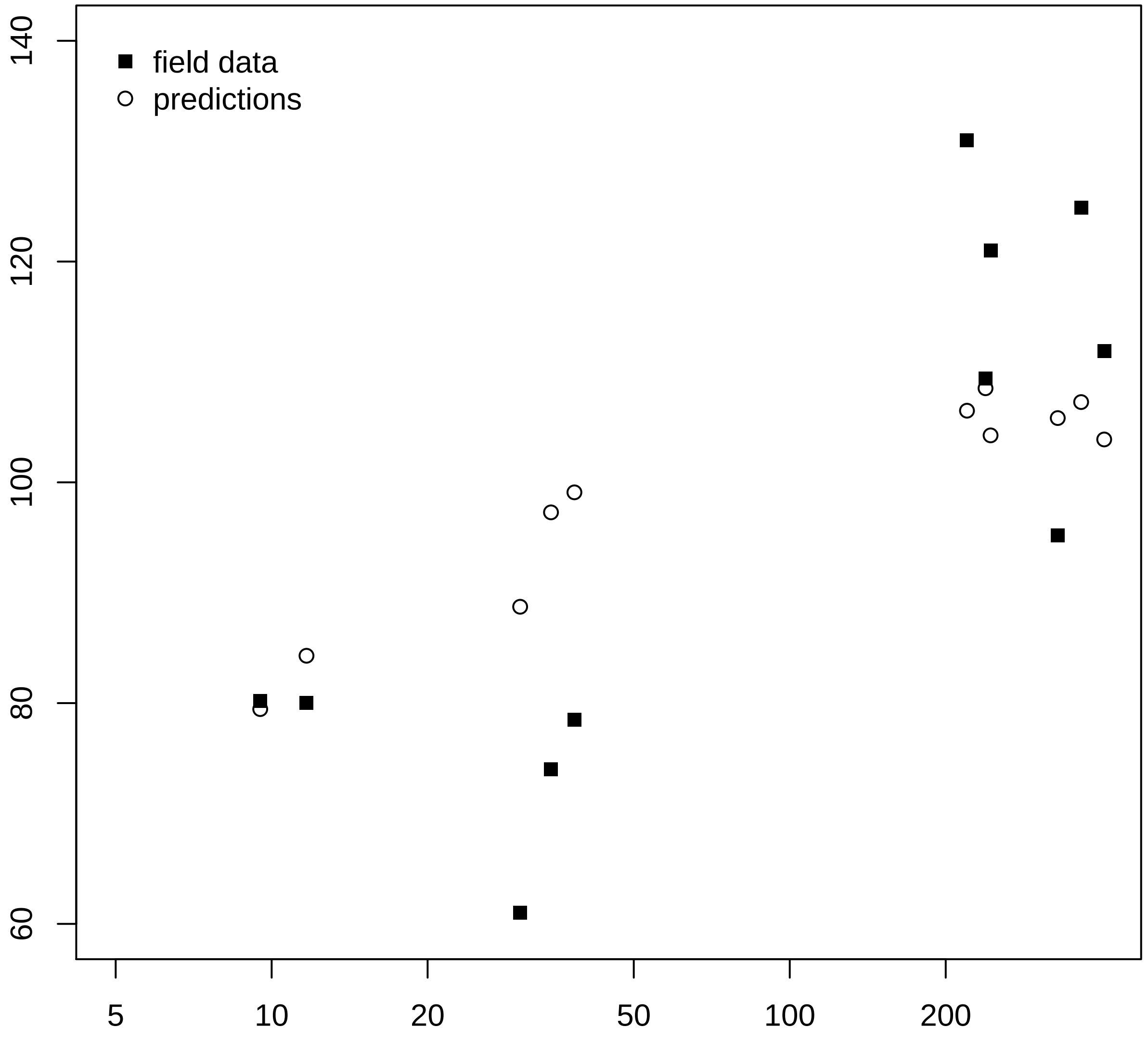

Supplement: Text S6 — Plots of model fit to field data: R0115. (PDF) [file pmed.1001157.s014.pdf]
