# Supplementary material for: Ensemble Modeling of the Likely Public Health Impact of a Pre-Erythrocytic Malaria Vaccine
Source: PLoS Med. 2012 Jan 17;9(1):e1001157. doi: 10.1371/journal.pmed.1001157 (PMC3260300; doi:10.1371/journal.pmed.1001157)
Supplement: Text S8 — Plots of model fit to field data: R0125. (PDF) [file pmed.1001157.s016.pdf]

X1: Age patterns of prevalence of infection

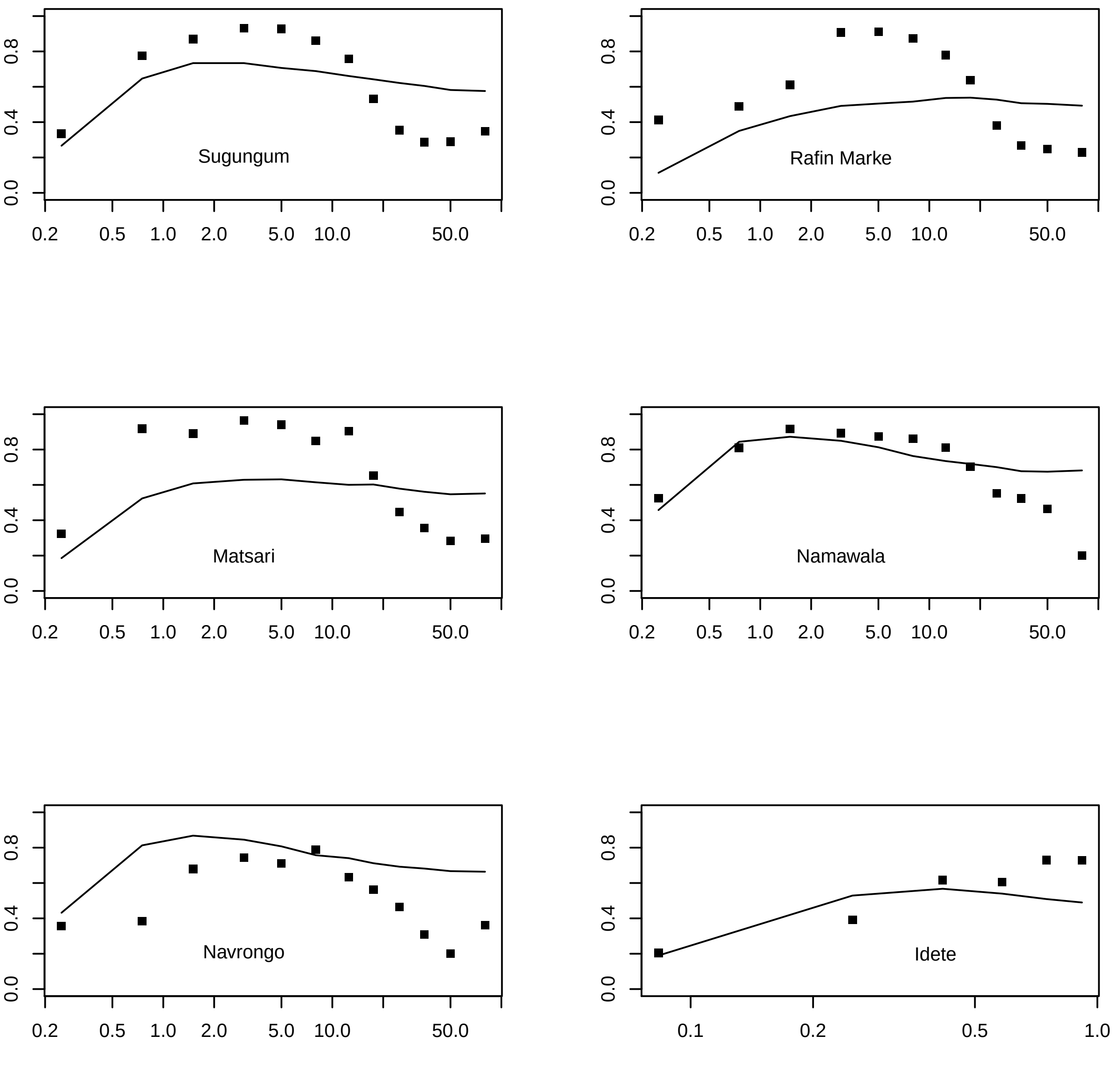

X2: Age patterns of parasite density

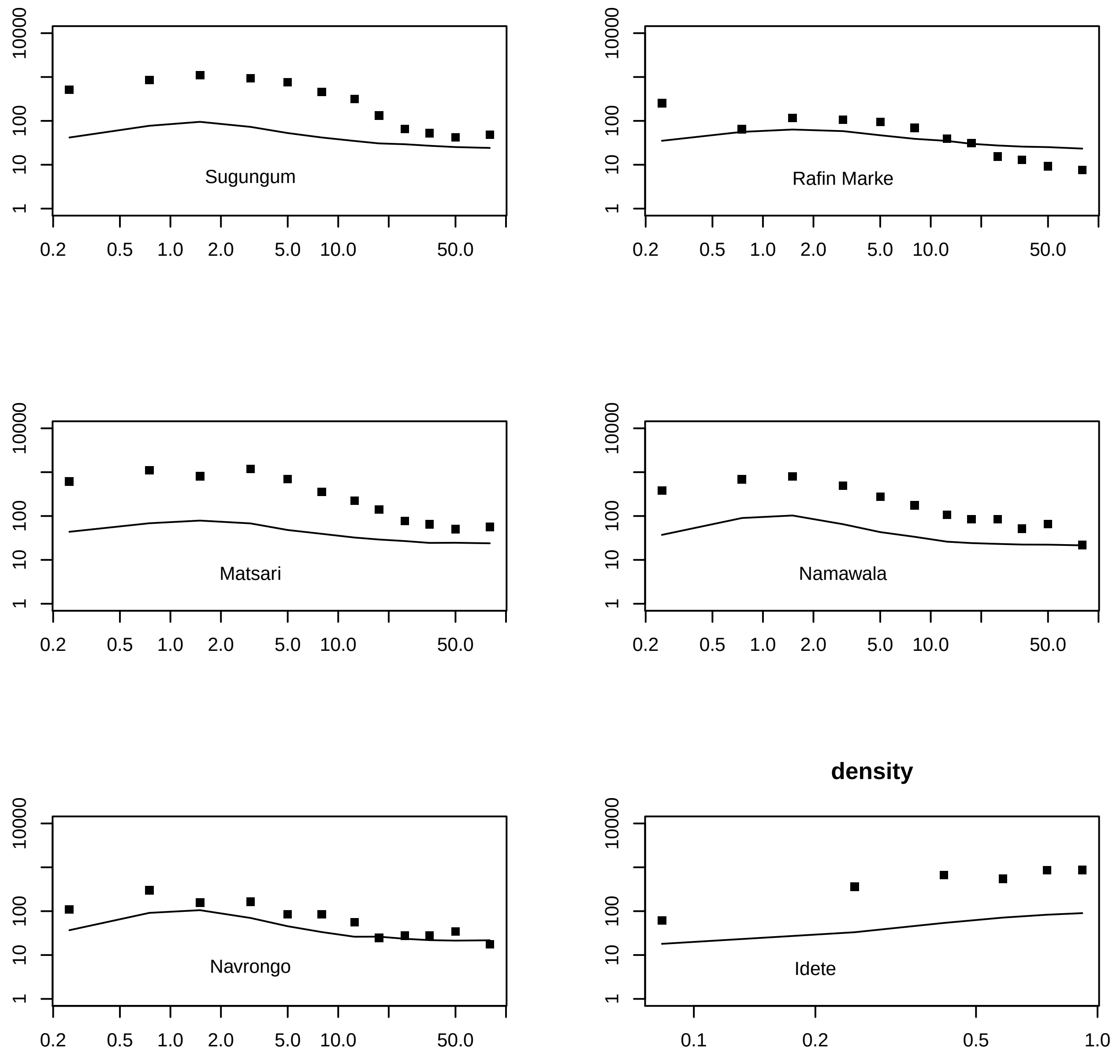

X3: Age pattern of number of concurrent infections

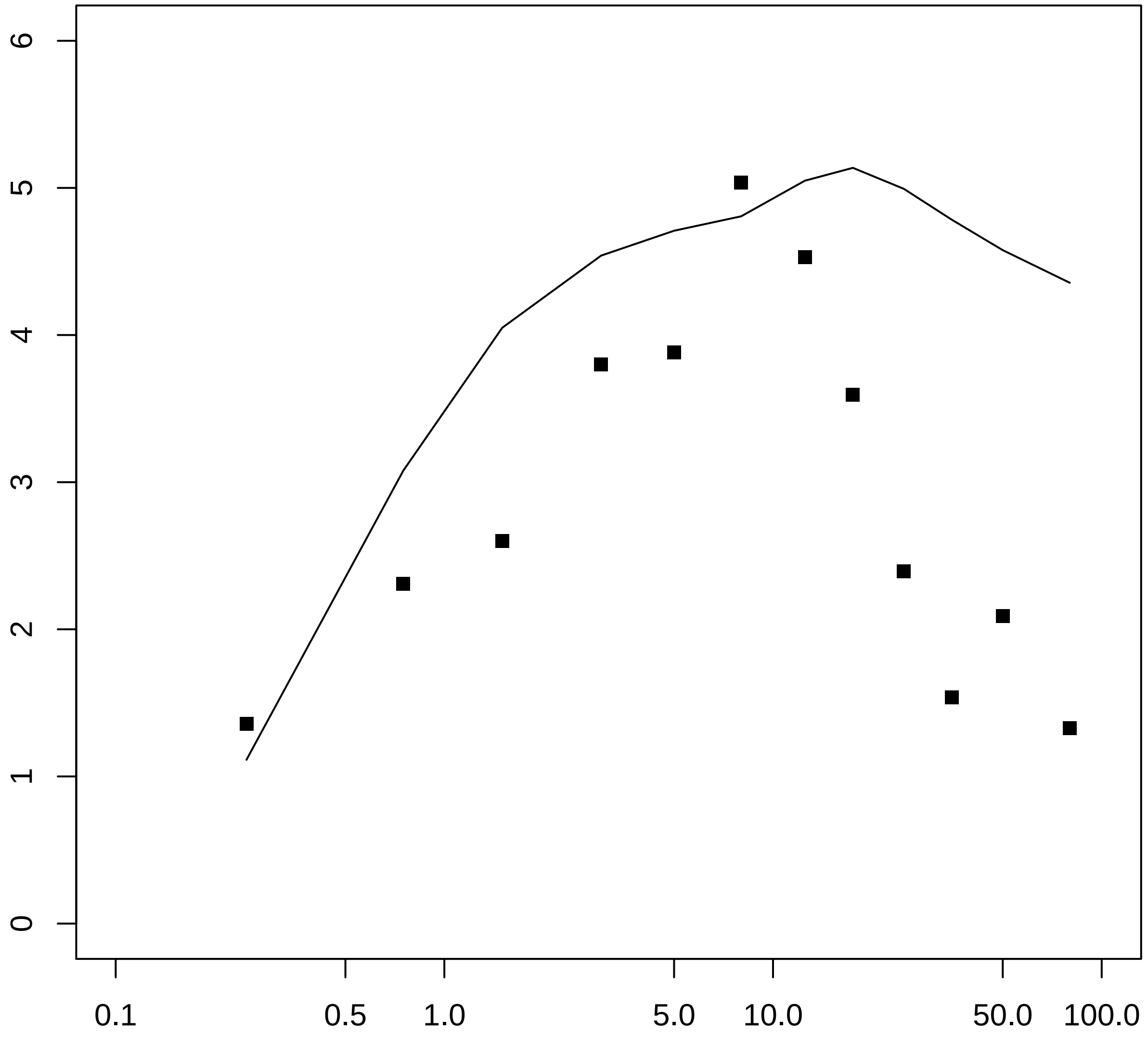

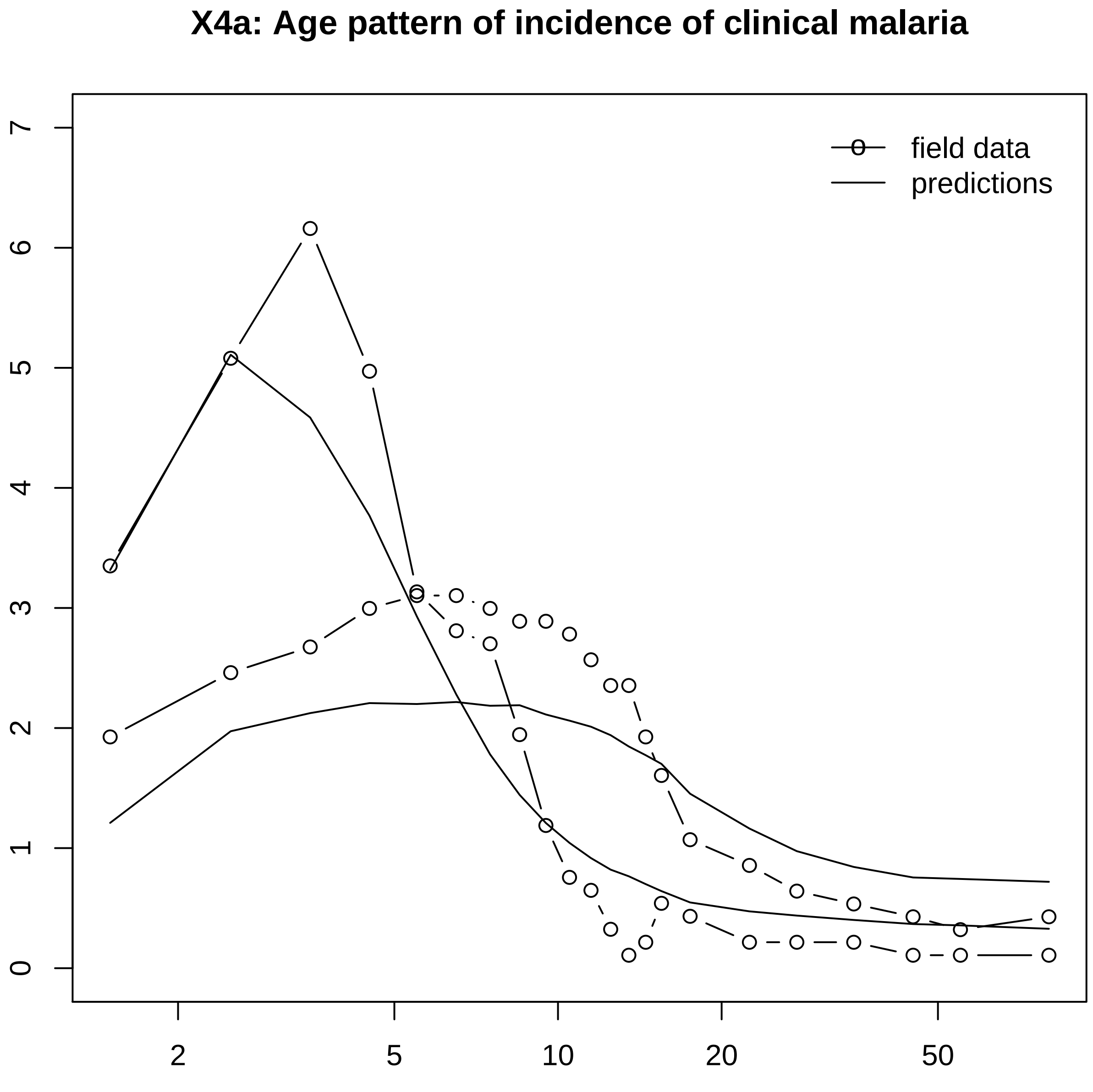
X4a: Age pattern of incidence of clinical malaria
field data
predictions
0
1
2
3
4
5
6
7
2
5
10
20
50

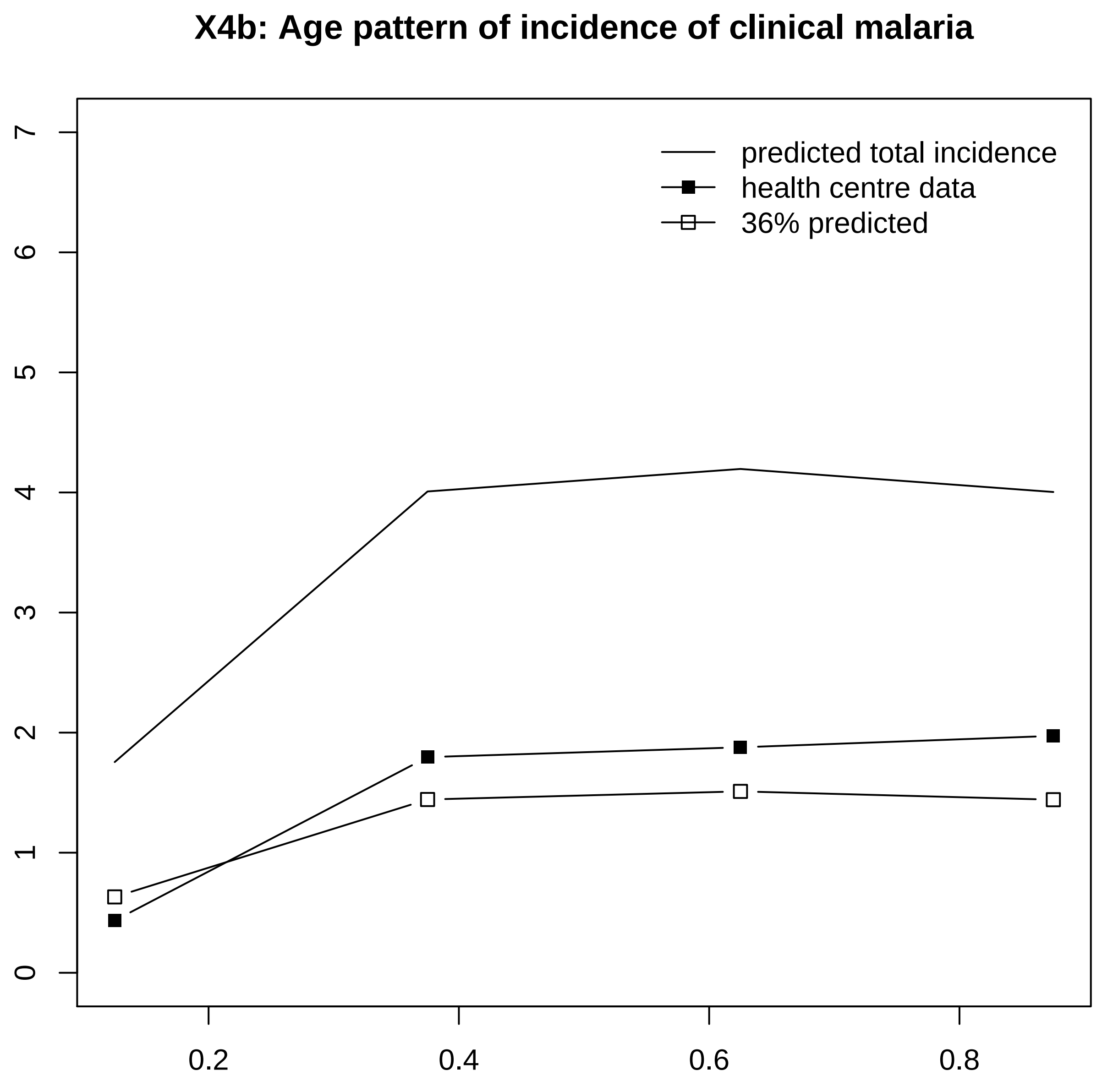
X4b: Age pattern of incidence of clinical malaria
predicted total incidence
health centre data
36% predicted
0
1
2
3
4
5
6
7
0.2
0.4
0.6
0.8

X5: Age pattern of threshold parasite density for clinical attacks

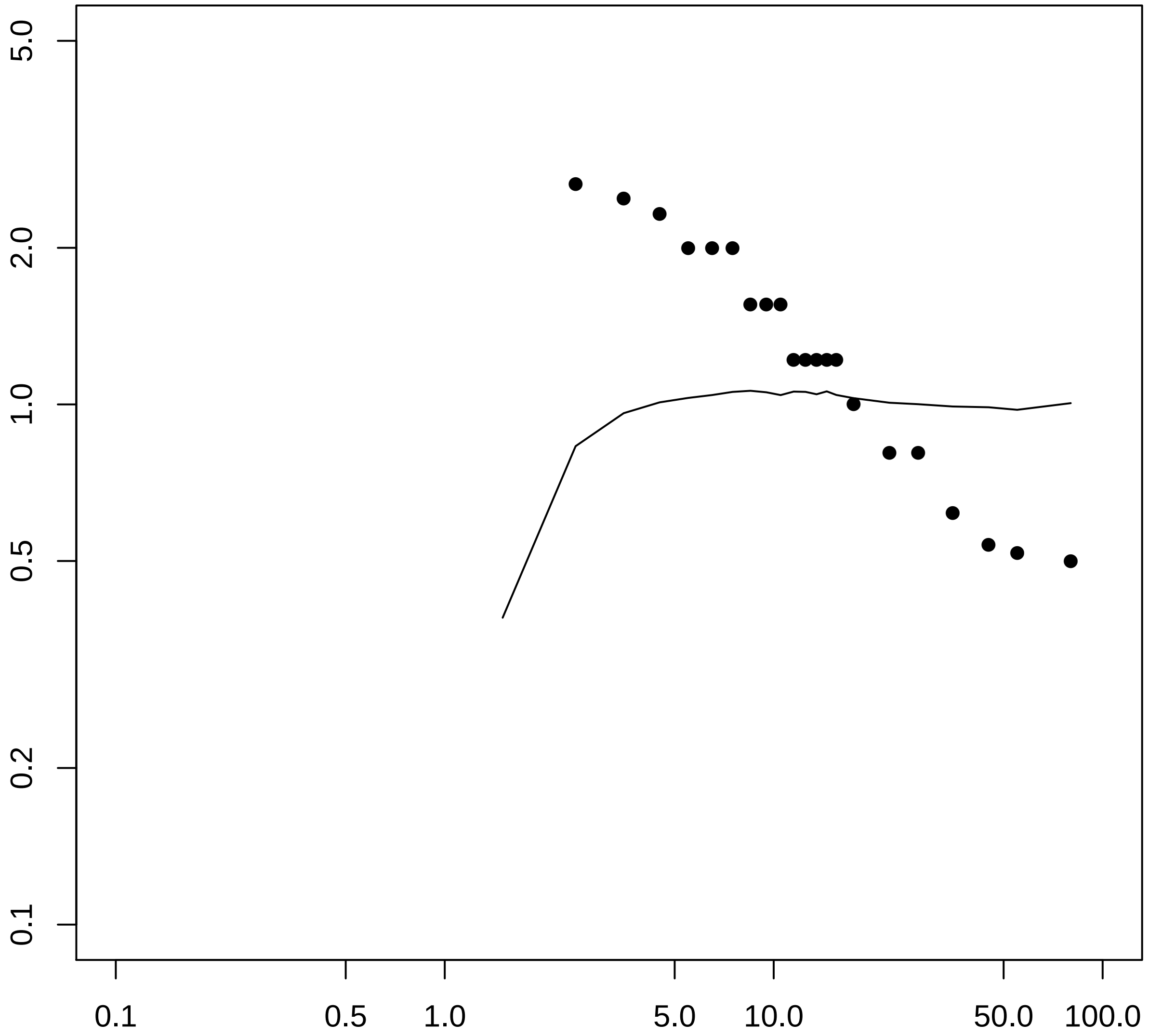

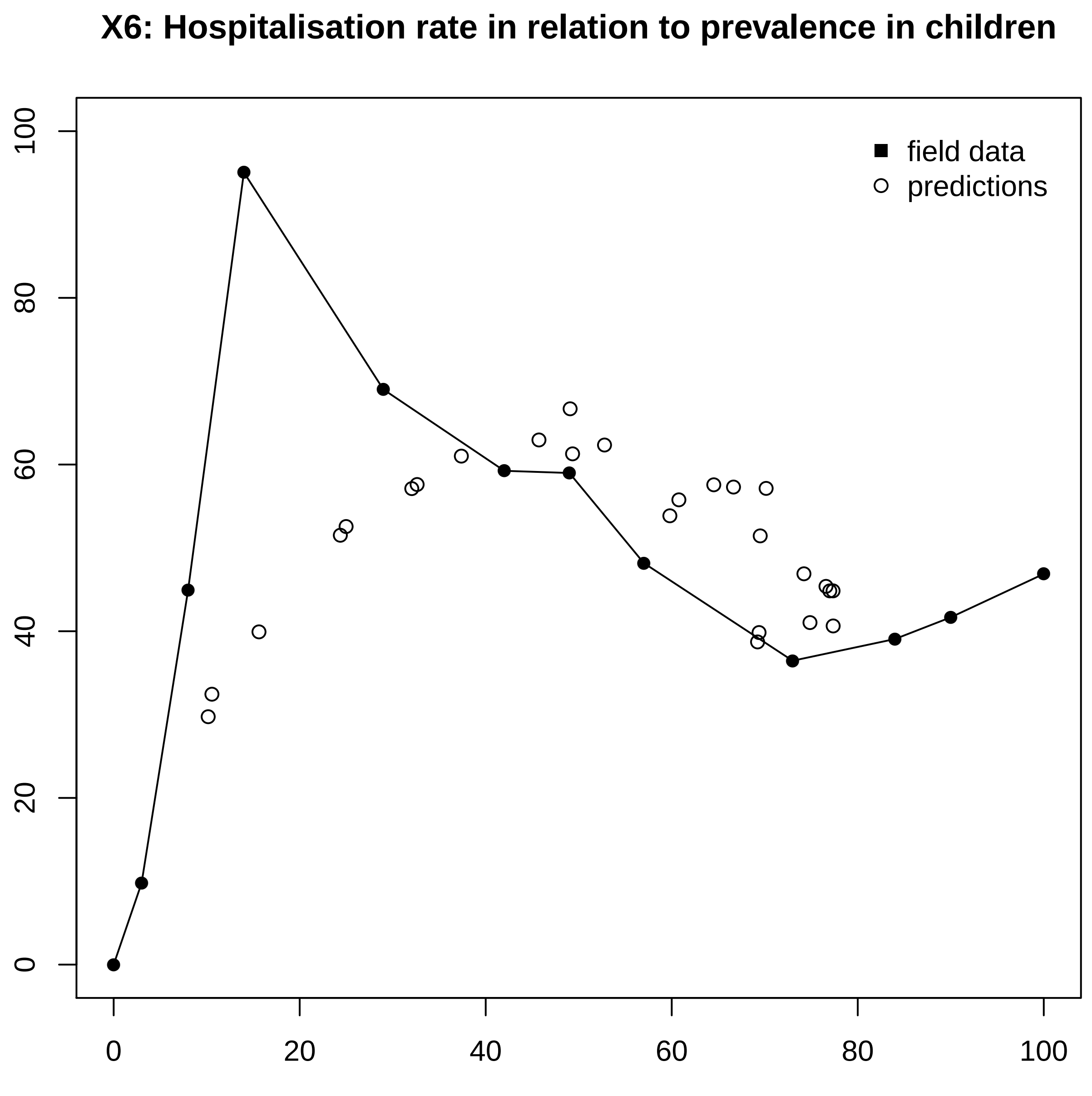
X6: Hospitalisation rate in relation to prevalence in children
field data
predictions
0
20
40
60
80
100
0
20
40
60
80
100

X7a: Age pattern of hospitalisation

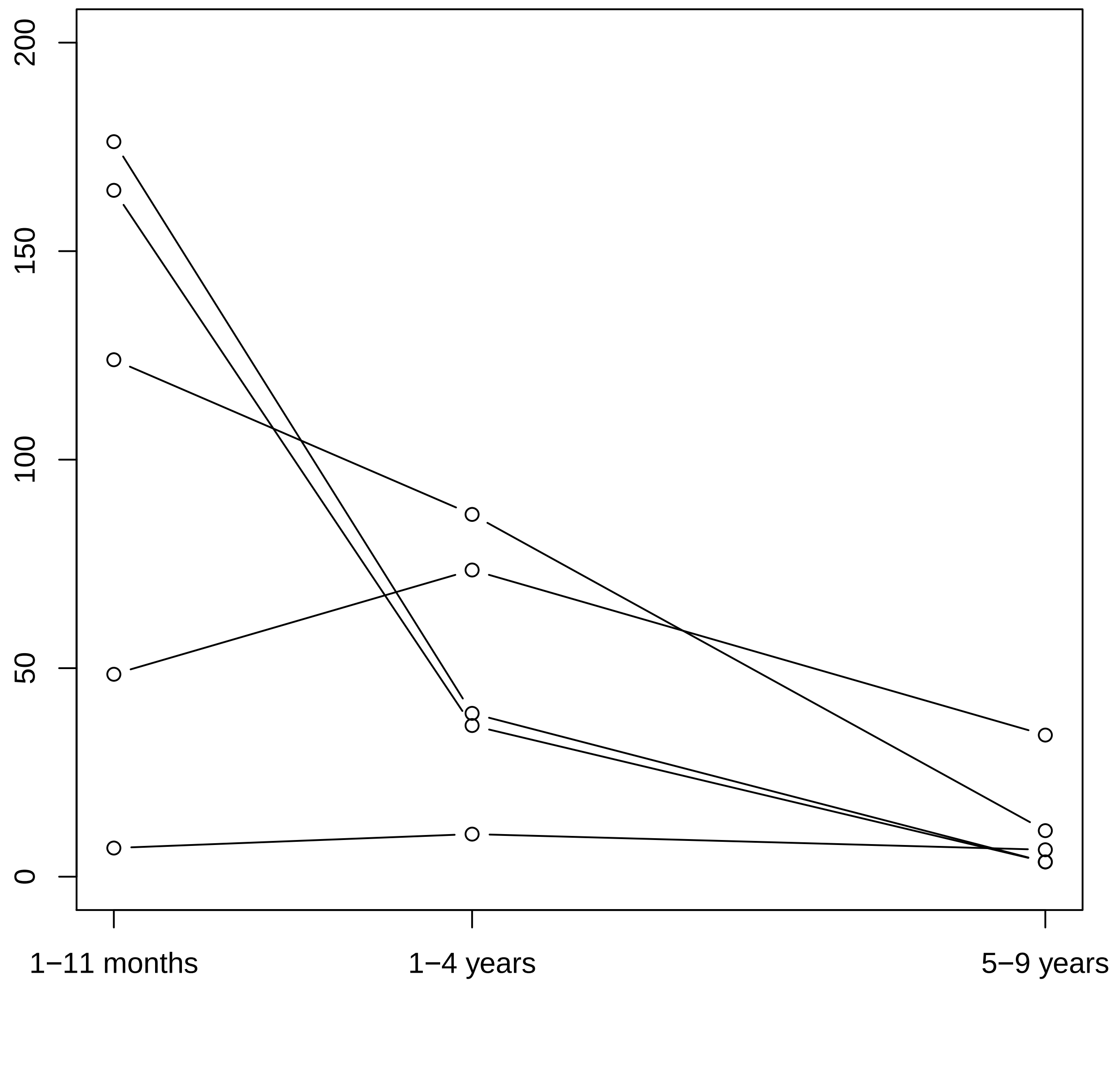

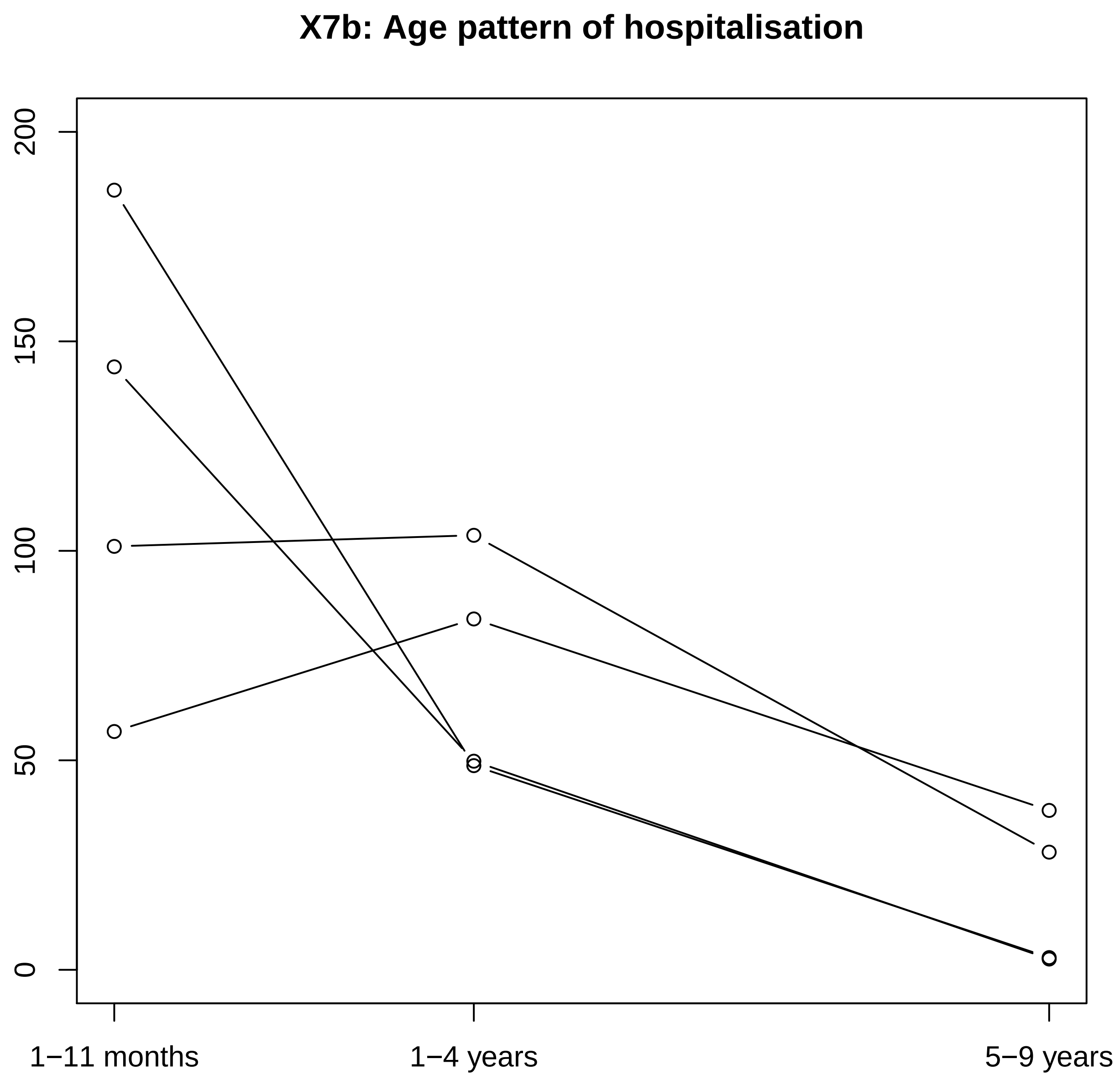
X7b: Age pattern of hospitalisation
0
50
100
150
200
1−11 months
1−4 years
5−9 years

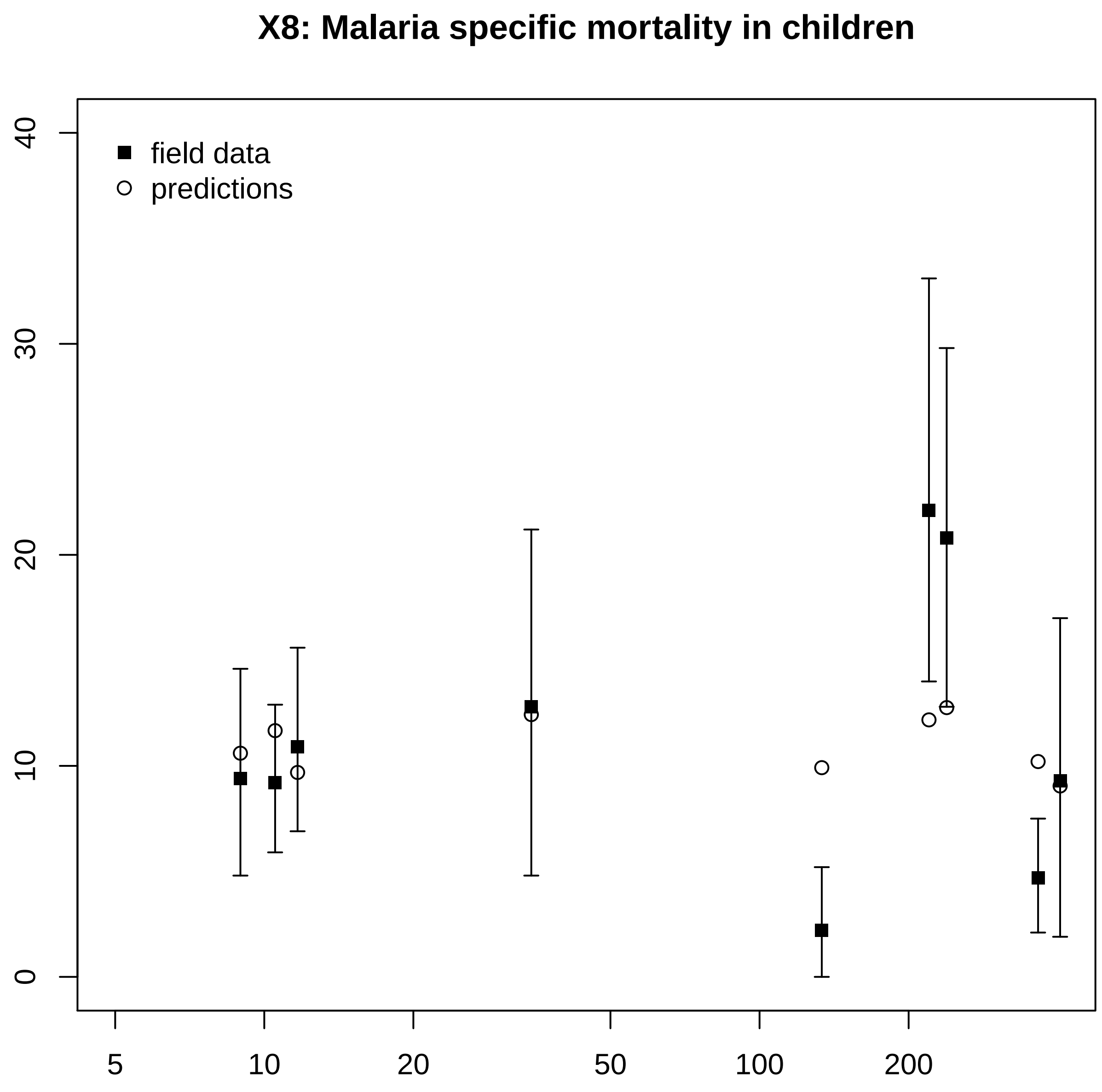
X8: Malaria specific mortality in children
field data
predictions
0
10
20
30
40
5
10
20
50
100
200

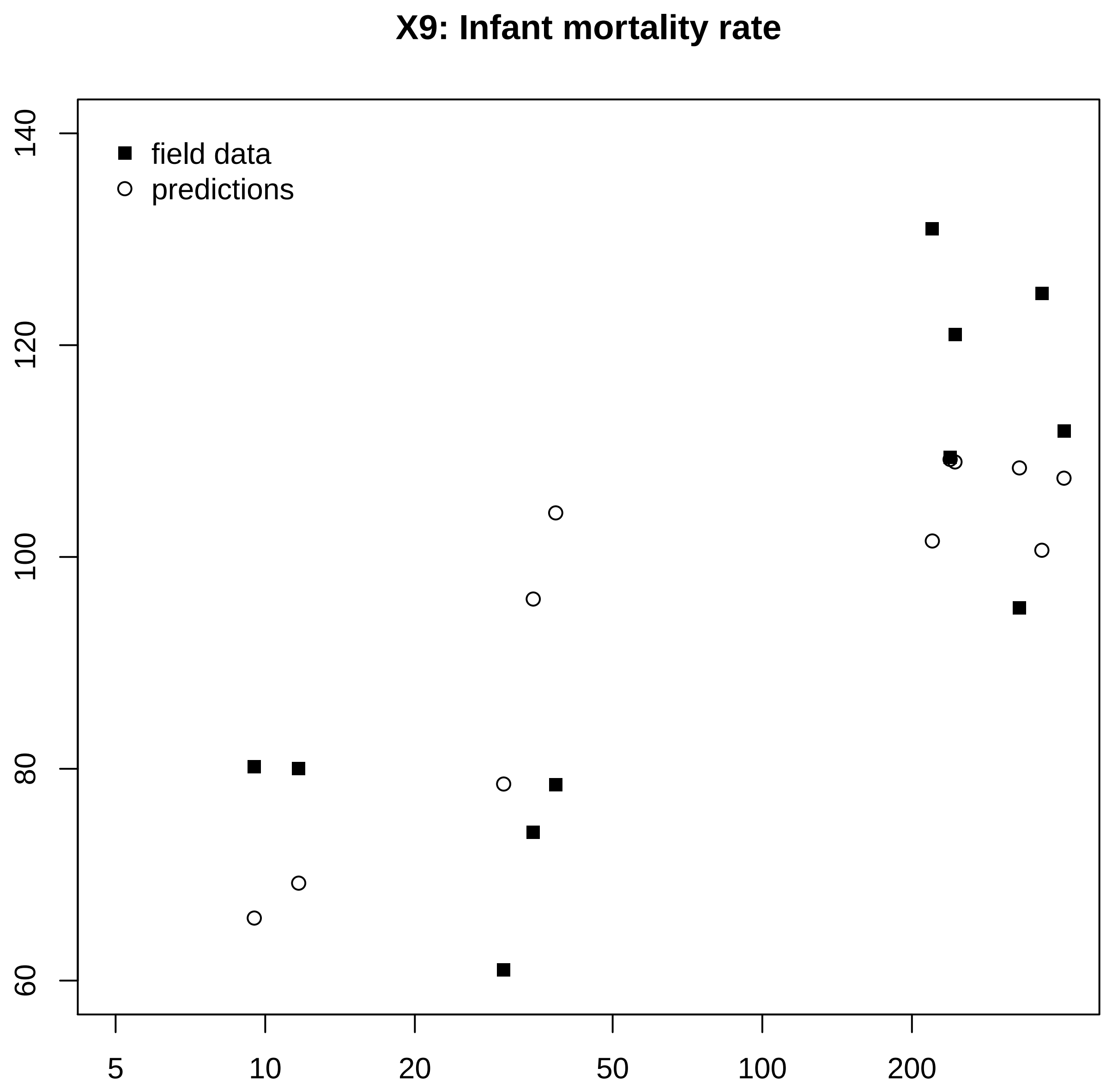
X9: Infant mortality rate
field data
predictions
60
80
100
120
140
5
10
20
50
100
200
